# Prophylactic inhibition of LATS1/2 before high-dose irradiation promotes the regeneration of the damaged intestinal epithelium

**DOI:** 10.1038/s41536-026-00496-5

**Published:** 2026-07-20

**Authors:** Lucy M. Driver, Frances Anne Tosto, Bridget S. Scherer, Lixia Luo, Katy Harbaugh, Olivia White, Ayra Charania, Jordan Tran, Rebecca Bacon, Patrick J. Morris, Michele Ceribelli, Damien Y. Duveau, Lale Evsen, Scott B. Hoyt, Keerthana Kurapati, Koravit Poysungnoen, John W. Hickey, Jatin Roper, Christine E. Eyler, Craig J. Thomas, Chang-Lung Lee

**Affiliations:** 1 Department of Pathology, Duke University, Durham, NC USA; 2 Department of Radiation Oncology, Duke University, Durham, NC USA; 3 Division of Preclinical Innovation, National Center for Advancing Translational Sciences, National Institutes of Health, Bethesda, MD USA; 4 Department of Biomedical Engineering, Duke University, Durham, NC USA; 5 Division of Gastroenterology, Department of Medicine, Duke University, Durham, NC USA; 6 Lymphoid Malignancies Branch, Center for Cancer Research, National Cancer Institute, National Institutes of Health, Bethesda, MD USA

**Keywords:** Cell biology, Gastroenterology

## Abstract

Following radiation injury, intestinal epithelial cells adapt through cellular plasticity to regenerate and repopulate the damaged epithelium by activating Yap, a key effector protein of the Hippo signaling pathway. However, the impact of pharmacologically targeting the Hippo pathway on the regeneration of the irradiated intestinal epithelium remains poorly understood. Here, we investigated this question using NCGC-023, a selective and potent small-molecule inhibitor of Yap’s upstream regulators LATS1/2. NCGC-023 treatment significantly increased active Yap protein expression in human enteroids and protected them against ionizing radiation (IR) in vitro. In mice, transient treatment with NCGC-023 before IR reduced radiation-induced DNA damage, suppressed premitotic apoptosis and aberrant mitosis in intestinal crypt cells, facilitated regenerative reprogramming of the damaged intestinal epithelium, and improved survival of mice subjected to IR doses that precipitate gastrointestinal acute radiation syndrome (GI-ARS). Mechanistically, NCGC-023 treatment before IR upregulated key signaling pathways controlled by LATS1/2, including Yap-dependent regenerative responses involving Il-33 and Yap-independent cellular responses to heavy metals mediated by metallothioneins. Moreover, NCGC-023 treatment did not exacerbate delayed injury in multiple organs of mice that survived 4 months post-irradiation. Together, these results demonstrate that transient NCGC-023 treatment before high-dose IR promotes the regeneration of the damaged intestinal epithelium. This proof-of-concept study supports further development of LATS1/2 inhibitors as prophylactic medical countermeasures for GI-ARS.

## Introduction

Exposure of the gastrointestinal (GI) tract to high-dose ionizing radiation (IR) results in acute injury of the intestinal epithelia and the development of a lethal GI acute radiation syndrome (GI-ARS)^1^. The GI-ARS is a complex process initiated by a rapid loss of crypt intestinal epithelial cells (IECs) and prolonged insufficient epithelial cell production, which leads to failure in maintaining the structure of the small intestinal epithelium and overall mucosal barrier^2^. The consequences of GI-ARS include shortening of villi and loss of the mucosal barrier that propagates profound fluid loss, electrolyte dysregulation, sepsis, and shock that ultimately can lead to death^2–4^. Due to the rapid turnover of the small intestinal epithelium, lethality from GI-ARS generally occurs within 10 days following high-dose radiation exposure due to the substantial loss of IECs^3,4^. Supportive therapies remain the only interventions available for GI-ARS, highlighting the unmet need to develop novel medical countermeasures that protect or mitigate GI-ARS by promoting intestinal epithelial regeneration^5–7^.

One key signaling node mediating intestinal regeneration following tissue injury is the Hippo signaling pathway, whose activity is governed by the core transcriptional co-activator Yap (*Yap1*) and its paralog Taz (*Wwtr1*)^8–10^. The Hippo pathway is a key signaling cascade central to intestinal tissue homeostasis and coordinating regenerative responses^9–19^. Under homeostatic conditions, active Yap protein is restricted to Lgr5^+^ intestinal stem cells (ISCs) at the base of the crypt^13,20^. Precise regulation of Yap activity is essential for intestinal tissue maintenance, as constitutive *Yap* overexpression drives aberrant reprogramming of differentiated epithelial cells into a pluripotent state, which is unable to subsequently differentiate into the various component cells of the intestine^12,20,21^. In contrast, following intestinal injury, Yap is robustly activated and is required for effective tissue repair and regeneration^11,19,21–29^. For example, the *Yap* gene signature is significantly enriched in the GI epithelium in response to dextran sodium sulfate (DSS) and radiation injury^24,29,30^. Notably, the regenerative responses to such insults are significantly impaired in mice harboring IEC-specific knockout of *Yap*^9,13,23,24,28,30,31^.

The activity of Yap and its paralog Taz is negatively regulated by the core Hippo kinases, large tumor suppressor kinases 1 and 2 (LATS1/2), which phosphorylate Yap and/or Taz to promote cystolic retention and transcriptional inhibition of Yap and/or Taz targets^8,15,16,32^. Inhibition of LATS1/2 permits nuclear translocation of Yap and/or Taz and activation of transcriptional networks regulating self-renewal, cell cycle control, cell survival, and DNA damage repair^14,18,27,33^. Consistent with this, pharmacologic inhibition of LATS1/2 improves wound healing of the skin and promotes regeneration of cardiac tissue^34–39^. However, the impact of such an approach on acute and delayed radiation-induced intestinal injury remains unknown. Here, we characterized a small-molecule inhibitor of LATS1/2 and demonstrated that transient LATS1/2 inhibition prior to high-dose radiation promotes epithelial reprogramming of small intestinal IECs and regeneration of crypts in both mice and intestinal organoids through Yap-dependent and Yap-independent mechanisms.

## Results

### Characterization of the LATS1/2 inhibitor NCGC-023

To evaluate the radioprotective effects of small molecule modulators of the Hippo signaling pathway, we characterized a recently described LATS1/2 kinase inhibitor, NCGC00846023, hereafter referred to as NCGC-023 (Supplementary Fig. 1) (listed as compound #28 in Patent WO2023239727)^40^. NCGC-023 exhibited high potency against LATS1 and LATS2 proteins as assessed by in vitro kinase assays (γ-^33^P assay), with low nM IC_50_ against both kinases (IC_50_ = 14.1 nM and 14.7 nM for LATS1 and LATS2, respectively) and robust LATS1/2 inhibition in cell-based NanoBRET target-engagement assays (IC_50_ = 367 nM and 57 nM for LATS1 and LATS2, respectively) (Supplementary Table 1 and Supplementary Fig. 2). In addition, the cellular IC_50_ of NCGC-023 against LATS1 and LATS2 is superior to that of previously published LATS1/2 inhibitors, including TRULI^35^, TDI-011536^34^, VT02956^41^, and NIBR-LTSi^37^ (Supplementary Table 2). Furthermore, we examined the selectivity of NCGC-023 across the kinome using both cell-free and physiologically relevant cell-based approaches. In a 370-kinase recombinant panel, only 22 kinases exhibited IC_50_ values < 50 nM in vitro, 4 of which were Hippo pathway-related hits, including LATS1 and LATS2. Complementary chemoproteomic profiling in HEK293 lysates showed that among 310 kinases, only 6 were inhibited >50%, with LATS1 and LATS2 as the top hits, demonstrating robust and selective target engagement (Supplementary Data 1).

To assess the effect of NCGC-023 on Yap-mediated signaling, we treated HepG2 human hepatocellular carcinoma cells with NCGC-023 in vitro for 4 h and observed significant induction of transcriptional targets of *Yap1*, including *Amotl2*, *Ctgf*, and *Cyr61* (Supplementary Fig. 3). In addition, acute intraperitoneal (IP) in vivo dosing of NCGC-023 markedly inhibited Yap Ser397 phosphorylation in mouse livers up to 8 h after treatment (Supplementary Fig. 4). Pharmacokinetic analysis showed the C_max_ of NCGC-023 was 2616 ng/mL and 7593 ng/mL in mice treated with NCGC-023 at 3 mg/kg and 10 mg/kg, respectively, following IP administration (Supplementary Fig. 5). Together, these results indicate that NCGC-023 is a highly potent and selective LATS1/2 inhibitor that effectively activates Yap protein in vitro and in vivo.

### Transient NCGC-023 treatment protects human enteroids from radiation in vitro

**Fig. 1:**
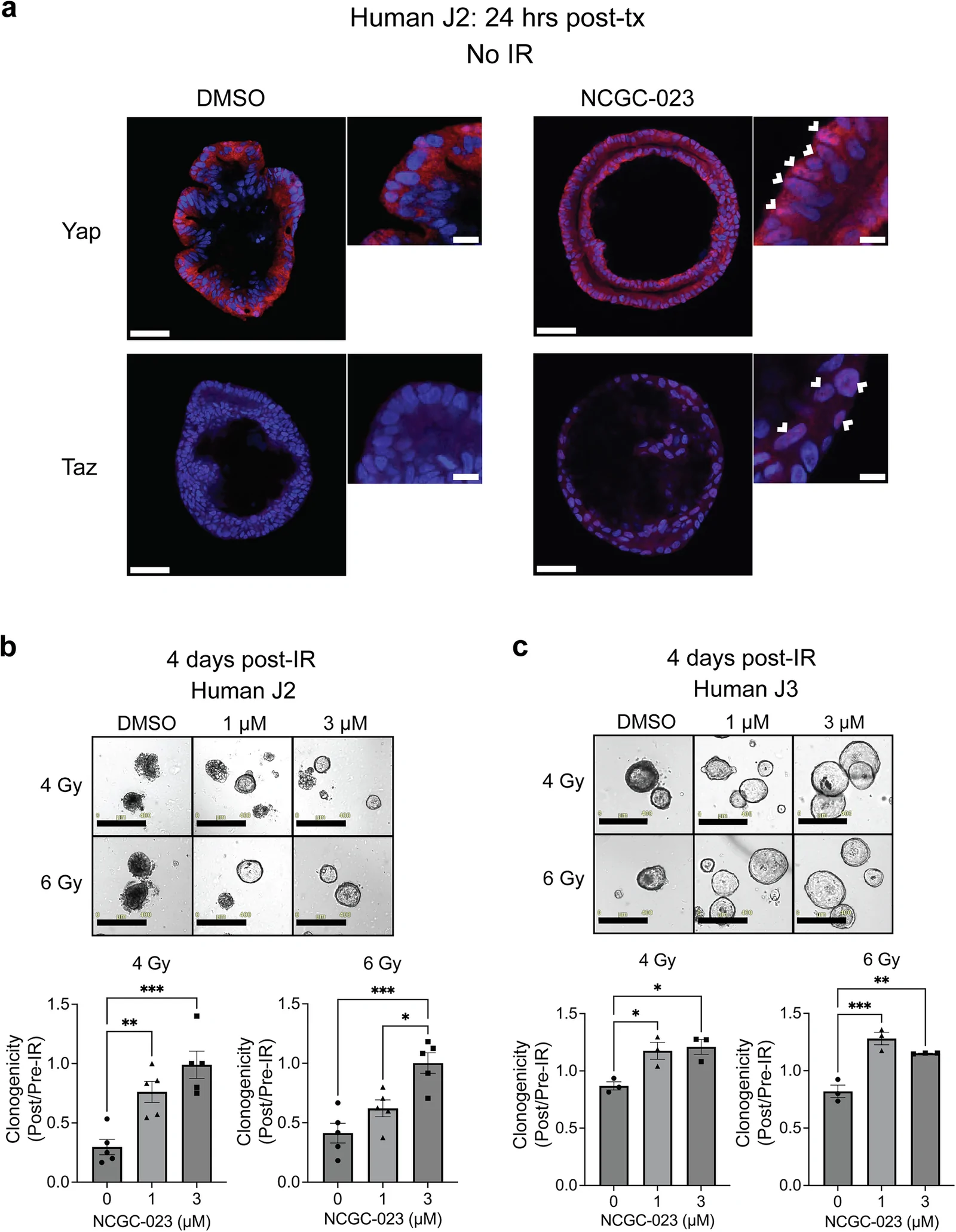
Transient NCGC-023 treatment protects human enteroids from radiation-induced death. **a** Human enteroids (J2) treated with either DMSO or 3 µM NCGC-023 24 hours (h) prior to fixation, stained with either Yap or Taz (red) and counterstained with DAPI (blue). Scale bar 50 µm and 10 µm (zoomed in). **b** Human J2 enteroids were treated with DMSO, 1 µM NCGC-023, or 3 µM NCGC-023 for 24 h prior to 4 or 6 Gy X-rays and were examined 4 days post-IR. Scale bar 400 µm. Quantification of enteroid clonogenicity after 4 or 6 Gy IR. Each dot represents a single well, *n* = 5 wells. **c** Human J3 enteroids were treated with DMSO, 1 µM NCGC-023, or 3 µM NCGC-023 for 24 h prior to 4 or 6 Gy X-rays and were examined 4 days post-IR. Scale bar 400 µm. Quantification of enteroid clonogenicity after 4 or 6 Gy IR. Each dot represents a single well, *n* = 3 wells. *p < 0.05, **p < 0.01, ***p < 0.001 by one-way ANOVA, followed by Tukey’s post hoc test for pairwise comparisons. Error bars represent SEM.

Human enteroid models recapitulate the cellular hierarchies evident in intact intestines in vivo and provide an advanced tool to elucidate the role of IECs in intestinal regeneration and pathophysiology in vitro^42^. To assess the impact of LATS1/2 inhibition (LATS1/2i) and activation of LATS1/2i downstream targets by NCGC-023, we treated a human jejunal enteroid line (J2)^43–45^ with NCGC-023 at 3 µM for 48 h. NCGC-023 treatment resulted in substantial nuclear localization of Yap protein in immunofluorescent (IF) whole mount staining, whereas the expression of Taz protein was only weakly evident in this organoid line (Fig. 1a).

We next investigated the impact of NCGC-023 treatment on the radiation response of human enteroids. Based on published studies, enteroids were challenged with 4 or 6 Gy X-rays^46^ along with NCGC-023 or DMSO treatment given before or after IR. Our results showed that in two human jejunal enteroid lines, J2 and J3^43–45^, transient NCGC-023 treatment with 1 and 3 μM 24 h prior to radiation significantly improved clonogenicity after 4 and 6 Gy, respectively (Fig. 1b, c). Morphological analysis of irradiated DMSO-treated enteroids appeared structurally collapsed and compacted due to the loss of lumen integrity and poorly defined borders filled with cellular debris, indicative of enteroid death as described previously^42,47,48^. In contrast, NCGC-023-treated enteroids appeared larger with clearly demarcated borders and transparent lumens, indicative of preserved cellular viability (Fig. 1b, c). Furthermore, transient treatment of unirradiated human enteroids with NCGC-023 1 and 3 μM for 24 h did not result in significant growth or morphological changes compared to DMSO controls, demonstrating negligible aberrant growth phenotypes with NCGC-023 treatment under homeostatic conditions (Supplementary Fig. 6a and b).

Since pre-IR treatment of NCGC-023 protected human enteroids from radiation-induced death, we evaluated the effects of NCGC-023 administered post-IR. In contrast to pre-IR experiments, post-IR treatment with NCGC-023 administered 1 or 24 h after 4 or 6 Gy X-rays did not significantly rescue organoid viability compared to DMSO controls, although it also did not worsen outcomes (Supplementary Fig. 7a and b). Collectively, these results indicate that transient LATS1/2 inhibition by NCGC-023 protects human enteroids from radiation when administered prior to radiation, while not promoting aberrant growth in unirradiated enteroids in vitro.

### Transient NCGC-023 treatment prior to high-dose irradiation ameliorates GI-ARS in mice

**Fig. 2:**
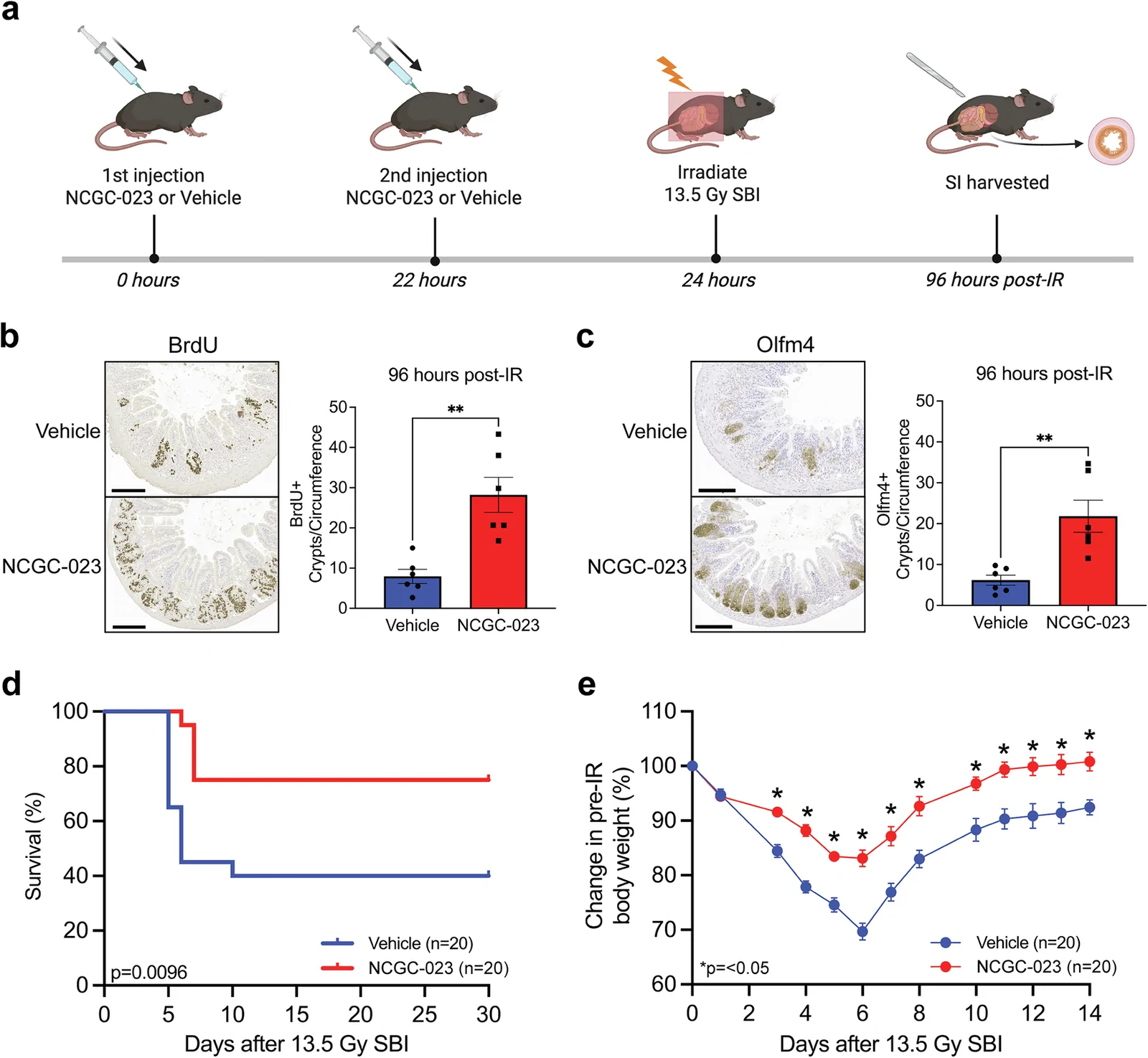
Prophylactic NCGC-023 treatment before irradiation promotes small intestinal regeneration and protects mice from GI-ARS. **a** Experimental schematic. C57BL/6N mice were treated with vehicle or NCGC-023 (50 mg/kg) administered 24 and 2 h prior to irradiation (–24 h, –2 h), exposed to 13.5 Gy sub-total-body irradiation (SBI), and small intestines were harvested 96 h post-irradiation for analysis. Created in BioRender. Driver, L. (2026) https://BioRender.com/nmhvx4o. **b** BrdU immunohistochemistry in small intestinal sections from C57BL/6N mice treated in a. Quantification of BrdU^+^ regenerating crypts per intestinal circumference is shown. Scale bar, 200 µm. Each dot represents one mouse, *n* = 6 per group. Error bars represent SEM; **p < 0.01 via unpaired two-tailed *t* test. **c** Olfm4 immunohistochemistry in small intestinal sections from mice treated in (**a**). Quantification of Olfm4^+^ crypts per intestinal circumference is shown. Scale bar, 200 µm. Each dot represents one mouse, *n* = 6 per group. Error bars represent SEM; **p < 0.01 via unpaired two-tailed *t* test. **d** Kaplan-Meier survival analysis of C57BL/6 N male and female mice treated with –24 h, –2 h vehicle or NCGC-023 (50 mg/kg) and followed for up to 30 days post-13.5 Gy SBI. *n* = 20 mice per group, *p* = 0.0096 by Kaplan-Meier survival analysis (log-rank test). **e** Percent change in body weight relative to pre-irradiation baseline for mice listed in (**d**). *n* = 20 mice per group. Error bars represent SEM. *p < 0.05 by two-way ANOVA with a Bonferroni post hoc test.

Given the significant effect of NCGC-023 in protecting human enteroids from radiation pre-IR in vitro, we pursued a prophylactic treatment paradigm in vivo by administering two doses of NCGC-023 at 50 mg/kg prior to radiation. This drug dose was estimated to reach a plasma concentration of approximately 3 µM in mice based on our pharmacokinetic data. Adult C57BL/6N mice were administered vehicle or NCGC-023 (50 mg/kg) 24 and 2 h (–24 h and –2 h) via IP injection prior to 13.5 Gy sub-total-body irradiation (SBI), a radiation dose known to induce severe GI injury^49^ (Fig. 2a). Lead shielding was applied to the head and forelimbs, providing approximately 20% bone marrow protection to mitigate hematopoietic acute radiation syndrome (H-ARS) while permitting assessment of GI-specific injury^50,51^. Small intestines were harvested 96 h post-IR to evaluate crypt regeneration (Fig. 2a). Prophylactic treatment of NCGC-023 significantly enhanced crypt proliferation following irradiation, as evidenced by an increased number of BrdU^+^ regenerating crypts in the small intestine compared to vehicle controls (Fig. 2b). Consistently, NCGC-023-treated mice exhibited a significant increase in the number of surviving crypts expressing Olfm4, a marker of intestinal stem cells (ISCs)^52^, indicating improved stem cell recovery (Fig. 2c).

To assess whether transient LATS1/2 inhibition using the same prophylactic paradigm confers protection against GI-ARS development, survival was monitored in C57BL/6N mice pre-treated with vehicle or NCGC-023 (50 mg/kg) prior to 13.5 Gy SBI. GI-ARS-associated mortality within this model typically occurs within 10 days post-IR, whereas H-ARS manifests over a longer timeframe (~30 days)^2,53^. Vehicle-treated mice exhibited 60% mortality within 10 days, consistent with GI-ARS, whereas only 20% mortality was observed in the NCGC-023 group (Fig. 2d). Moreover, NCGC-023-treated mice maintained and recovered body weight significantly faster than vehicle controls, in which NCGC-023-treated mice returned to baseline body weight by week two post-IR (Fig. 2e). In contrast, NCGC-023 did not mitigate GI-ARS in mice when it was administered at multiple concentrations and time points after 13.5 Gy SBI (Supplementary Fig. 8a–c). Collectively, our results indicate that NCGC-023 significantly protects mice from GI-ARS and improves intestinal regeneration when administered prophylactically before high-dose radiation exposure.

### NCGC-023 protects crypt cells from radiation-induced DNA damage

**Fig. 3:**
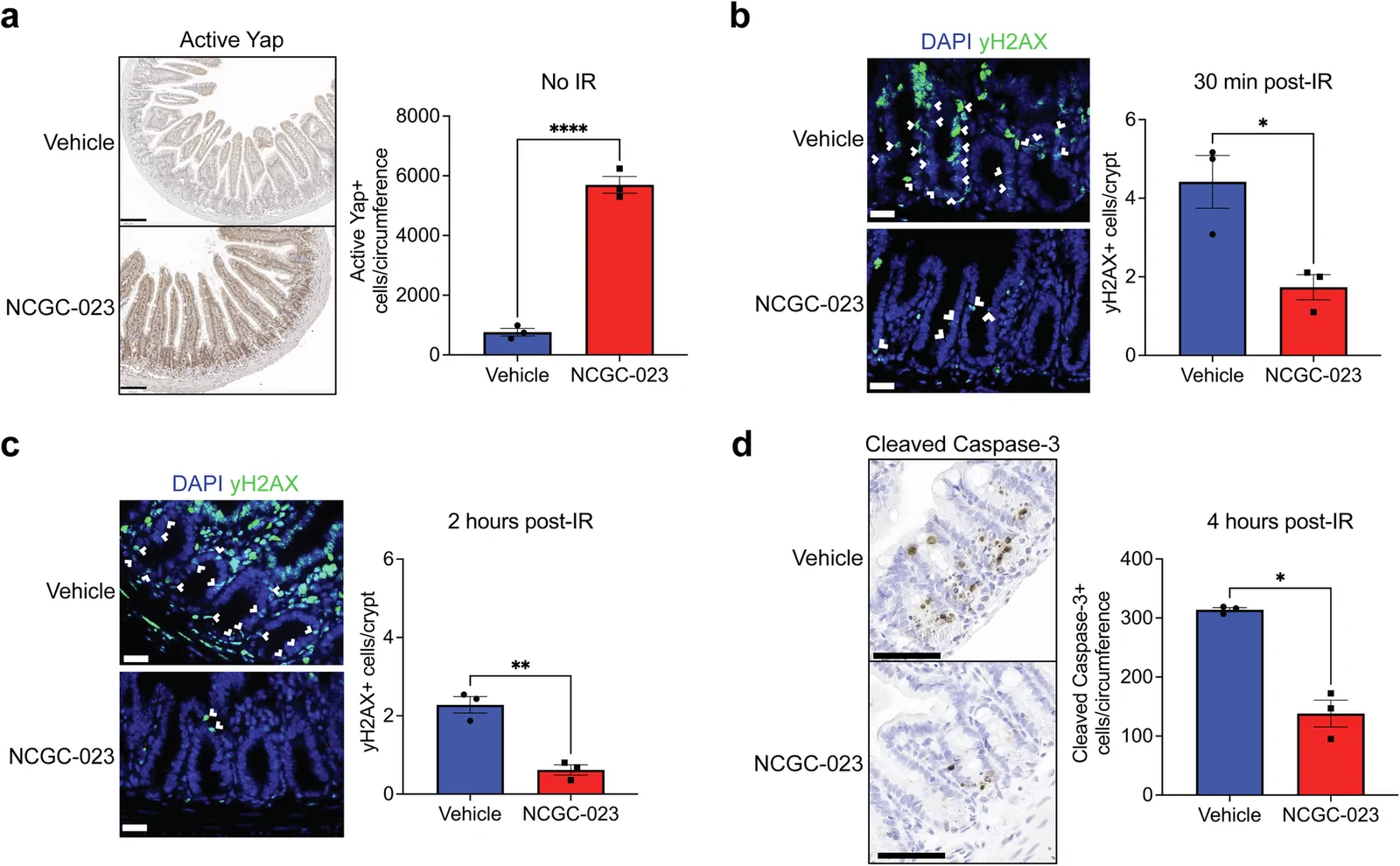
Prophylactic NCGC-023 treatment before irradiation protects intestinal crypt cells from radiation-induced acute DNA damage and apoptosis. **a** Unirradiated C57BL/6N mice treated with vehicle or 50 mg/kg NCGC-023 24 and 2 h (–24 h, –2 h) prior to tissue harvest. The small intestines were stained for active (nuclear) Yap protein. Scale bar 100 µm. ****p < 0.0001 by two-tailed *t* test. **b** C57BL/6N mice treated with vehicle or 50 mg/kg NCGC-023 (–24 h, –2 h) prior to 13.5 Gy SBI. The small intestines were harvested 30 min post-IR to stain for γH2AX (green) and DAPI (blue). Scale bar 25 µm. *p < 0.05 by two-tailed *t* test. **c** C57BL/6N mice treated with vehicle or 50 mg/kg NCGC-023 (–24 h, –2 h) prior to 13.5 Gy SBI. The small intestines were harvested 2 h post-IR to stain for γH2AX (green) and DAPI (blue). Scale bar 25 µm. **p < 0.01 by two-tailed *t* test. **d** C57BL/6N mice treated with vehicle or 50 mg/kg NCGC-023 (–24 h, –2 h) prior to 13.5 Gy SBI. The small intestines were harvested 4 h post-IR to stain for Cleaved Caspase-3 (brown). Scale bar 60 µm. *p < 0.05 by two-tailed *t* test. For all panels, each dot represents a single mouse, *n* = 3 per group. Error bars represent SEM.

To elucidate the underlying mechanisms by which NCGC-023 protects mice from GI-ARS and promotes regeneration, we first examined its effect on Yap activation and the DNA damage response. Using the same pre-IR NCGC-023 or vehicle dosing regimen described in Fig. 2a, mice were harvested at the time of sham-IR and multiple time points following-IR. Pre-treatment of NCGC-023 resulted in an approximate 5-fold increase in the number of crypt cells expressing active Yap compared to vehicle-treated controls in unirradiated mice (Fig. 3a). Of note, NCGC-023-treated mice exhibited a regional reduction in BrdU^+^ crypt cells (Supplementary Fig. 9a), predominantly within the transit-amplifying (TA) zone compared to vehicle controls (Supplementary Fig. 9b).

To evaluate the effect of NCGC-023 treatment on radiation-induced DNA damage, we assessed γH2AX, a marker of DNA double-strand breaks^54^, in the small intestines of mice 30 min and 2 h after 13.5 Gy SBI. NCGC-023-treated mice demonstrated significantly more rapid resolution of γH2AX foci at both 30 min and 2 h post-IR, consistent with enhanced DNA damage repair kinetics (Fig. 3b, c). In addition, we assessed cell death through the intrinsic pathway of apoptosis 4 h post-IR by staining for Cleaved Caspase-3 (CC3). Our results showed that compared to the vehicle control, NCGC-023 treatment significantly reduced the number of CC3^+^ cells per SI circumference, preferentially in the TA region of the crypts (Fig. 3d). Collectively, these findings reveal that transient LATS1/2i enhances Yap activation, promotes efficient DNA damage resolution, and decreases radiation-induced pre-mitotic apoptosis of crypt cells in the small intestinal epithelium.

### NCGC-023 treatment protects crypt cells from radiation-induced mitotic catastrophe

**Fig. 4:**
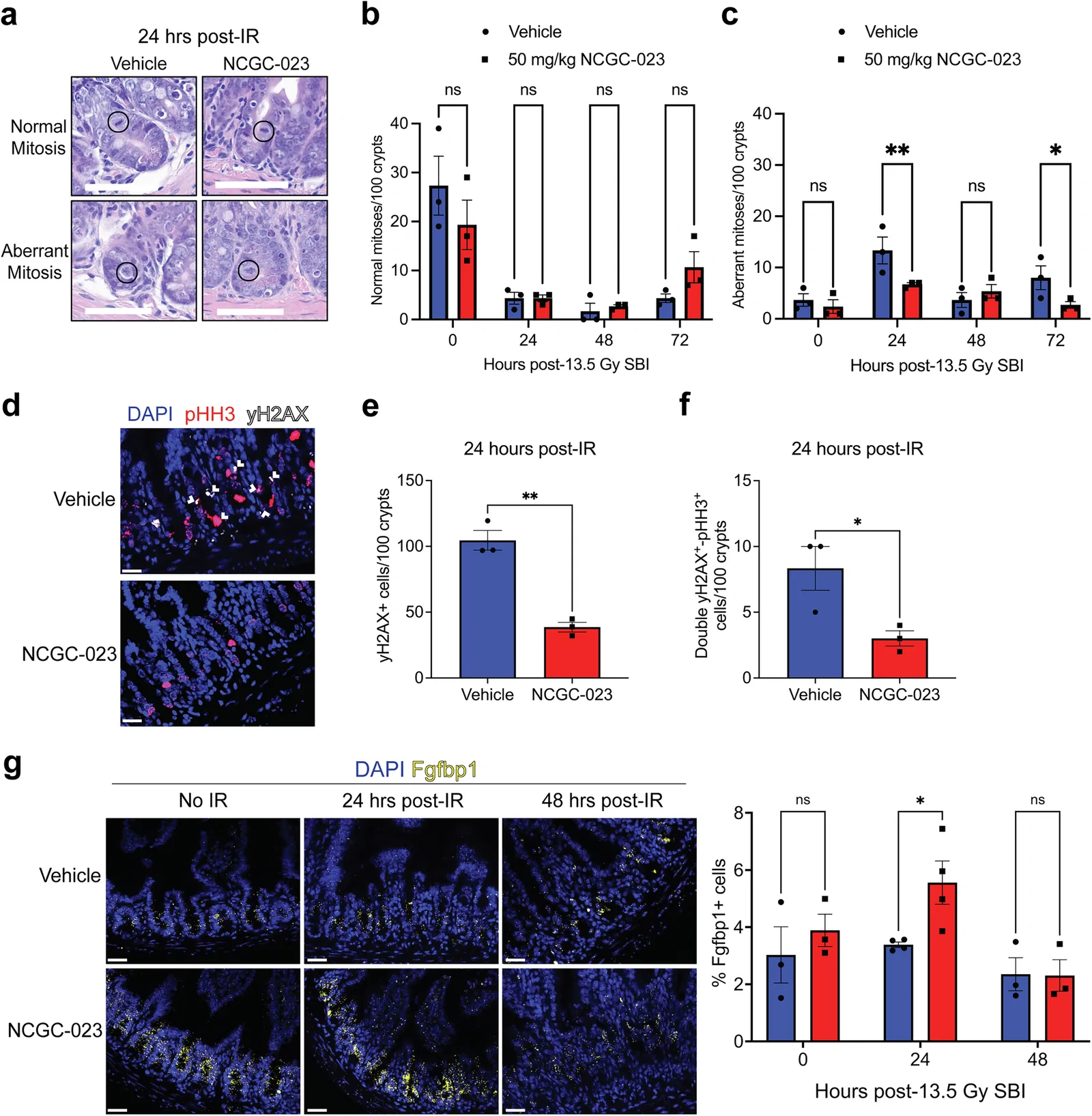
Prophylactic NCGC-023 treatment before irradiation protects proliferating crypt cells from radiation-induced mitotic catastrophe. **a** Normal and aberrant mitoses (circled) in vehicle- or 50 mg/kg NCGC-023-treated mice 24 h post-13.5 Gy SBI. Scale bar 60 µm. **b** Normal mitoses per 100 crypts. No IR vehicle vs. NCGC-023 *p* = 0.0874, 24 h vehicle vs. NCGC-023 *p* = 0.9999, 48 h vehicle vs. NCGC-023 *p* = 0.8228, 72 h vehicle vs. NCGC-023 *p* = 0.1688. *p* values were calculated by two-way ANOVA with a Bonferroni post hoc test. **c** Aberrant mitoses per 100 crypts. No IR vehicle vs. NCGC-023 *p* = 0.5572, 24 h vehicle vs. NCGC-023 **p = 0.0085, 48 h vehicle vs. NCGC-023 *p* = 0.4644, 72 h vehicle vs. NCGC-023 *p = 0.0290. *p* values were calculated by two-way ANOVA with a Bonferroni post hoc test. **d** C57BL/6N mice were treated with vehicle or 50 mg/kg NCGC-023 24 h and 2 h prior to 13.5 Gy SBI. The small intestines were harvested 24 h post-IR to stain for phospho-histone H3 (pHH3) (red), γH2AX (white), and DAPI (blue). White arrows indicate cells stained double-positive for γH2AX and pHH3. Scale bar 25 µm. **e** Quantification of γH2AX-positive cells per 100 crypts from panel (**d**). **p < 0.01 by two-tailed *t* test. **f** Quantification of cells double positive for pHH3 and γH2AX per 100 crypts from panel (**d**). *p < 0.05 by two-tailed *t* test. **g** C57BL/6N mice treated with vehicle or 50 mg/kg NCGC-023 24 h and 2 h prior to 13.5 Gy SBI. The small intestines were harvested at no IR, 24 h, or 48 h post-IR to stain for *Fgfbp1* mRNA via RNAscope (yellow) and counterstained with DAPI (blue). Scale bar 25 µm. The percentage of *Fgfbp1*^*+*^ cells per crypt region, no IR NCGC-023 vs. vehicle *p* = 0.9292; 24 h post-IR NCGC-023 vs. vehicle *p* = 0.0452; 48 h post-IR NCGC-023 vs. vehicle *p* = 0.9999. *p* values were calculated by one-way ANOVA, followed by Tukey’s post hoc test for pairwise comparisons. For all panels, each dot represents a single mouse. All error bars represent SEM.

Multiple studies have demonstrated that the division of intestinal crypt cells with unrepaired DNA damage can lead to aberrant mitosis or mitotic catastrophe, which is associated with the development of GI-ARS after high-dose irradiation^3,50,55,56^. To determine the effect of NCGC-023 on mitosis, we quantified normal and aberrant mitotic figures in intestinal crypt cells treated with 50 mg/kg NCGC-023 (–24 h, –2 h) or vehicle from 0 to 72 h after 13.5 Gy SBI (Fig. 4a). No significant difference was observed in the number of crypt cells undergoing normal mitoses between NCGC-023- and vehicle-treated mice (Fig. 4b). In contrast, the number of crypts exhibiting aberrant mitoses—categorized by chromosomal misalignment or micronuclei—was significantly reduced in NCGC-023-treated mice at 24 h and 72 h post-irradiation compared to vehicle-treated controls (Fig. 4c). Consistent with improved DNA damage resolution seen in Fig. 3b, c, NCGC-023 treatment significantly decreased the number of γH2AX^+^ crypt cells at 24 h post-IR relative to vehicle-treated mice (Fig. 4d, e). Furthermore, double immunostaining for γH2AX and phospho-histone H3 (pHH3) revealed a significant reduction in mitotic crypt cells harboring unrepaired DNA damage (γH2AX^+^/pHH3^+^ double-positive cells) in NCGC-023-treated mice 24 h post-IR (Fig. 4d, f**)**.

Given that most vehicle-treated mice exhibited mitotic catastrophe in the TA region, we examined the effect of NCGC-023 treatment on crypt cells expressing *Fgfbp1*, a marker enriched in TA cells and the cycling CBCs^57–59^. The percentage of *Fgfbp1*^*+*^ cells per crypt region was significantly higher in NCGC-023-treated mice 24 h post-IR (Fig. 4g). However, no significant difference in *Fgfbp1* mRNA was observed between NCGC-023- and vehicle-treated mice at baseline (no IR) and 48 h post-IR (Fig. 4g). Together, these findings demonstrate that prophylactic LATS1/2i via NCGC-023 suppresses radiation-induced aberrant mitosis and mitotic catastrophe of rapidly cycling small intestinal crypt cells.

### NCGC-023 treatment prior to irradiation promotes fetal-like reprogramming of crypt cells

We and others have demonstrated that a key mechanism by which the intestinal epithelium regenerates in response to severe radiation injury is fetal-like transcriptional reprogramming of surviving epithelial cells^28–30,60^. Reprogramming of irradiated intestinal epithelial cells produces revival stem cells (revSCs), which express a marker gene *Clusterin* (*Clu*), to generate new Lgr5^+^ ISCs and repopulate the damaged epithelium^28^. The emergence of revSCs starts around 2 days after radiation exposure and peaks around 2.5 to 3 days post-IR, a process that is preceded by activation of Yap and the tumor suppressor p53^28–30,60^.

**Fig. 5:**
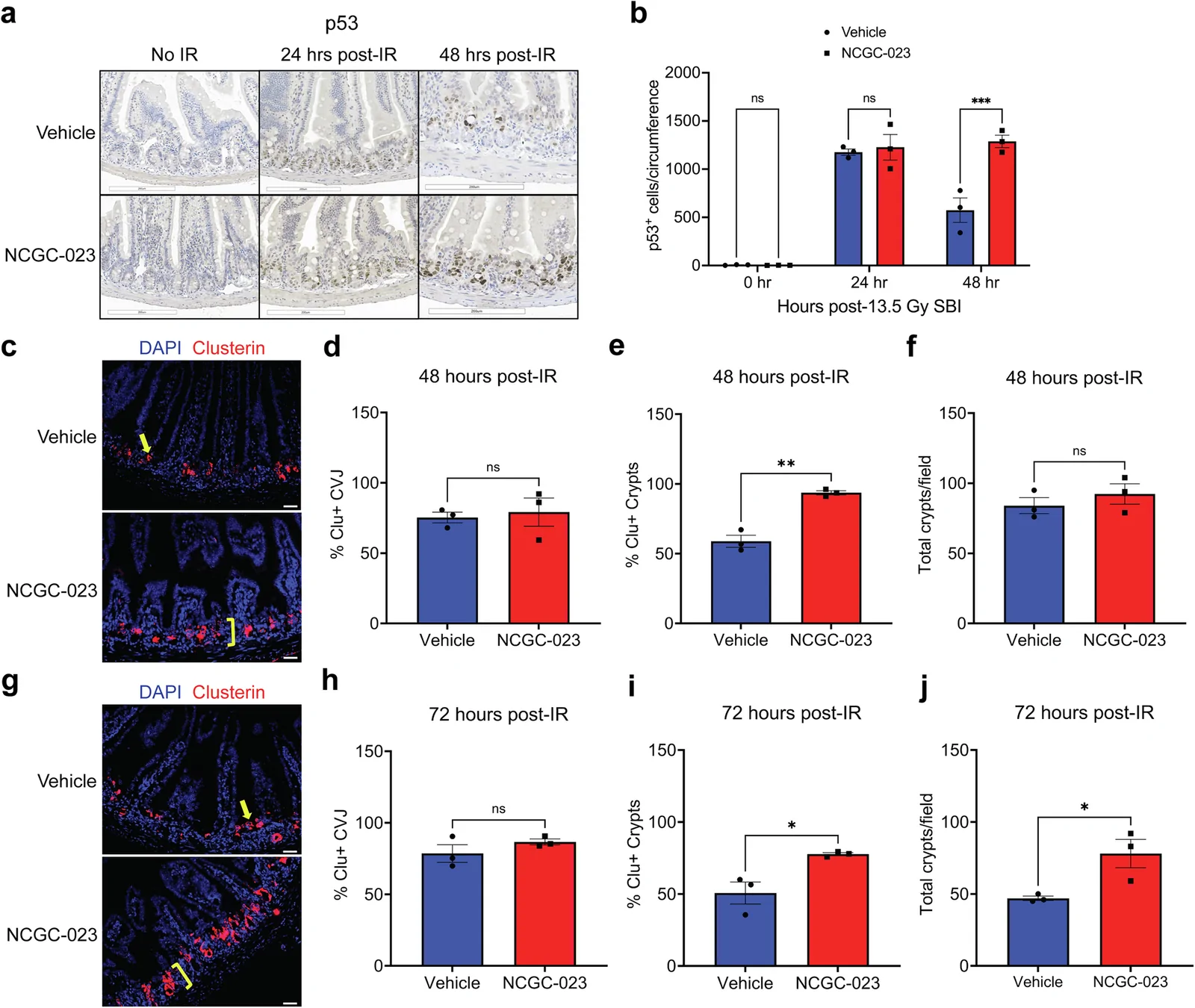
Prophylactic NCGC-023 treatment before irradiation promotes regenerative reprogramming of the irradiated small intestinal epithelium. **a** C57BL/6N mice were treated with vehicle or 50 mg/kg NCGC-023 24 and 2 h (–24 h, –2 h) prior to sham-IR or 13.5 Gy SBI. The small intestines were harvested 24 h or 48 h post-IR to stain for p53 protein (brown). Scale bar 200 µm. **b** Average number of p53^+^ cells per circumference from panel (**a**). No IR vehicle vs. NCGC-023 *p* = 0.9999, 24 h vehicle vs. NCGC-023 *p* = 0.9627, 48 h vehicle vs. NCGC-023 ***p = 0.0001. **c** C57BL/6N mice were treated with vehicle or 50 mg/kg NCGC-023 (–24 h, –2 h) prior to 13.5 Gy SBI. The small intestines were harvested 48 h post-IR to stain for *Clu* mRNA via RNAScope (red) and DAPI (blue). Scale bar 100 µm. **d** The percentage of Clu^+^ crypt-villus-junction (CVJ) 48 h post-IR, *p* = 0.7376. **e** The percentage of Clu^+^ crypts 48 h post-IR, **p = 0.0016. **f** Total number of crypts per ×10 field at 48 h post-IR, *p* = 0.4175. **g** C57BL/6N mice were treated with vehicle or 50 mg/kg NCGC-023 (–24 h, –2 h) prior to 13.5 Gy SBI. The small intestines were harvested 72 h post-IR to stain for *Clu* mRNA via RNAScope (red) and DAPI (blue). Scale bar 100 µm. **h** The percentage of Clu^+^ CVJ 72 h post-IR, *p* = 0.3761. **i** The percentage of Clu^+^ crypts 72 h post-IR, *p = 0.0142. **j** Total number of crypts per ×10 field 72 h post-IR, *p = 0.0109. For all panels, each dot represents a single mouse. Error bars represent SEM. For panel **b**, *p* values were calculated via two-way ANOVA followed by Bonferroni post hoc correction. For panels **d**–**f** and **h**–**j**, via a two-tailed *t* test.

To assess the effect of NCGC-023 treatment before IR on the emergence and maturation of revSCs, we first examined intestinal epithelial cells that expressed p53 protein 1 and 2 days after 13.5 Gy SBI. Histological assessment revealed that the number of crypt cells expressing p53 protein was significantly higher in NCGC-023-treated mice 48 h after 13.5 Gy SBI when compared to vehicle controls, consistent with the pre-emergence of Clu^+^ RevSCs^29^ (Fig. 5a, b). *Clu* mRNA was expressed in intestinal epithelial cells of both vehicle- and NCGC-023-treated mice 48 and 72 h after 13.5 Gy SBI; however, the microanatomic distribution of these cells varied between treatments. Vehicle-treated mice showed epithelial cells expressing *Clu* mRNA only at the crypt-villus junction (CVJ), while NCGC-023-treated mice exhibited a significantly higher number of epithelial cells expressing *Clu* mRNA extending from the CVJ to the crypt base (Fig. 5c–j). Furthermore, the number of total crypts in the small intestines was significantly higher in NCGC-023-treated irradiated mice compared to irradiated mice treated with vehicle 72 h post-IR (Fig. 5j). Together, these results indicate that transient inhibition of LATS1/2 by NCGC-023 prior to high-dose irradiation promotes revSC-mediated regenerative programming of intestinal crypt cells in vivo.

### NCGC-023 induces Yap-dependent and -independent pathways downstream of LATS1/2

**Fig. 6:**
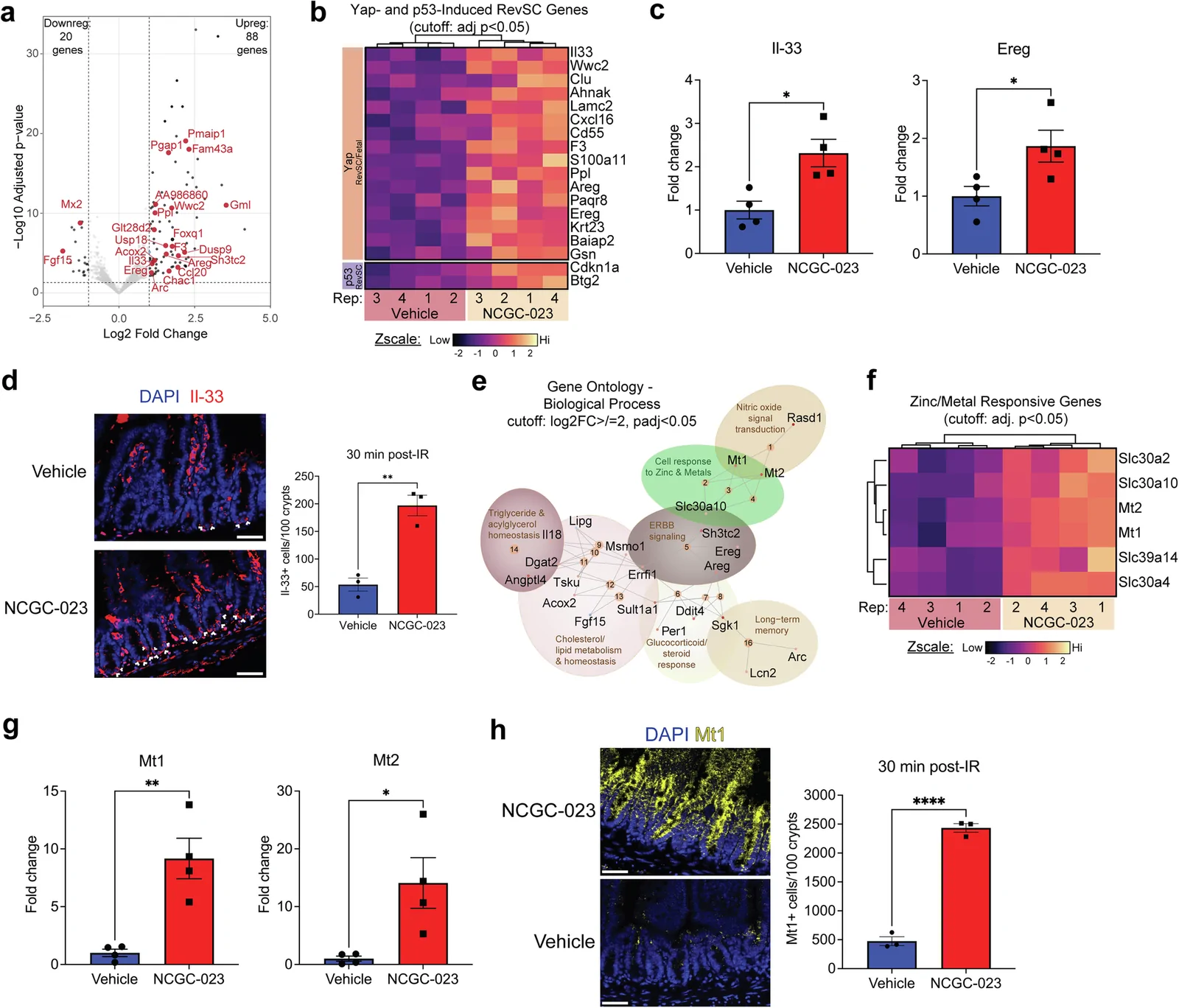
RNA sequencing reveals NCGC-023 induces Yap-mediated fetal-like reprogramming and metallothionein-mediated heavy metal response. **a** 108 differentially expressed genes were identified in bulk RNA-seq comparing small intestinal crypts harvested from unirradiated C57BL/6N mice treated with vehicle or 50 mg/kg NCGC-023 at –24 h and –2 h before tissue harvest. Numerous known Yap-regulated genes (from Gregorieff et al.) were identified in the differentially expressed genes, labeled and colored in red. **b** NCGC-023 treatment of mouse crypts induces genes typically upregulated in RevSCs, as evident in a Zscaled heatmap of RNA-seq data displaying all RevSC-related genes with differential expression in the RNA-seq data (*p* < 0.05). **c** qRT-qPCR validating Yap-dependent genes upregulated by NCGC-023 treatment. *Il-33*, *p = 0.0132, *Ereg*, *p = 0.0365. **d** C57BL/6 N mice were treated with vehicle or 50 mg/kg NCGC-023 (–24 h, –2 h) prior to 13.5 Gy SBI. The small intestines were harvested 30 min post-IR to stain for Il-33 protein (red) and DAPI (blue). White arrows denote Il-33^+^ crypt cells. Scale bar 50 μm. Quantification of Il-33^+^ cells per 100 crypts, **p = 0.0029. **e** Overrepresentation analysis reveals several “Gene Ontology – Biological Process” pathways with significant enrichment in the list of RNA-seq differentially-expressed genes (fold change >/=2 and *p* < 0.05). **f** Hierarchical unsupervised sample clustering and heatmap visualization of significantly altered genes reveals that NCGC-023-induced expression of genes contained in metal- and zinc-related Gene-Ontology pathways. **g** qRT-PCR validating metallothionein genes upregulated by NCGC-023 treatment. *Mt1*, **p = 0.0038, *Mt2*, *p = 0.0250. **h** C57BL/6N mice were treated with vehicle or 50 mg/kg NCGC-023 (–24 h, –2 h) prior to 13.5 Gy SBI. The small intestines were harvested 30 min post-IR to stain for Mt1 mRNA via RNAscope (yellow) and DAPI (blue). Scale bar 50 μm. Quantification of *Mt1*^*+*^ cells per 100 crypts, ****p < 0.001. For panels **c**, **d**, **g**, and **h**, each dot represents a single mouse. Error bars represent SEM. *P* values were calculated by two-tailed *t* test.

To elucidate the impact of NCGC-023 treatment on gene transcription in vivo, we collected intestinal crypts from unirradiated mice treated with NCGC-023 (50 mg/kg, –24, –2 h before tissue harvest) for bulk RNA sequencing (RNA-seq). Results from the RNA-seq experiment identified 108 differentially expressed genes significantly affected by NCGC-023 treatment in unirradiated crypts (adjusted *p*-value less than 0.05 and fold change of 2 or more) (Fig. 6a). 88 of these genes were upregulated, and 20 were downregulated by NCGC-023. Numerous of these differentially regulated genes are known to be regulated by Yap^23^ and/or induced during fetal-like reprogramming of intestinal epithelial cells^24,28,29,61^ (Supplementary Fig. 10). In addition, we observed significant upregulation of revSC marker genes^28,29^ in NCGC-023-treated crypts, including *Il33*, *Clu*, *F3*, *Areg* and *Ereg* (transcriptional targets of Yap) as well as *Cdkn1a* and *Btg2* (transcriptional targets of p53) (Fig. 6b). qRT-PCR validation of the RNA-seq findings confirmed that NCGC-023 significantly upregulated Yap-dependent genes such as *Il-33* and *Ereg* (Fig. 6c). Moreover, IF staining revealed that NCGC-023 significantly increased the number of crypt cells expressing Il-33 protein 30 min post-13.5 Gy SBI (Fig. 6d). Notably, NCGC-023 treatment resulted in significant downregulation of CBC markers and Wnt target genes, *Ascl2* and *Lgr5*, a phenomenon that is consistent with Yap-mediated reprogramming^23,62,63^ (Supplementary Fig. 11a and b). The changes in CBCs were validated by a significant reduction in Olfm4^+^ epithelial cells in unirradiated mice treated with NCGC-023 (–24 h, –2 h) (Supplementary Fig. 11c). Collectively, these results suggest that NCGC-023 treatment significantly induces transcriptional reprogramming of intestinal crypt cells in mice in the absence of radiation injury.

In addition to the alterations in genes involved in Yap-mediated transcriptional reprogramming, overrepresentation analysis revealed significant enrichment of several “Gene Ontology – Biological Process” pathways in NCGC-023-treated crypts (Fig. 6e and Supplementary Fig. 12). Of note, we observed a significant enrichment of genes involved in the cellular response to zinc and metals, which is a pathway known to be regulated by LATS1/2 independent of Yap^64^. A closer examination of this pathway revealed significant upregulation of multiple effector genes, including metallothionein family genes *Mt1* and *Mt2* (Fig. 6f). qRT-PCR indicated NCGC-023 significantly induced Yap-independent metallothionein genes, *Mt1* and *Mt2*, consistent with RNA-seq results (Fig. 6g). Metallothionein induction was further validated through IF RNAscope staining, which demonstrated a significant increase in *Mt1* mRNA 30 min post-13.5 Gy SBI (Fig. 6h).

To further evaluate whether NCGC-023 modulates additional hallmark signaling cascades, we performed overrepresentation analysis across curated gene sets spanning EGFR/MAPK, NOTCH1, WNT, TGFβ, FOXO3, and p53 pathways (Supplementary Fig. 13). Neither EGFR signaling nor MAPK transcriptional targets reached statistical significance, nor did NOTCH1, WNT/TCF-dependent, or TGFβ gene sets, indicating that NCGC-023 does not broadly activate or suppress these cascades at the transcriptional level. Among p53-related gene sets, direct p53 targets (Fischer meta-analysis) achieved significance after multiple testing correction (18/261 genes; adj. *p* = 0.029), consistent with the upregulation of canonical p53 effectors *Cdkn1a* and *Btg2* noted above.

Together, these findings indicate that the dominant transcriptional response to NCGC-023 is channeled through Yap-dependent intestinal stem cell reprogramming, a Yap-independent stress response (Metallothionein), and p53 pathway engagement, without broad activation of other major canonical signaling cascades.

### Transient NCGC-023 treatment does not exacerbate radiation-induced delayed injury

**Fig. 7:**
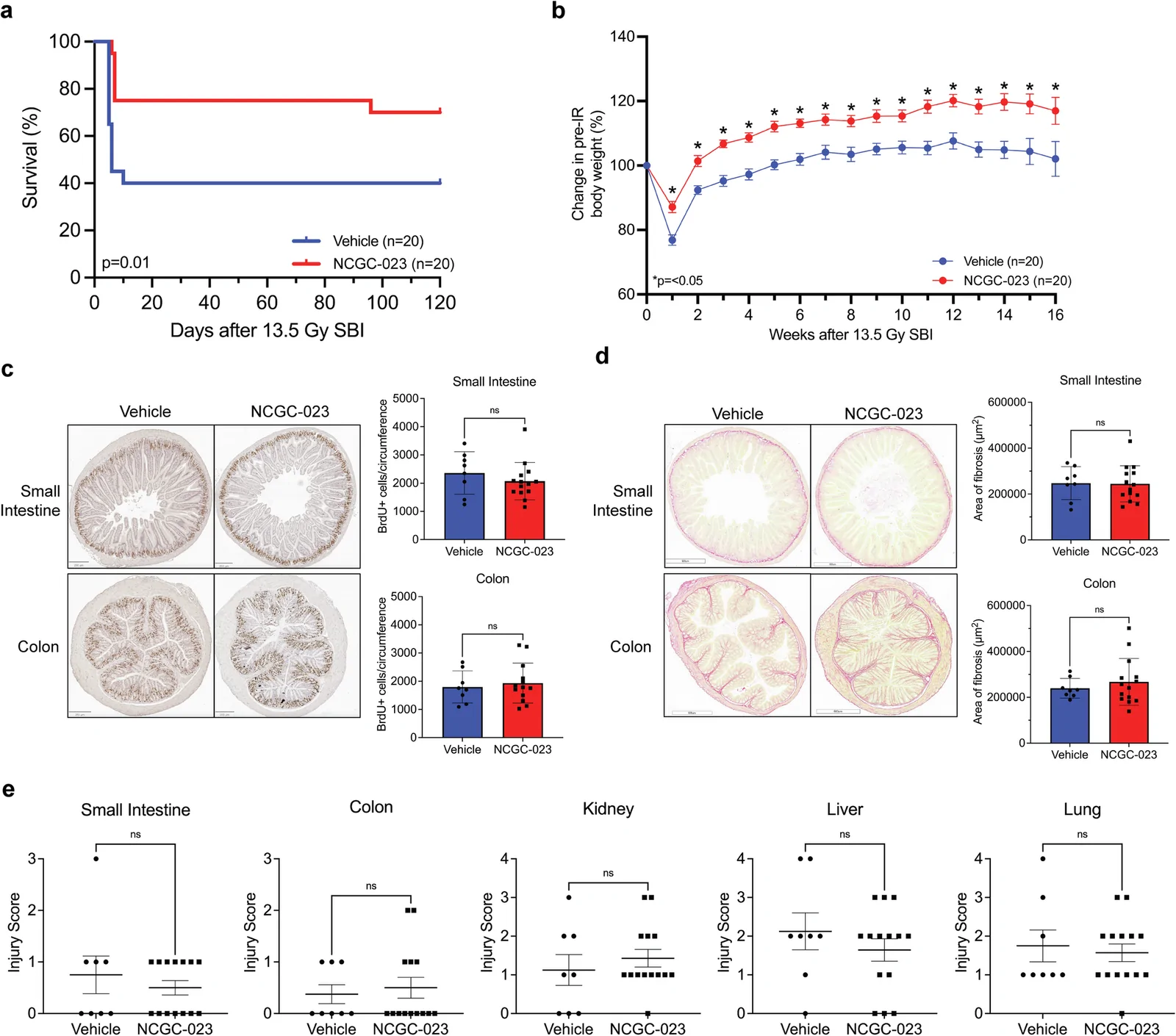
Prophylactic NCGC-023 treatment before irradiation does not exacerbate radiation-induced delayed injury. **a** C57BL/6N mice were treated with vehicle or 50 mg/kg NCGC-023 (–24 h, –2 h) prior to 13.5 Gy SBI. The survival of mice was followed up to 120 days after IR. *n* = 20 mice/group, *p* = 0.01 by Kaplan-Meier survival analysis (log-rank test). **b** Percent change in pre-IR bodyweight in C57BL/6 N male and female mice in panel (**a**). *n* = 20 mice/group. Error bars represent SEM. *p < 0.05 by two-way ANOVA with a Bonferroni post hoc test. **c** BrdU^+^ cells per small intestine or colon circumference in vehicle or 50 mg/kg NCGC-023 treated mice harvested 120 days post-13.5 Gy SBI. Scale bar 250 µm. Each point represents one mouse, *n* = 8 mice/vehicle, *n* = 13 mice/NCGC-023; ns: *p* > 0.05. **d** Area of fibrosis (µm^2^) examined by Picrosirius red staining per small intestine or colon circumference in vehicle or 50 mg/kg NCGC-023 treated mice harvested 120 days post-13.5 Gy SBI. Scale bar 600 µm. Each point represents one mouse, *n* = 8 mice/vehicle, *n* = 13 mice/NCGC-023; ns: *p* > 0.05. **e** Injury score per organ in vehicle or 50 mg/kg NCGC-023 treated mice harvested 120 days post-13.5 Gy SBI. Each point represents one mouse, *n* = 8 mice/vehicle, *n* = 13 mice/NCGC-023. ns: *p* > 0.05. Error bars represent SEM. *p* values were calculated by two-tailed *t* test.

Surviving NCGC-023- or vehicle-treated mice (from Figure 2d) were monitored for up to 4 months post-IR. NCGC-023-treated mice demonstrated more rapid and sustained body weight recovery, exceeding 100%, and exhibited 70% long-term survival compared to 40% survival in vehicle-treated mice. Vehicle-treated mice recovered to baseline bodyweight by week 5 and plateaued thereafter (Fig. 7a, b). To assess the impact of transient NCGC-023 on late radiation effects, primary organs within the radiation field—including the small intestine, colon, kidneys, liver, and lungs—were collected from mice that survived 4 months after 13.5 Gy SBI for histological analysis. Examination of the small intestine and colon revealed no significant increase in proliferation indicated by BrdU^+^ epithelial cells in NCGC-023-treated mice compared to vehicle controls 4 months after irradiation (Fig. 7c). Additionally, Sirius Red staining indicated that NCGC-023 did not promote fibrosis through connective tissue deposition in either organ when compared to vehicle controls (Fig. 7d). The injury of key organs within the field of irradiation was also examined through semi-quantitative scoring (Supplementary Fig. 14a and b). The results of semi-quantitative scoring of tissue injury revealed that NCGC-023 treatment did not exacerbate delayed injury in any of the tissues harvested 4 months post-IR (Fig. 7e). Furthermore, no neoplastic or dysplastic lesions were observed in any irradiated animals treated with vehicle or NCGC-023. Our results from histological examinations collectively suggest that transient NCGC-023 treatment pre-IR provides long-term GI recovery without aggravating delayed organ injury post-IR.

### NCGC-023 does not radioprotect colonic organoids harboring loss of tumor suppressors

Inactivation or sustained disruption of normal physiological Hippo pathway signaling can result in unchecked Yap or Taz activity, promoting oncogenic processes including uncontrolled cellular proliferation, suppression of apoptosis, and induction of epithelial-mesenchymal transition, thereby enhancing malignant potential^65–67^. Specifically, aberrant Hippo signaling that leads to hyperactivation of Yap and/or Taz has been reported to drive colorectal cancer initiation and progression, including premalignant lesions^12,67–70^. Therefore, we sought to examine the effect of transient NCGC-023 treatment on the growth and radiosensitivity of mouse colonic organoids that harbor a deletion in the tumor suppressors *Apc* or *Trp53* in vitro.

**Fig. 8:**
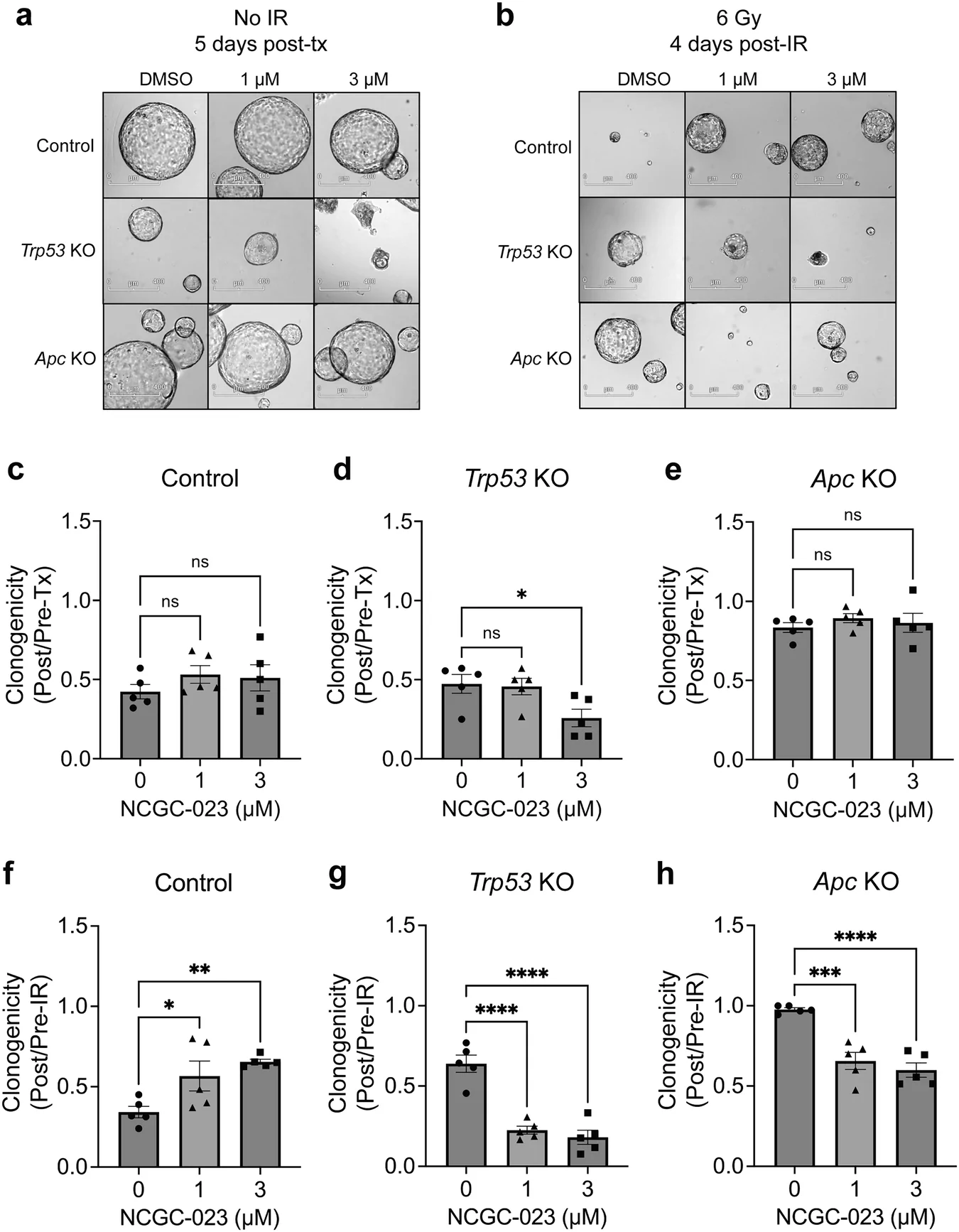
NCGC-023 does not protect mouse colonic organoids harboring *Trp53* or *Apc* loss from radiation in vitro. **a** Representative images of unirradiated mouse colonic organoids treated with DMSO, 1 µM, or 3 µM NCGC-023 for 24 h and followed for 5 days post-NCGC-023 treatment. Scale bar 400 µm. **b** Representative images of mouse colonic organoids treated with DMSO, 1 µM, or 3 µM NCGC-023 for 24 h prior to irradiation with 6 Gy and followed for 5 days post-NCGC-023 treatment (4 days post-6 Gy). Scale bar 400 µm. **c** Clonogenicity of unirradiated control mouse colonic organoids 5 days post-treatment. DMSO vs. 1 µM NCGC-023 *p* = 0.4663, DMSO vs. 3 µM NCGC-023 *p* = 0.6052. **d** Clonogenicity of unirradiated *Trp53* KO mouse colonic organoids 5 days post-treatment. DMSO vs. 1 µM NCGC-023 *p* = 0.9761, DMSO vs. 3 µM NCGC-023 *p = 0.0437. **e** Clonogenicity of unirradiated *Apc* KO mouse colonic organoids 5 days post-treatment. DMSO vs. 1 µM NCGC-023 *p* = 0.5936, DMSO vs. 3 µM NCGC-023 *p* = 0.8736. **f** Clonogenicity of control mouse colonic organoids 5 days post-treatment (4 days post-6 Gy). DMSO vs. 1 µM NCGC-023 *p = 0.0448, DMSO vs. 3 µM NCGC-023 **p = 0.0065. **g** Clonogenicity of *Trp53* KO mouse colonic organoids 5 days post-treatment (4 days post-6 Gy). DMSO vs. 1 µM NCGC-023 ****p < 0.0001, DMSO vs. 3 µM NCGC-023 ****p < 0.0001. **h** Clonogenicity of *Apc* KO mouse colonic organoids 5 days post-treatment (4 days post-6 Gy). DMSO vs. 1 µM NCGC-023 ***p = 0.0003, DMSO vs. 3 µM NCGC-023 ****p = <0.0001. For panels **c**–**h**, each point represents one well, *n* = 5. Differences between groups were analyzed using a one-way ANOVA, followed by Tukey’s post hoc test for pairwise comparisons. Error bars represent SEM.

CRISPR/Cas9-mediated genome editing was used to generate knockout (KO) colonic organoid lines targeting *Apc* (95% KO) or *Trp53* (98% KO), to compare to a control line bearing a guide targeting a non-coding safe locus^71^ (Supplementary Fig. 15a and b). Colonic organoids were treated with DMSO, 1 µM, or 3 µM NCGC-023 for 24 h prior to sham-IR or 6 Gy IR and subsequently followed for 4 days (Fig. 8a and b). NCGC-023 did not promote the growth of control, *Trp53* KO or *Apc* KO organoids in unirradiated conditions; in fact, NCGC-023 decreased clonogenicity at 3 µM in *Trp53* KO organoids (Fig. 8c–e). In irradiated organoids treated with NCGC-023 24 h prior to 6 Gy, transient NCGC-023 treatment significantly increased the clonogenicity of control colonic organoids 4 days post-IR (Fig. 8f). In contrast, transient treatment of *Trp53* KO or *Apc* KO colonic organoids with NCGC-023 before 6 Gy caused a significant decrease in clonogenicity of both lines (Fig. 8f–h). This is consistent with previous findings suggesting LATS1/2i suppresses the metastatic potential of colorectal cancers^62^. Thus, these results indicate that transient NCGC-023 treatment does not significantly promote the growth of *Trp53* KO or *Apc* KO colonic organoids in the absence of radiation, nor protects these premalignant organoids from radiation in vitro.

## Discussion

Here, we have characterized a novel small molecule kinase inhibitor, NCGC-023, that is highly potent and selective to inhibit LATS1 and LATS2. NCGC-023 effectively induces nuclear translocation of Yap protein in vitro and in vivo. Transient inhibition of LATS1/2 by NCGC-023 before irradiation significantly improves the clonogenicity of human intestinal enteroids in vitro and promotes the regeneration of the intestinal epithelium of C57BL/6N mice in vivo. NCGC-023-treated mice exhibit a significant decrease in radiation-induced DNA damage, pre-mitotic apoptosis, and mitotic catastrophe of irradiated crypt cells. Protection of crypt cells from radiation-induced DNA damage and death results in an acceleration of transcriptional reprogramming, as shown by a significant increase in the number of p53^+^ early revSCs and Clu^+^ mature revSCs in the irradiated crypts. Furthermore, transient NCGC-023 treatment before irradiation ameliorates GI-ARS, and it does not significantly exacerbate any aberrant proliferative, fibrotic, or neoplastic events in treated mice 4 months post-irradiation.

The findings from RNA-seq and histological examinations suggest multiple potential mechanisms by which transient NCGC-023 treatment before irradiation ameliorates GI-ARS. First, NCGC-023 treatment induces reprogramming of crypt cells to a revSC-like state, which is associated with transient cell cycle arrest^28,29^. The decrease in cell proliferation is demonstrated by a significant reduction in BrdU^+^ cells in the upper crypt region of NCGC-023-treated mice. It has been shown that induction of transient cell cycle arrest before irradiation protects mice from GI-ARS and promotes DNA repair of intestinal epithelial cells^51,72^, a phenomenon that is consistent with our results showing a significant decrease in γH2AX^+^ crypt cells 30 min and 2 h after 13.5 Gy SBI in NCGC-treated mice. These results are further supported by a significantly higher percentage of *Fgfbp1*^*+*^ intestinal crypt cells, which are enriched for TA cells and the cycling CBCs, in NCGC-023-treated mice compared with vehicle-treated mice 24 h post-13.5 Gy SBI, indicating that NCGC-023 ameliorates radiation-induced cell death of rapidly proliferating crypt cells. Moreover, NCGC-023 induces transcriptional targets of Yap that can promote crypt regeneration. For example, our results show that the number of crypt cells that express Il-33, a direct target of Yap, is significantly higher in NCGC-023-treated mice. In support of this, it has been shown that Il-33 secreted by crypt cells functions through Paneth cells to promote the regeneration of irradiated intestinal epithelium^73^. Furthermore, NCGC-023 treatment significantly upregulates metallothionein family genes, *Mt1* and *Mt2*, which are downstream of metal-regulatory transcription factor 1 (Mtf1) that is activated upon LATS1/2 inhibition^64^. Metallothionein proteins are well-established regulators of redox homeostasis, enabling cells to mitigate oxidative stress and maintain DNA fidelity^74,75^. Indeed, it has been shown that administration of Mt2 protein before irradiation ameliorates acute intestinal toxicity in mice^76^. Collectively, these findings reveal that transient NCGC-023 treatment before irradiation promotes intestinal regeneration through complementary mechanisms that involve Yap-dependent reprogramming of crypt cells and Yap-independent activation of metallothionein-mediated stress responses, both of which are regulated by LATS1/2.

The known role of LATS1/2 deletion and Yap activation in promoting fibrosis^77^ and aberrant growth of certain organs^20^ has led us to evaluate the impact of transient NCGC-023 treatment on the delayed effects of radiation exposure. Our results from mice 4 months post-IR indicate that transient NCGC-023 treatment before irradiation does not significantly exacerbate delayed injury or aberrant cell proliferation in the small intestine, colon, kidney, liver, or lung. In addition, transient NCGC-023 treatment does not increase the clonogenicity of colonic organoids that lack the tumor suppressor *Apc* or *Trp53* with and without irradiation. These data suggest that transient Yap activation promotes regenerative responses of normal colonic organoids without overtly accelerating the growth of pre-malignant colonic organoids in vitro. However, exposure to ionizing radiation may pose a lifelong risk of developing cancer and multiorgan injury^78^. Therefore, comprehensive studies that follow the development of late effects in irradiated animals treated with vehicle versus NCGC-023 throughout the lifespan will be necessary to determine the long-term consequences of transient LATS1/2 inhibition on tissue health.

There is an unmet need for developing pre-exposure prophylactic treatments that protect individuals who may be anticipatorily exposed to high-dose radiation environments, including first responders and military personnel. In support of this approach, numerous studies have highlighted the importance of prophylactic medical countermeasures for acute radiation syndromes (ARS) in preserving tissue integrity and improving survival following exposure^79–83^. Our results demonstrate a significant effect of NCGC-023 on protecting mice from GI-ARS when given 24 h and 2 h before radiation exposure. This time point of drug administration is comparable to other published studies that develop prophylactic countermeasures for ARS in research animals^80,81,84,85^. While our study utilizes a mouse model of SBI that shields the bone marrow from radiation to prevent death from the hematopoietic (H) acute radiation syndrome (H-ARS) to evaluate GI-ARS, in the scenario of total-body irradiation (TBI), high-dose TBI (>10 Gy) can lead to severe injury in both the hematopoietic system and the GI tract, resulting in a mixed H/GI syndrome^49^. However, in the event of a real-world scenario, most individuals would experience a heterogeneous dose of radiation rather than TBI, similar to a SBI shielding model^5^. Nonetheless, it warrants further investigation to determine the effect of NCGC-023 treatment before high-dose TBI on the recovery of the hematopoietic system and the GI tract. In addition, it will be important to test the combination of NCGC-023 with promising prophylactic countermeasures for H-ARS, such as BIO300^80,81^, on the development of ARS and delayed injury in animal models. Furthermore, it is well established that intestinal organoids in vitro, while recapitulating many features of crypt biology, exhibit transcriptional profiles that diverge from in vivo counterparts in important ways^86–91^. Therefore, a direct transcriptomic comparison between irradiated organoids and irradiated mouse crypts would be a valuable future direction to validate the translational relevance of our in vitro observations and to identify signaling pathways that may be differentially regulated in the two systems.

In summary, our findings demonstrate that transient treatment of mice with our novel small molecule LATS1/2 inhibitor, NCGC-023, before high-dose radiation exposure enhances the regeneration of the intestinal epithelium through Yap-dependent and Yap-independent mechanisms. NCGC-023 also improves overall survival and body weight recovery up to 4 months after irradiation without exacerbating delayed tissue injury. These findings suggest that administration of LATS1/2 inhibitors before radiation exposure is a promising approach to ameliorate severe radiation damage to the small intestines.

## Methods

### Characterizations of NCGC00846023 (referred to as NCGC-023)

NCGC00846023 was listed as compound #28 in the patent application on LATS inhibitors (Patent No. WO2023239727)^40^. Chemical synthesis was accomplished in an analogous fashion to that of Morris et al.^29,92^. In brief, 1-bromo-4-chloro-2-methylbenzene (7.3 mmol, 1.0 eq) was cooled to –78 °C in tetrahydrofuran (10 mL) under a nitrogen atmosphere. n*-B*utyllithium in hexanes (2.5 M, 1.1 eq) was added. The mixture was stirred for 5 min, then a solution of *tert*-butyl (3 R,5S)-3,5-dimethyl-4-oxopiperidine-1-carboxylate (7.3 mmol, 1.0 eq.) in tetrahydrofuran (10 ml) was added. The reaction was allowed to stir for 2 h and slowly raised to 0 °C. It was then poured into an aqueous saturated solution of sodium bicarbonate, extracted with ethyl acetate, and purified via silica gel chromatography to give compound 1 in 62% yield. m/s ES+ [M-OC(CH_3_)_3_] + : 280.0. Compound 1 (Supplementary Fig. 1) (2.1 mmol) was dissolved in dichloromethane (10 mL) and treated with trifluoroacetic acid (3.0 mL) at room temperature for 1 h. The solvent and trifluoroacetic acid were removed by rotary evaporation. The residue was treated with aqueous sodium bicarbonate and extracted with ethyl acetate. The organic phase was taken and solvent removed by rotary evaporation to give compound 2 in 95% yield. m/s ES+ [M + H]: 254.1. Compound 2 (1.0 mmol) was dissolved in tetrahydrofuran (15 mL) in a 20 mL microwave vial. Tetrahydro-4H-thiopyran-4-one 1,1-dioxide (1.5 mmol) was added, followed by acetic acid (1.0 mmol), then sodium triacetoxyborohydride (4.1 mmol). The reaction was heated to 40 °C for 6 h via microwave irradiation. The reaction was poured into saturated aqueous sodium bicarbonate, extracted into ethyl acetate, and purified via silica gel chromatography to give compound 3 in 68% yield. m/s ES+ [M + H]: 386.2. Compound 3 (2.5 mmol) was added to a vial, followed by 3-fluoro-4-(4,4,5,5-tetramethyl-1,3,2-dioxaborolan-2-yl)-1H-pyrrolo[2,3-b]pyridine (2.7 mmol), potassium phosphate tribasic (4.9 mmol) and chloro(2-dicyclohexylphosphino-2′,4′,6′-triisopropyl-1,1′-biphenyl)[2-(2′-amino-1,1′-biphenyl)]palladium(II) (0.12 mmol, XPhos Pd G2), tetrahydrofuran (15.0 mL) and water (15.0 mL). The mixture was degassed by nitrogen bubbling, split between two 20 mL microwave vials, and heated to 100 °C for 45 min via microwave irradiation. The mixture was poured into aqueous sodium bicarbonate, extracted into ethyl acetate, the solvent removed by rotary evaporation, and residue purified by medium pressure reverse phase liquid chromatography to give compound 4 as the trifluoroacetate salt. Compound 4 was converted to the free base by treatment with sodium bicarbonate, then converted to the di-HCl salt by treatment with 2 M hydrochloric acid in diethyl ether to give a white solid in a yield of 34%. ^1^H NMR (400 MHz, DMSO-*d*_6_) δ 11.58 (s, 1H), 8.27 (dd, *J* = 4.9, 2.4 Hz, 1H), 7.82 (d, *J* = 8.3 Hz, 1H), 7.54 – 7.35 (m, 3H), 7.15 (t, *J* = 5.0 Hz, 1H), 4.33 (s, 1H), 4.10–4.04 (m, 1H), 3.17 – 3.01 (m, 5H), 2.65 (s, 1H), 2.52 (s, 2H), 2.44 – 2.29 (m, 1H), 2.21 – 2.07 (m, 4H), 2.05 – 1.98 (m, 4H), 0.63 (d, *J* = 6.8 Hz, 2H), 0.53 (d, *J* = 6.1 Hz, 4H). [M + H]^+^ : 486.3.

Methods for the kinome selectivity and published LATSi comparison table were previously published as per Morris et al.^92^. The functional biochemical kinase selectivity assessment was performed by testing the desired compounds against a panel of 370 wild-type kinases, at two concentrations, in duplicate. This was performed at Reaction Biology (Malvern, PA). Methods are as per the in vitro biochemical kinase assays below.

For the in vitro biochemical kinase assay, compounds were tested at Reaction Biology (Malvern, PA) for LATS1, LATS2, and PKB (AKT1) kinase activity in a 10-dose IC_50_ mode, with threefold serial dilution starting at 10 µM, with 10 µM ATP concentration. Compounds were evaluated using the RBC HotSpot Kinase Assay Protocol (γ-33P assay) as described previously^93^. The assays were typically run in a 10-point curve, in singlet, to produce a titration curve from which an IC_50_ value was obtained. Staurosporine was used as a control in all assays.

For NanoBRET cellular kinase assays, compounds were tested at Reaction Biology (Malvern, PA). Kinase cellular potency data were obtained for compounds, using the Reaction Biology NanoBRET™ assay protocol. HEK293 cells (ATCC) were transiently transfected with NanoLuc®-tagged LATS1, LATS2, or AKT1 fusion vectors using FuGENE HD Transfection Reagent (Promega). Transfected cells were harvested, mixed with the appropriate NanoBRET™ tracer (K-5 for LATS1/2, K-10 for AKT1), and dispensed into 384-well plates. Test compounds were delivered via Echo 550 (Labcyte Inc., Sunnyvale, CA) and incubated at 37 °C, 5% CO₂ for 1 h. NanoBRET™ Nano-Glo Substrate with Extracellular NanoLuc® Inhibitor was then added, incubated 2–3 min at room temperature, and donor (460 nm) and acceptor (600 nm) emissions were measured on an EnVision 2104 Multilabel Reader. BRET ratios were calculated as [(Acceptor ÷ Donor) – (Acceptor no-tracer ÷ Donor no-tracer)], and IC_50_ values were determined by sigmoidal dose-response fitting in GraphPad Prism. Reference compounds CTx-0294885 (LATS1/2; IC_50_ = 300.9 nM and 211 nM) and GSK690693 (AKT1; IC_50_ = 236.0 nM) were tested in duplicate in 50 µM starting concentrations with 3-fold serial dilutions.

For RNA extraction and RT-qPCR of NCGC-023 in HepG2 cells, cells were seeded in 12-well plates at a density of 0.45 × 10^6^ cells/well (1 mL/well). 24 h post-seeding, cells were treated with the indicated drugs (500X stock) or equivalent volumes of DMSO and incubated for 4 h. Total RNA was extracted using the RNeasy Mini Kit with a vacuum manifold (Qiagen). One microgram of RNA was reverse-transcribed into cDNA using SuperScript™ IV VILO™ Master Mix (Invitrogen), and the resulting cDNA was diluted 1:20 for RT-qPCR on a ViiA 7 Real-Time PCR System using TaqMan™ Fast Advanced Master Mix (Applied Biosystems). TaqMan probes used included ACTB (Hs01060665_g1) as a housekeeping control, and AMOTL2 (Hs01048101_m1), CTGF (Hs00170014_m1), and CYR61 (Hs00155479_m1) as target genes.

Acute intraperitoneal (IP) dosing studies were conducted by Charles River Laboratories (Worcester, MA). NCGC-023 was formulated in 20% 2-hydroxypropyl-β-cyclodextrin and administered via IP injection to male CD-1 mice. Male CD1 mice were dosed with 30 mg/kg NCGC-023. At the terminal time points, 4 h and 8 h, animals were euthanized by CO₂ asphyxiation, and liver samples were collected, flash-frozen in liquid nitrogen, and stored at −80 °C until analysis. For western blotting, 50–100 mg liver fragments were extracted in Tissue Extraction Buffer #1 (Invitrogen) supplemented with protease and phosphatase inhibitor cocktails (Sigma) and homogenized using a BeadRuptor24 (3 × 15 s cycles, speed 5) in 7 mL ceramic bead tubes (1.4 mm; Omni International). Clarified supernatants were diluted 1:4 in RIPA buffer (Pierce) with inhibitors and quantified using BCA assay (Promega). Thirty micrograms of lysate per sample were separated on 4–12% Bis-Tris gels and transferred to 0.45 μm PVDF membranes (Invitrogen). Proteins were detected using the following antibodies: phospho-YAP (Ser127, Cell Signaling #13008), phospho-YAP (Ser397, Cell Signaling #13619), total YAP1 (Cell Signaling #14074), and β-actin (HRP, Santa Cruz #sc-47778). Vehicle control animals were assessed at the 8 h time point only.

Pharmacokinetic studies were performed by Pharmaron, Inc. (Beijing, China) in male C57BL/6 mice (*n* = 3). Compounds were formulated in 20% aqueous 2-hydroxypropyl-β-cyclodextrin at 0.6 or 2 mg/mL and administered intraperitoneally at 3 or 10 mg/kg. Plasma samples were collected at 0.083, 0.25, 0.5, 1, 2, 4, 8, and 24 h post-dose and analyzed by liquid chromatography–tandem mass spectrometry (LC–MS/MS) using an AB SCIEX API 5500 instrument with a Waters XSelect HSS T3 2.5 µm (30 × 3.0 mm) column and internal standard quantification. Pharmacokinetic parameters were calculated via noncompartmental analysis using WinNonLin.

### Animal models

All animal procedures were approved by the Institutional Animal Care and Use Committee (IACUC) of Duke University. C57BL/6N mice were originally obtained from Charles River Laboratories and then bred at Duke University. All mice were at least 8-12 weeks old at the time of irradiation. Both male and female mice were used, and both age and sex matched littermates were used as controls for all experiments. The health status of mice was monitored by trained laboratory personnel. The criteria for euthanasia were based on decreased activity and squinted/closed eyes on a scale of 1 to 3. A mouse receiving a score of 3 in either category was considered moribund and was euthanized by carbon dioxide asphyxiation.

### Intestinal organoid culture

Mouse small intestinal organoids were cultured according to a previously described method established by Sato and Clevers^90^. Crypts were collected by incubating the small intestine in 30 mM EDTA, followed by a series of three 1X PBS washes and isolation using a 100 µm strainer (Corning, 431752) to remove the villi. Crypts were plated in phenol red-free, growth factor-reduced Matrigel (Corning, #356231) and grown in IntestiCult organoid growth medium (StemCell, #6005) until they were plated for experiments. Organoids for experiments were grown in ENR media containing Advanced DMEM/F-12 (ThermoFisher, #12634010), supplemented with 10 mM HEPES (ThermoFisher, #15630080), 2 mM GlutaMAX (ThermoFisher, #35050061), 100 U/mL Penicillin/100 mg/mL Streptomycin (ThermoFisher, #15240062), B-27 supplement (ThermoFisher, #17504044), N2 supplement (ThermoFisher, #17502048), 300 ng/mL human R-Spondin1 (StemCell Technologies, #78213.1), 1 mM N-Acetylcysteine (Sigma, #A9165), 50 ng/uL recombinant mouse EGF protein (R&D Systems, #2028-EG), and 100 ng/uL recombinant mouse noggin protein (PeproTech, #250-38). Human jejunal organoid lines J2 and J3 were purchased from Baylor College of Medicine^43,44^ and grown in IntestiCult (StemCell, #6010) for both maintenance and experiments. The Institutional Review Board at Baylor College of Medicine approved the study protocol (H-49668) to obtain tissue samples from which the organoid lines were established, and written informed consent was obtained from each donor.

### Intestinal organoid cloning/CRISPR

Plasmids and sgRNA cloning Lentiguide-puro were a gift from Feng Zhang (Addgene plasmid #52963), Lentiguide-hygro was a gift from Caroline Goujon (Addgene plasmid #139462), and pKLV2-EF1a-Cas9Bsd-W was a gift from Kosuke Yusa (Addgene plasmid #68343). Gene-specific and safe-targeting mouse sgRNA sequences were selected from the Gouda genome-wide library^94^ and a previously reported bank of safe locus-targeting sgRNAs^71^, respectively. Selected sequences are in Supplementary Table 3. Complimentary oligodeoxynucleotides with the sgRNAs and appropriate cloning overhangs were phosphorylated and annealed, then ligated into BsmbI v.2-cut and calf intestinal phosphatase-treated expression plasmids. Final ligated plasmid products were midi prepped and verified by Sanger sequencing. Final sgRNAs for use were selected after knockdown efficacy was evaluated in test 3T3 cells stably expressing pKLV2-Cas9-blast.

Lentivirus was made by transfecting 293 T cells at 70–80% confluence with a 4:3:1 molar ratio of lentiviral plasmid, psPAX2, and pMD2.G (total of 28 µg per 10 cm dish of cells) using PEI-STAR Transfection Reagent (Tocris Bioscience #7854) per the manufacturer’s guidelines. Briefly, DNA and PEI mixes were separately constructed in serum-free media, with the PEI mix containing 3-fold the amount of total DNA to be transfected. After a brief incubation, the DNA and PEI mixes were combined and rested for 15 min at RT before being added dropwise to the 293 T cells. The transfection mix was replaced with standard culture media after 8–16 h. Lentivirus was collected and filtered with an 0.4 µm syringe filter at 48 and 72 h post transfection. Pooled, harvested virus was left on ice at 4 °C overnight with 1/3 volume PEG 6000 virus concentrator and resuspended in 1X PBS at roughly 1/5 the original volume. Single-use aliquots of lentivirus were stored at –80 °C.

Organoids were transduced with lentivirus using a protocol adapted from Maru et al.^95^. A 24 well plate was washed with cold 1X PBS, then 50 µL of Matrigel was added to cover the bottom of the well. Organoids were completely dissociated and resuspended in LY media. 400 µL of organoid suspension was added to each well with 100 µL of concentrated virus and 10 µg/mL polybrene. The plate containing organoids was centrifuged for 30 min at 1750 rpm to facilitate mechanical association/partial embedding of the organoids into the matrigel layer, then returned to a 37 °C incubator. The following day, media was aspirated and an additional 50 µL of Matrigel was added on top of the matrigel/organoid layer, then submerged under 500 µL of LY media after the matrigel solidified. Antibiotic selection was performed 3 days after infection. Organoids were first transduced twice with pKLV2-Cas9 and selected with blasticidin at 15 µg/mL. Then, organoids were transduced with lentivirus bearing gene- or safe locus-targeting sgRNAs and selected with the appropriate antibiotics (3 µg/mL puromycin or 150 µg/mL hygromycin). Once antibiotic selection was complete, organoids bearing *Apc*-targeting sgRNAs were enriched for deleted mutants via biological selection with LY2090314 deficient media for roughly 7 days before returning to standard culture conditions. Similarly, organoids bearing *Trp53*-targeting sgRNAs were enriched for deleted mutants using 10 µM nutlin being added to complete media for roughly 7 days prior to returning to standard culture conditions.

Primers flanking the guide cut sites were used for PCR. Gel-purified PCR product was Sanger sequenced and chromatograms showed CRISPR-mediated indels for the targeted genes compared to the safe-targeting negative control. The genetic disruptions were quantified by Inference of CRISPR Edits (ICE) software (Synthego). Stable *Apc* KO organoids had a knockout score and indel % of 95, and stable *Trp53* KO organoids had a knockout score and indel % of 98.

### Radiation experiments

To study radiation-induced GI injury, mice were exposed to a single fraction of SBI with approximately 20% of the head and forelimbs shielded to prevent hematopoietic acute radiation syndrome^29,50,51^. Jigs were used to hold unanesthetized mice to limit movement during radiation exposure. SBI was performed with an Xstrahl CIX3 biological irradiator (Xstrahl Inc., Suwanee, GA) at Duke University. Mice were treated 40 cm from the radiation source using X-rays of 320 kV, 10 mA, and a 1 mm Cu filter. The dose rate was 3.44 cGy/second. Radiation dosimetry was performed using Thermo Luminescent Dosimeters (TLDs) that were placed inside tissue equivalent mouse phantoms. TLDs were then cross calibrated using an ion chamber that had been calibrated with a NIST traceable X-ray calibration source. Organoids were grown in 48-well plates (Corning, #3548) and irradiated with a single dose of 4 or 6 Gy using an Xstrahl CIX3 biological irradiator.

### Examine the effect of NCGC-023 on the small intestines and organoids

To inhibit LATS1/2 in vivo, NCGC-023 was prepared at a concentration of 50 mg/kg by dissolving 50 mg NCGC-023 powder into sterile DMSO (5% total volume) and 20% 2-hydroxypropyl-beta-cyclodextrin dissolved in sterile 1X PBS (95% total volume) and sterile filtered. All mice used in this study were randomized within each cage to be given vehicle or NCGC-023 at a dose of 50 mg/kg via intraperitoneal injection 24 h and 2 h (–24 h, –2 h) prior to sham-irradiation or irradiation unless stated otherwise such as in Supplementary Fig. 8. Post-IR administration of NCGC-023 was given either 24 (+24) or 96 (+96) hours post-IR at either 50 mg/kg or 40 mg/kg. 40 mg/kg NCGC-023 was prepared similarly to 50 mg/kg in which 40 mg NCGC-023 powder was dissolved in sterile DMSO (5% total volume), 20% 2-hydroxypropyl-beta-cyclodextrin was dissolved in sterile 1X PBS (95% total volume), and combined and sterile filtered.

For in vitro studies, human organoids were treated once with 1 or 3 µM NCGC-023 in human IntestiCult media for 24 h prior to irradiation. Immediately after irradiation drug was removed and replaced with IntestiCult only. Media was refreshed every 72 h until the endpoint. Organoids were imaged daily on an IncuCyte S3 using the IncuCyte organoid analysis software. For human organoids, clonogenicity was calculated based on the number of organoids that were alive when plated vs. the number of organoids alive at the endpoint based on both phenotypic and morphological differences^47,48^.

### Tissue harvest

Irradiated and unirradiated mice were sacrificed by CO_2_ asphyxiation at indicated time points post-sham IR or IR, where the proximal 12 cm (duodenum/jejunum) of the small intestine was isolated and flushed with room temperature 1X PBS pH 7.4 (Gibco, #10010023). 12 cm tissues were divided into 2 cm segments and fixed in 10% neutral buffered formalin (VWR, #89370094) for 24 h, then transferred to 70% ethanol for up to one week prior to processing. When needed, BrdU (Sigma, #B5002) dissolved in sterile 1X PBS was injected into mice via intraperitoneal injection at 100 mg/kg 2 h before euthanization.

### Tissue histology and staining

For immunohistochemistry, deparaffinized slides were placed in tris-based antigen retrieval buffer (Abcam, #ab93684), washed with 1X PBS pH 7.4 containing 0.1% Tween-20 (Sigma, # P7949), and treated with 3% hydrogen peroxide prior to blocking with 10% goat serum (Vector Labs, #S-1000-20) or rabbit serum (Vector Labs, #S-5000-20) in 1X PBS. Slides were incubated with primary antibody at 4 °C overnight against BrdU (1:500; Abcam, #ab6326), Olfm4 (1:500; Cell Signaling, #39141), active-Yap (1:1500; Abcam, #ab205270), p53 (1:200; Leica Biosystems, #NCL-L-p53-CM5p), Cleaved-Caspase 3 (1:1000; Cell Signaling, #9664). For secondary antibodies, either Goat anti-Rabbit IgG (H + L) biotinylated (1:200; Vector Labs, #BA-1000) or Rabbit anti-Rat IgG (H + L) biotinylated (1:200; Vector Labs, #BA-4000) antibody was used. VECTASTAIN Elite ABC (Vector Labs, #PK-7100) was next applied to each slide for 30 min at room temperature. For chromogenic detection 3,3’-diaminobenzidine (DAB) (Vector Labs, #SK-4100) was applied and slides were counterstained with Mayer’s hematoxylin (Sigma, #MHS32) prior to dehydration and mounting.

For sirus red staining, paraffin-embedded tissue sections were deparaffinized and immersed in Picrosirius Red Solution (VitroVivo Biotech, #VE-3017-3) for 1 h, then washed in Acidified Water (VitroVivo Biotech), #VE-3017-4 before dehydration and mounting with Micromount (Leica Biosystems, #3801731). Using QuPath software, a tissue region-of-interest was created utilizing the brush tool. Under the analyze tab, stain vector was estimated to better define stained versus unstained tissue. Next, the thresholder was created using full (0.26 µm/px) resolution, hematoxylin channel output, gaussian prefilter, and a threshold of approximately 0.12, depending on the intensity of stain.

### Image quantification

To conduct manual quantification of BrdU^+^ crypts 96 h after irradiation, a positive crypt was defined as any crypt that contained more than 10 BrdU^+^ epithelial cells 96 h after irradiation^2,96^. To conduct manual quantification of Olfm4^+^ crypts 96 h after irradiation, a positive crypt was defined as any crypt that contained more than >50% of Olfm4^+^ epithelial cells. IHC-stained slides were scanned using an Aperio GT 450 image scanner through the Duke Biorepository and Precision Pathology Core. To assess the number of individual crypt cells positive for BrdU, Active-Yap, or p53, QuPath software^97^ was used to detect and quantify the number of positive cells per cross-section on digitally scanned slides. The geometric tool was used to identify crypts from the crypt villus junction to the crypt base for positive cell analysis, and at least 3 or more small intestinal circumferences were analyzed per mouse. The number of positive cells per circumference was then averaged to get a single positive total cell count.

Mitotic figures were counted over 100 crypts, where each mitotic figure was designated as “normal” or “aberrant.” A randomly selected contiguous section of clearly demarcated 15-20 crypts was counted per H&E intestinal cross-section. Mitoses were classified as “aberrant” as previously described^50,56,98^, including tripolar, asymmetric, chromosome bridging, and chromosome lagging forms. Cells that were hyper-eosinophilic with fragmented nuclei were not counted as aberrant mitoses. The samples were quantified by a board-certified veterinary pathologist (R.B.), blinded to treatment groups.

For quantification of Il-33^+^, pHH3^+^, γH2AX^+^, or pHH3^+^/γH2AX^+^ double-positive cells, a positive cell was defined as any cell that contained a positive signal after adjustment of the background signal. For the quantification of *Clu*^*+*^ crypts/field or *Clu*^*+*^ CVJ/field (Fig. 5c, g), *Clu*^*+*^ crypts were denoted as any crypt containing >50% *Clu*^*+*^ mRNA signal, and *Clu*^*+*^ CVJ were counted as positive if any CVJ region (denoted as any crypt-villus junction) containing any Clu^+^ mRNA signal. Total crypts were counted as any crypt structure that was considered intact^29^. For the quantification of *Mt1* (Fig. 6h), *Mt1*^*+*^ cells were considered positive if at least 2 mRNA puncta were present. At least 12 images were acquired (2/cross section), and 100 crypts were counted per sample.

### Immunofluorescence (IF)

For in vitro studies, passaged organoids were plated in Nunc Lab-Tek II chamber slides (ThermoFisher, #154534) and grown for 2–4 days prior to fixation. Organoids were treated with NCGC-023 for 24 h and then fixed in warm 4% PFA (VWR, #101176014) for 20 min at room temperature, followed by permeabilization in warm 0.5% Triton X-100 (Sigma, #T8787) in 1X PBS for 25 min. Organoids were washed 3x for 20 min each with 100 mM Glycine (Sigma, #4840) in diH_2_O and blocked with a mixture of 5% bovine serum albumin (BSA) (Sigma, #A7906), 0.25% Triton X-100, and 5% goat serum (Vector Laboratories, #S-1000-20) in 1X PBS for 2 h at room temperature. Organoids were incubated in either Yap (1:100; Cell Signaling #14074) or Taz (1:100; Cell Signaling, #83669) primary antibody at 4 °C for 24 h protected from light then washed 3x for 20 min each with IF buffer containing 0.1% BSA (Sigma, #A7906), 0.2% Triton X-100 (Sigma, #T8787), and 0.05% Tween-20 (Sigma, #P7949) in 1X PBS. Organoids were incubated with Goat anti-Rabbit IgG (H + L) Highly Cross-Adsorbed Alexa Fluor 647 (1:500; ThermoFisher, #A21245) secondary antibody for 1 h at room temperature protected from light then subsequently washed with IF buffer 3X for 20 min. Chambers were removed from the slide using a razor blade and ProLong Diamond Antifade Mountant with DAPI (Invitrogen, #P36962) was used to mount coverslips. Images were acquired using a Leica Stellaris 8 and FLIM confocal microscope through Duke University’s research core.

For small intestines, tissues were de-paraffinized and heated in tris-based antigen retrieval buffer (Abcam, #ab93684), washed with 1X PBS containing 0.1% Triton X-100 (Sigma, #T8787) for 5 min twice, and blocked with 10% goat serum in 1X PBS for 30 min. Slides were incubated with Il-33 primary antibody (10ug/mL; R&D Systems, #AF3626) at 4 °C overnight and then incubated with Alexa Fluor 647 (1:500; Invitrogen, #A21447) for 1 h at room temperature or phospho-histone H3 primary antibody (1:300; Cell Signaling #9701) at 4 °C overnight and then incubated with pre-conjugated Phospho-Histone H2A.X Alexa Fluor 488 (1:500; Invitrogen, #53-9865-82) for 1 h at room temperature. Slides were then washed with 1X PBS containing 0.1% Triton X-100 twice for 5 min and mounted with ProLong Diamond Antifade Mountant with DAPI (Invitrogen, #P36962). Images were acquired using a Leica DM5500 microscope.

RNA in situ hybridization (ISH) was performed using RNAscope technology (Advanced Cell Diagnostics) and RNAscope Multiplex Fluorescent V2 Assay was used to detect fluorescence based on the manufacturer’s recommendations. To detect *Clu* mRNA, the probe Mm-Clu-C3 (ACD, #427891-C3) was used; to detect *Fgfbp1* mRNA, the probe Mm-Fgfbp1-C3 (ACD, #508831-C3) was used; to detect *Mt1* mRNA, the probe Mm-Mt1-C3 (ACD, #547711-C3) was used, and Opal 570 reagent pack (Akoya Biosciences, #FP1488001KT) was used for fluorescent detection. Images were acquired using a Leica DM5500 microscope.

### Quantification of Fgfbp1 ISH

For quantification of the percentage of *Fgfbp1⁺* cells, microscopy images (TIFF format) were processed using a Cellpose-based analysis pipeline (Supplementary Fig. 16). Images were first loaded and intensity-normalized to reduce variability arising from acquisition conditions. A region of interest corresponding to the crypt-villus junction was defined computationally as described below.

Cell segmentation was performed using a custom-trained Cellpose model configured to segment nuclei using the DAPI channel, with fixed parameters for object diameter, flow threshold, and cell probability threshold. Segmentation was therefore nuclei-based, with nuclear boundaries defined by DAPI staining. These parameters were fixed prior to analysis and kept constant across all samples and conditions to ensure comparability. Segmentation outputs were post-processed, and per-cell features, including centroid coordinates, area, and marker intensity, were extracted using region-based measurements.

*Fgfbp1* signals were detected as discrete puncta, each corresponding to an individual RNA molecule, using an intensity threshold of >20% above background signal to define a punctum as positive. Puncta were assigned to individual nuclei based on spatial overlap with the DAPI-defined nuclear segmentation masks; puncta whose centroids did not fall within any nuclear mask were excluded from analysis. Cells were classified as *Fgfbp1*-positive if they contained ≥2 puncta, which served as the threshold for positivity.

The CVJ region mask was defined using a linear algebra-based approach. A line of best fit was computed along the spatial coordinates of *Fgfbp1*-positive cells to approximate the crypt base axis. This boundary was then extended vertically, approximately 20% upward and 10% downward relative to the fitted line, to delineate the CVJ region. The percentage of *Fgfbp1*-positive cells was calculated as the number of positive cells divided by the total number of segmented cells within this region. For each sample, at least 12 images per mouse (2 images per cross-section) were analyzed using identical processing parameters.

### Long-term tissue analysis

Irradiated mice were compared to age-matched controls with no history of irradiation to account for any age-related background changes. Tissues were ordinally scored by a board-certified veterinary pathologist (R.B.) in a blinded fashion from 0 to 3 or 0 to 4 correlating to the degree of lesion severity, as appropriate for the organ. Assessment of lesion severity was referable to the presence of proliferative lesions or other chronic irradiation-related changes. Organ-specific scoring schemes are in Supplementary Data 2.

### Sample processing and library prep for Bulk RNA-sequencing

Mice were treated with 50 mg/kg NCGC-023 or Vehicle at 24 and 2 h prior to harvest, with four C57BL6/N mice per condition (2 male, 2 female). Small intestines were harvested and crypts were collected by incubating the small intestine in 30 mM EDTA, followed by a series of three 1X PBS washes and isolation using a 100 µm strainer (Corning, 431752) to remove the villi. Crypt isolates from each mouse were snap frozen, and stored at –80 °C until use. Total RNA was extracted using the RNEasy Plus Mini Kit (Qiagen, 74134), according to the manufacturer’s instructions. RNA was quantified using the Qubit RNA Broad Range Assay Kit (Invitrogen, Q10210), and 1.0 μg of total RNA was depleted of rRNA using the NEBNext rRNA Depletion Kit v2 (NEB, E7400). RNA-seq library preparation was performed using NEBNext Ultra II Directional RNA Library Prep Kit for Illumina (NEB, E7760) with dual indices according to the manufacturer’s instructions. Sequencing was performed by the Duke Sequencing and Genomic Technologies Core Facility using an Illumina NextSeq P2 XLEAP flow cell, with paired-end sequencing length of 2x50bp.

### RNA-seq analysis

FastQC (version 0.11.7) was used for assessment of sequencing quality (select metrics in Supplementary Fig. 17). Despite QC flags for sequence duplication and overrepresented sequences (consistent with residual rRNA carryover), all samples ultimately retained between 33.4 and 41.6 million transcriptome-mapped reads with Salmon (mean 38.3 million; described in Supplementary Fig. 17), providing adequate depth for our planned differential expression analysis at the gene-level. Transcripts were quantified using Salmon (version 1.10.0) and expression estimates imported into R using tximport (version 1.32.0). Transcripts were aggregated to the gene level using the Mus musculus Ensembl dataset (release 115, September 2025), retrieved via biomaRt. Genes with fewer than 10 total counts across all samples were removed prior to analysis. Differential expression between NCGC-023- and vehicle-treated groups was performed using DESeq2 (version 1.44.0) with condition as the sole covariate. Log2 fold changes were shrunk using the adaptive shrinkage estimator (ashr method, version 2.2-63) via lfcShrink^99^. Genes with an adjusted *p*-value < 0.05 (Benjamini–Hochberg) were considered significant, and up/down-regulated genes were annotated using a threshold of log2(2) (2-fold).

### RNA-seq visualization

Volcano plots were generated using ggplot2 (version 4.0.2), using ggrepel (version 0.9.6) for gene label rendering. Heatmaps were constructed using rlog-transformed expression values (DESeq2, blind = FALSE), with z-score row scaling and visualization using pheatmap (version 1.0.13) or ComplexHeatmap (version 2.20.0) with palettes from the viridis package (version 0.6.5). The heatmaps depicting signature-specific heatmaps display all genes meeting padj<0.05 that overlap with indicated gene sets. Sample quality was assessed using rlog-based principal component analysis (PCA), Pearson sample-to-sample correlation matrices, pairwise replicate scatter plots, and a heatmap of the 500 most variable genes, all computed from rlog-transformed data (Supplementary Fig. 17).

### Pathway analysis

Objective enrichment of Gene Ontology Biological Process gene sets was performed by over-representation analysis (ORA) using enrichGO from clusterProfiler (version 4.12.6) with org.Mm.eg.db (version 3.19.1) as the annotation database and all tested genes as the background universe. This analysis was performed using a gene set from our DESeq2 analysis meeting the following thresholds: adjusted *p* < 0.05 (Benjamini–Hochberg), |log2FC | >=2. ORA enrichment terms were retained if they met thresholds of adjusted *p* value < 0.10 and q-value < 0.15. Enrichment terms were initially visualized as gene-concept network plots using enrichplot (version 1.24.4) and subsequently refined in Adobe Illustrator with related biological process terms manually grouped by shared gene membership and functional similarity. All enrichment term *p*-values and component genes are described in Supplementary Fig. 12 alongside the manual grouping categories.

The same DESeq2 results were additionally interrogated to assess enrichment of specific author-selected gene signatures from the literature and other resources (see below). Rank-based gene set enrichment analysis (GSEA) performed using the fgsea (v1.30.0) R package^100^ on all expressed genes ranked by shrunken log2 fold-change using fgseaMultilevel (minSize=5, maxSize=2000, eps=0 and 10,000 initial permutations). Hypergeometric ORA was performed with phyper (one-sided, lower-tail=FALSE) using a foreground comprised of all genes passing our significance padj<0.05 threshold (*n* = 758) and a universe of 20,829 expressed genes with numerical (not NA) padj values. One-sided Fisher’s exact tests on the contingency tables were used to calculate odds ratios. *P* values from both tests were adjusted for multiple comparisons across the 24-set panel with the Benjamini–Hochberg procedure.

### Gene signatures

Referenced or publicly available gene expression signatures were retrieved across seven biological categories of signaling: p53-regulated^101,102^, NOTCH-regulated^103^, EGFR/MAPK-regulated^102,104^, FOXO-regulated (as an inverse readout of AKT signaling)^102,105^, TGFβ-regulated^106^, Wnt-regulated^102,107^, and various signatures related to Yap signaling and intestinal stem cell signatures^28,108^. MSigDB-derived sets^109^ were retrieved using msigdbr (v26.1, species = “Mus musculus”)^110^, providing 1:1 orthologue mapping for human-derived signatures if applicable.

### Statistical analysis and reproducibility

Statistical analysis was performed using GraphPad Prism 10 software. Student’s *t* test (two-tailed) was performed to compare the means of two groups, and one-way ANOVA was performed to compare the means of three or more groups. Two-way ANOVA followed by Bonferroni post hoc test was performed to examine the interaction between genotypes or drug treatment and irradiation. For survival studies, Kaplan-Meier analysis was performed, followed by the log-rank test. Significance was assumed at *p* < 0.05. Results are presented as mean ± SEM.

## Supplementary information

Supplementary Material_2026.06.27_clean.

Supplementary Data 1.

Supplementary Data 2.

## Data Availability

All data are available in the main text or the supplementary materials. The raw datasets used and/or analyzed during the current study are available from the corresponding author on reasonable request. The raw files and raw count table from the RNA sequencing can be accessed at Gene Expression Omnibus (GEO) with accession number GSE326240.

## References

[1] Dainiak, N. & Albanese, J. Medical management of acute radiation syndrome. *J. Radiol Prot*. **42**, 10.1088/1361-6498/ac7d18 (2022). 35767939

[2] Booth, C., Tudor, G., Tudor, J., Katz, B. P. & MacVittie, T. J. Acute gastrointestinal syndrome in high-dose irradiated mice. *Health Phys.* **103**, 383–399 (2012). 10.1097/hp.0b013e318266ee13 PMC3530834 23091876

[3] Freeman, M. L. Gastrointestinal acute radiation syndrome: Current knowledge and perspectives. *Cell Death Discov.* **11**, 235 (2025). 10.1038/s41420-025-02525-6 PMC12078527 40368913

[4] Prevention, C. f. D. C. a. *Acute Radiation Syndrome: Information for Clinicians*,http://www.cdc.gov/radiation-emergencies/hcp/clinical-guidance/ars.html (2024).

[5] Winters, T. A. et al. Gastrointestinal acute radiation syndrome: Mechanisms, models, markers, and medical countermeasures. *Radiat. Res.* **201**, 628–646 (2024). 10.1667/RADE-23-00196.1 PMC11658916 38616048

[6] Rios, C. I. et al. Radiation and chemical program research for multi-utility and repurposed countermeasures: A US department of health and human services agencies perspective. *Disaster Med Public Health Prep.* **18**, e35 (2024). 10.1017/dmp.2023.226 PMC10948027 38384183

[7] Kiang, J. G., Cannon, G. & Singh, V. K. An overview of radiation countermeasure development in radiation research from 1954 to 2024. *Radiat. Res.* **202**, 420–431 (2024). 10.1667/RADE-24-00036.1 PMC11385179 38964743

[8] Piccolo, S., Dupont, S. & Cordenonsi, M. The biology of YAP/TAZ: Hippo signaling and beyond. *Physiol. Rev.* **94**, 1287–1312 (2014). 10.1152/physrev.00005.2014 25287865

[9] Hong, A. W., Meng, Z. & Guan, K. L. The Hippo pathway in intestinal regeneration and disease. *Nat. Rev. Gastroenterol. Hepatol.* **13**, 324–337 (2016). 10.1038/nrgastro.2016.59 PMC5642988 27147489

[10] Yu, F. X., Meng, Z., Plouffe, S. W. & Guan, K. L. Hippo pathway regulation of gastrointestinal tissues. *Annu Rev. Physiol.* **77**, 201–227 (2015). 10.1146/annurev-physiol-021014-071733 25293527

[11] Fallah, S. & Beaulieu, J. F. The Hippo pathway effector YAP1 regulates intestinal epithelial cell differentiation. *Cells* **9**, 1895 (2020). 10.3390/cells9081895 PMC7463744 32823612

[12] Zhou, D. et al. Mst1 and Mst2 protein kinases restrain intestinal stem cell proliferation and colonic tumorigenesis by inhibition of Yes-associated protein (Yap) overabundance. *Proc. Natl. Acad. Sci. USA*. **108**, E1312–E1320 (2011). 10.1073/pnas.1110428108 PMC3241824 22042863

[13] Cai, J. et al. The Hippo signaling pathway restricts the oncogenic potential of an intestinal regeneration program. *Genes Dev.* **24**, 2383–2388 (2010). 10.1101/gad.1978810 PMC2964748 21041407

[14] Yu, F. X., Zhao, B. & Guan, K. L. Hippo pathway in organ size control, tissue homeostasis, and cancer. *Cell* **163**, 811–828 (2015). 10.1016/j.cell.2015.10.044 PMC4638384 26544935

[15] Meng, Z., Moroishi, T. & Guan, K. L. Mechanisms of Hippo pathway regulation. *Genes Dev.* **30**, 1–17 (2016). 10.1101/gad.274027.115 PMC4701972 26728553

[16] Barry, E. R. & Camargo, F. D. The Hippo superhighway: Signaling crossroads converging on the Hippo/Yap pathway in stem cells and development. *Curr. Opin. Cell Biol.* **25**, 247–253 (2013). 10.1016/j.ceb.2012.12.006 23312716

[17] Mo, J. S., Park, H. W. & Guan, K. L. The Hippo signaling pathway in stem cell biology and cancer. *EMBO Rep.* **15**, 642–656 (2014). 10.15252/embr.201438638 PMC4197875 24825474

[18] Pefani, D. E. & O’Neill, E. Hippo pathway and protection of genome stability in response to DNA damage. *FEBS J.* **283**, 1392–1403 (2016). 10.1111/febs.13604 26607675

[19] Seo, Y., Park, S. Y., Kim, H. S. & Nam, J. S. The Hippo-YAP signaling as guardian in the pool of intestinal stem cells. *Biomedicines* **8**, 560 (2020). 10.3390/biomedicines8120560 PMC7760694 33271948

[20] Camargo, F. D. et al. YAP1 increases organ size and expands undifferentiated progenitor cells. *Curr. Biol.* **17**, 2054–2060 (2007). 10.1016/j.cub.2007.10.039 17980593

[21] Barry, E. R. et al. Restriction of intestinal stem cell expansion and the regenerative response by YAP. *Nature* **493**, 106–110 (2013). 10.1038/nature11693 PMC3536889 23178811

[22] Guillermin, O. et al. Wnt and Src signals converge on YAP-TEAD to drive intestinal regeneration. *EMBO J.* **40**, e105770 (2021). 10.15252/embj.2020105770 PMC8246259 33950519

[23] Gregorieff, A., Liu, Y., Inanlou, M. R., Khomchuk, Y. & Wrana, J. L. Yap-dependent reprogramming of Lgr5(+) stem cells drives intestinal regeneration and cancer. *Nature* **526**, 715–718 (2015). 10.1038/nature15382 26503053

[24] Yui, S. et al. YAP/TAZ-dependent reprogramming of colonic epithelium links ECM remodeling to tissue regeneration. *Cell Stem. Cell* **22**, 35–49.e37 (2018). 10.1016/j.stem.2017.11.001 PMC5766831 29249464

[25] Moroishi, T. et al. A YAP/TAZ-induced feedback mechanism regulates Hippo pathway homeostasis. *Genes Dev.* **29**, 1271–1284 (2015). 10.1101/gad.262816.115 PMC4495398 26109050

[26] Ohara, T. E., Colonna, M. & Stappenbeck, T. S. Adaptive differentiation promotes intestinal villus recovery. *Dev. Cell* **57**, 166–179.e166 (2022). 10.1016/j.devcel.2021.12.012 PMC9092613 35016013

[27] Moya, I. M. & Halder, G. Hippo-YAP/TAZ signalling in organ regeneration and regenerative medicine. *Nat. Rev. Mol. Cell Biol.* **20**, 211–226 (2019). 10.1038/s41580-018-0086-y 30546055

[28] Ayyaz, A. et al. Single-cell transcriptomes of the regenerating intestine reveal a revival stem cell. *Nature* **569**, 121–125 (2019). 10.1038/s41586-019-1154-y 31019301

[29] Morral, C. et al. p53 promotes revival stem cells in the regenerating intestine after severe radiation injury. *Nat. Commun.* **15**, 3018 (2024). 10.1038/s41467-024-47124-8 PMC11001929 38589357

[30] Fink, M. et al. Chromatin remodelling in damaged intestinal crypts orchestrates redundant TGFbeta and Hippo signalling to drive regeneration. *Nat. Cell Biol.* **26**, 2084–2098 (2024). 10.1038/s41556-024-01550-4 39548329

[31] Zhu, H. et al. YAP represses intestinal inflammation through epigenetic silencing of JMJD3. *Clin. Epigen.* **16**, 14 (2024). 10.1186/s13148-024-01626-w PMC10800074 38245781

[32] Zhong, Z., Jiao, Z. & Yu, F. X. The Hippo signaling pathway in development and regeneration. *Cell Rep.* **43**, 113926 (2024). 10.1016/j.celrep.2024.113926 38457338

[33] Totaro, A., Panciera, T. & Piccolo, S. YAP/TAZ upstream signals and downstream responses. *Nat. Cell Biol.* **20**, 888–899 (2018). 10.1038/s41556-018-0142-z PMC6186418 30050119

[34] Kastan, N. R. et al. Development of an improved inhibitor of Lats kinases to promote regeneration of mammalian organs. *Proc. Natl. Acad. Sci. USA* **119**, e2206113119 (2022). 10.1073/pnas.2206113119 PMC9282237 35867764

[35] Kastan, N. et al. Small-molecule inhibition of Lats kinases may promote Yap-dependent proliferation in postmitotic mammalian tissues. *Nat. Commun.* **12**, 3100 (2021). 10.1038/s41467-021-23395-3 PMC8149661 34035288

[36] Shen, H. et al. Lats-IN-1 protects cardiac function and promotes regeneration after myocardial infarction by targeting the hippo pathway. *Front Pharm.* **15**, 1463465 (2024). 10.3389/fphar.2024.1463465 PMC11484033 39421671

[37] Namoto, K. et al. NIBR-LTSi is a selective LATS kinase inhibitor activating YAP signaling and expanding tissue stem cells in vitro and in vivo. *Cell Stem Cell* **31**, 554–569 e517 (2024). 10.1016/j.stem.2024.03.003 38579685

[38] Xiao, C. et al. Pharmacological targeting of large tumor suppressor kinases (LATS) 1 and 2 augments tissue repair and regeneration. *Br. J. Pharm.* **182**, 5536–5555 (2025). 10.1111/bph.70137 40696786

[39] Han, H. et al. Regulation of the Hippo pathway by phosphatidic acid-mediated lipid-protein interaction. *Mol. Cell* **72**, 328–340 e328 (2018). 10.1016/j.molcel.2018.08.038 PMC6195446 30293781

[40] Morris, P. J., Duveau, D. Y., Thomas, C. J., Hoyt, S. B. & Ceribelli, M. *Lats inhibitors and uses thereof*. *Patent, WO2023239727. 2023*., (2023).

[41] Ma, S. et al. Transcriptional repression of estrogen receptor alpha by YAP reveals the Hippo pathway as therapeutic target for ER(+) breast cancer. *Nat. Commun.* **13**, 1061 (2022). 10.1038/s41467-022-28691-0 PMC8881512 35217640

[42] Zachos, N. C. et al. Human enteroids/colonoids and intestinal organoids functionally recapitulate normal intestinal physiology and pathophysiology. *J. Biol. Chem.* **291**, 3759–3766 (2016). 10.1074/jbc.R114.635995 PMC4759158 26677228

[43] Saxena, K. et al. A paradox of transcriptional and functional innate interferon responses of human intestinal enteroids to enteric virus infection. *Proc. Natl. Acad. Sci. USA.* **114**, E570–E579 (2017). 10.1073/pnas.1615422114 PMC5278484 28069942

[44] Ettayebi, K. et al. Replication of human noroviruses in stem cell-derived human enteroids. *Science* **353**, 1387–1393 (2016). 10.1126/science.aaf5211 PMC5305121 27562956

[45] Poplaski, V. et al. Distinct molecular responses of human intestinal organoids to proton and photon radiation. *Am. J. Physiol. Gastrointest. Liver Physiol.* **329**, G734–G746 (2025). 10.1152/ajpgi.00186.2025 PMC12928256 41165566

[46] Montenegro-Miranda, P. S. et al. A novel organoid model of damage and repair identifies HNF4alpha as a critical regulator of intestinal epithelial regeneration. *Cell Mol. Gastroenterol. Hepatol.* **10**, 209–223 (2020). 10.1016/j.jcmgh.2020.02.007 PMC7301200 32145468

[47] Sato, T. et al. Long-term expansion of epithelial organoids from human colon, adenoma, adenocarcinoma, and Barrett’s epithelium. *Gastroenterology* **141**, 1762–1772 (2011). 10.1053/j.gastro.2011.07.050 21889923

[48] Wang, R. et al. Systematic evaluation of colorectal cancer organoid system by single-cell RNA-Seq analysis. *Genome Biol.* **23**, 106 (2022). 10.1186/s13059-022-02673-3 PMC9047329 35484598

[49] Leibowitz, B. J. et al. Ionizing irradiation induces acute haematopoietic syndrome and gastrointestinal syndrome independently in mice. *Nat. Commun.* **5**, 3494 (2014). 10.1038/ncomms4494 PMC4327858 24637717

[50] Kirsch, D. G. et al. p53 controls radiation-induced gastrointestinal syndrome in mice independent of apoptosis. *Science* **327**, 593–596 (2010). 10.1126/science.1166202 PMC2897160 20019247

[51] Lee, C. L. et al. Blocking cyclin-dependent kinase 4/6 during single dose versus fractionated radiation therapy leads to opposite effects on acute gastrointestinal toxicity in mice. *Int. J. Radiat. Oncol. Biol. Phys.* **102**, 1569–1576 (2018). 10.1016/j.ijrobp.2018.07.192 PMC6348051 30056081

[52] van der Flier, L. G., Haegebarth, A., Stange, D. E., van de Wetering, M. & Clevers, H. OLFM4 is a robust marker for stem cells in human intestine and marks a subset of colorectal cancer cells. *Gastroenterology* **137**, 15–17 (2009). 10.1053/j.gastro.2009.05.035 19450592

[53] Lopez, M. & Martin, M. Medical management of the acute radiation syndrome. *Rep. Pr. Oncol. Radiother.* **16**, 138–146 (2011). 10.1016/j.rpor.2011.05.001 PMC3863169 24376971

[54] Mah, L. J., El-Osta, A. & Karagiannis, T. C. gammaH2AX: A sensitive molecular marker of DNA damage and repair. *Leukemia* **24**, 679–686 (2010). 10.1038/leu.2010.6 20130602

[55] Leibowitz, B. J. et al. Interferon b drives intestinal regeneration after radiation. *Sci. Adv.* **7**, eabi5253 (2021). 10.1126/sciadv.abi5253 PMC8494436 34613772

[56] Leibowitz, B. J. et al. Uncoupling p53 functions in radiation-induced intestinal damage via PUMA and p21. *Mol. Cancer Res.* **9**, 616–625 (2011). 10.1158/1541-7786.MCR-11-0052 PMC3096742 21450905

[57] Capdevila, C. et al. Time-resolved fate mapping identifies the intestinal upper crypt zone as an origin of Lgr5+ crypt base columnar cells. *Cell* **187**, 3039–3055 e3014 (2024). 10.1016/j.cell.2024.05.001 PMC11770878 38848677

[58] Malagola, E. et al. Isthmus progenitor cells contribute to homeostatic cellular turnover and support regeneration following intestinal injury. *Cell* **187**, 3056–3071 e3017 (2024). 10.1016/j.cell.2024.05.004 PMC11164536 38848678

[59] Chua, J. et al. Lgr5+ stem cells maintain apex position in cell hierarchy of the intestinal epithelium during homeostasis and injury. bioRxiv. 10.1101/2025.11.09.687498 (2025).

[60] Chen, L. et al. TGFB1 induces fetal reprogramming and enhances intestinal regeneration. *Cell Stem. Cell* **30**, 1520–1537 e1528 (2023). 10.1016/j.stem.2023.09.015 PMC10841757 37865088

[61] Nusse, Y. M. et al. Publisher Correction: Parasitic helminths induce fetal-like reversion in the intestinal stem cell niche. *Nature* **562**, E22 (2018). 10.1038/s41586-018-0370-1 30013120

[62] Cheung, P. et al. Regenerative reprogramming of the intestinal stem cell state via Hippo signaling suppresses metastatic colorectal cancer. *Cell Stem. Cell* **27**, 590–604.e599 (2020). 10.1016/j.stem.2020.07.003 PMC10114498 32730753

[63] Li, Q. et al. Lats1/2 sustain intestinal stem cells and Wnt activation through TEAD-dependent and independent transcription. *Cell Stem. Cell* **26**, 675–692 e678 (2020). 10.1016/j.stem.2020.03.002 PMC7310193 32259481

[64] Han, H. et al. The Hippo pathway kinases LATS1 and LATS2 attenuate cellular responses to heavy metals through phosphorylating MTF1. *Nat. Cell Biol.* **24**, 74–87 (2022). 10.1038/s41556-021-00813-8 PMC9022944 35027733

[65] Bala, R. et al. Targeting the Hippo/YAP pathway: A promising approach for cancer therapy and beyond. *MedComm*. **6**, e70338 (2025). 10.1002/mco2.70338 PMC12394893 40895190

[66] Ghaboura, N. Unraveling the Hippo pathway: YAP/TAZ as central players in cancer metastasis and drug resistance. *EXCLI J.* **24**, 612–637 (2025). 10.17179/excli2025-8351 PMC12235270 40630468

[67] Gill, M. K. et al. A feed forward loop enforces YAP/TAZ signaling during tumorigenesis. *Nat. Commun.* **9**, 3510 (2018). 10.1038/s41467-018-05939-2 PMC6115388 30158528

[68] Zhang, H. et al. Prognostic analysis of Yes-associated protein 1 in patients with colorectal cancer. A systematic review and meta-analysis. *Rev. Esp. Enferm. Dig.* **116**, 148–156 (2024). 10.17235/reed.2022.8472/2021 36177818

[69] Yuen, H. F. et al. TAZ expression as a prognostic indicator in colorectal cancer. *PLoS One* **8**, e54211 (2013). 10.1371/journal.pone.0054211 PMC3553150 23372686

[70] Harvey, K. F., Zhang, X. & Thomas, D. M. The Hippo pathway and human cancer. *Nat. Rev. Cancer* **13**, 246–257 (2013). 10.1038/nrc3458 23467301

[71] Morgens, D. W. et al. Genome-scale measurement of off-target activity using Cas9 toxicity in high-throughput screens. *Nat. Commun.* **8**, 15178 (2017). 10.1038/ncomms15178 PMC5424143 28474669

[72] Wei, L. et al. Inhibition of CDK4/6 protects against radiation-induced intestinal injury in mice. *J. Clin. Invest* **126**, 4076–4087 (2016). 10.1172/JCI88410 PMC5096907 27701148

[73] Calafiore, M. et al. A tissue-intrinsic IL-33/EGF circuit promotes epithelial regeneration after intestinal injury. *Nat. Commun.* **14**, 5411 (2023). 10.1038/s41467-023-40993-5 PMC10480426 37669929

[74] Subramanian Vignesh, K. & Deepe, G. S. Jr. Metallothioneins: Emerging Modulators in Immunity and Infection. *Int J. Mol. Sci.* **18**, 2197 (2017). 10.3390/ijms18102197 PMC5666878 29065550

[75] Mohammed, O., Tufa, A. & Gizaw, S. T. Emerging roles of metallothioneins in human pathophysiology: A review. *Health Sci. Rep.* **8**, e71279 (2025). 10.1002/hsr2.71279 PMC12453973 40994777

[76] Kwak, S. Y. et al. Metallothionein 2 activation by pravastatin reinforces epithelial integrity and ameliorates radiation-induced enteropathy. *EBioMedicine* **73**, 103641 (2021). 10.1016/j.ebiom.2021.103641 PMC8546423 34688032

[77] Xiao, Y. et al. Hippo pathway deletion in adult resting cardiac fibroblasts initiates a cell state transition with spontaneous and self-sustaining fibrosis. *Genes Dev.* **33**, 1491–1505 (2019). 10.1101/gad.329763.119 PMC6824468 31558567

[78] Kamiya, K. et al. Long-term effects of radiation exposure on health. *Lancet* **386**, 469–478 (2015). 10.1016/S0140-6736(15)61167-9 26251392

[79] Cheng, J. et al. Anti-ceramide ScFv prophylaxis for first responders to a limited nuclear attack. *Cell Physiol. Biochem.* **58**, 418–430 (2024). 10.33594/000000721 PMC11650686 39172137

[80] Singh, V. K. et al. BIO 300: A prophylactic radiation countermeasure for acute radiation syndrome. *Mil. Med*. **189**, 390–398 (2024). 10.1093/milmed/usae156 39160790

[81] Fatanmi, O. O. et al. Prophylactic administration of BIO 300, a radiation medical countermeasure, improves erythrocyte-associated parameters in the rhesus macaque model. *Mil. Med.* **190**, 746–754 (2025). 10.1093/milmed/usaf325 40984112

[82] Kumar, V. P. et al. Pre-administration of PLX-R18 cells protects mice from radiation-induced hematopoietic failure and lethality. *Genes (Basel)* **13**, 1756 (2022). 10.3390/genes13101756 PMC9601513 36292639

[83] Holmes-Hampton, G. P. et al. PEGylated thrombopoietin mimetic, JNJ‑26366821 a novel prophylactic radiation countermeasure for acute radiation injury. *Sci. Rep.* **13**, 15211 (2023). 10.1038/s41598-023-42443-0 PMC10502090 37709916

[84] Wang, L. et al. Pre-exposure prophylaxis with vasculotide enhances survival and alleviates hematopoietic and gastrointestinal injury following lethal total body irradiation. *Int. J. Mol. Sci.* **27**, 2001 (2026). 10.3390/ijms27042001 PMC12941350 41752137

[85] Liermann-Wooldrik, K. T. et al. Identification of potential prophylactic medical countermeasures against acute radiation syndrome (ARS). *Int J. Mol. Sci.* **26**, 4055 (2025). 10.3390/ijms26094055 PMC12072061 40362293

[86] Lu, J. et al. Characterization of an in vitro 3D intestinal organoid model by using massive RNAseq-based transcriptome profiling. *Sci. Rep.* **11**, 16668 (2021). 10.1038/s41598-021-96321-8 PMC8371140 34404908

[87] Finkbeiner, S. R. et al. Transcriptome-wide analysis reveals hallmarks of human intestine development and maturation in vitro and in vivo. *Stem. Cell Rep.* **4**, 1140–1155 (2015). 10.1016/j.stemcr.2015.04.010 PMC4471827 26050928

[88] Gjorevski, N. & Ordonez-Moran, P. Intestinal stem cell niche insights gathered from both in vivo and novel in vitro models. *Stem. Cells Int* **2017**, 8387297 (2017). 10.1155/2017/8387297 PMC5610807 29081810

[89] Sato, T. et al. Paneth cells constitute the niche for Lgr5 stem cells in intestinal crypts. *Nature* **469**, 415–418 (2011). 10.1038/nature09637 PMC3547360 21113151

[90] Sato, T. et al. Single Lgr5 stem cells build crypt-villus structures in vitro without a mesenchymal niche. *Nature* **459**, 262–265 (2009). 10.1038/nature07935 19329995

[91] Taelman, J., Diaz, M. & Guiu, J. Human intestinal organoids: Promise and challenge. *Front Cell Dev. Biol.* **10**, 854740 (2022). 10.3389/fcell.2022.854740 PMC8962662 35359445

[92] Morris, P. J. et al. Discovery and optimization of LATS1 and LATS2 kinase inhibitors for use in regenerative medicine. *J. Med. Chem.* **68**, 17499–17515 (2025). 10.1021/acs.jmedchem.5c01027 PMC12406206 40768682

[93] Anastassiadis, T., Deacon, S. W., Devarajan, K., Ma, H. & Peterson, J. R. Comprehensive assay of kinase catalytic activity reveals features of kinase inhibitor selectivity. *Nat. Biotechnol.* **29**, 1039–1045 (2011). 10.1038/nbt.2017 PMC3230241 22037377

[94] DeWeirdt, P. C. et al. Genetic screens in isogenic mammalian cell lines without single cell cloning. *Nat. Commun.* **11**, 752 (2020). 10.1038/s41467-020-14620-6 PMC7005275 32029722

[95] Maru, Y., Orihashi, K. & Hippo, Y. Lentivirus-based stable gene delivery into intestinal organoids. *Methods Mol. Biol.* **1422**, 13–21 (2016). 10.1007/978-1-4939-3603-8_2 27246018

[96] Lee, C. L. et al. Assessing cardiac injury in mice with dual energy-MicroCT, 4D-MicroCT, and MicroSPECT imaging after partial heart irradiation. *Int J. Radiat. Oncol. Biol. Phys.* **88**, 686–693 (2014). 10.1016/j.ijrobp.2013.11.238 PMC3985387 24521682

[97] Bankhead, P. et al. QuPath: Open source software for digital pathology image analysis. *Sci. Rep.* **7**, 16878 (2017). 10.1038/s41598-017-17204-5 PMC5715110 29203879

[98] Donovan, T. A. et al. Mitotic figures-normal, atypical, and imposters: A guide to identification. *Vet. Pathol.* **58**, 243–257 (2021). 10.1177/0300985820980049 33371818

[99] Stephens, M. False discovery rates: A new deal. *Biostatistics* **18**, 275–294 (2017). 10.1093/biostatistics/kxw041 PMC5379932 27756721

[100] Korotkevich, G., Sukhov, V. & Sergushichev, A. Fast gene set enrichment analysis. bioRxiv. 10.1101/060012 (2019).

[101] Fischer, M. Census and evaluation of p53 target genes. *Oncogene* **36**, 3943–3956 (2017). 10.1038/onc.2016.502 PMC5511239 28288132

[102] Milacic, M. et al. The reactome pathway knowledgebase 2024. *Nucleic Acids Res.* **52**, D672–D678 (2024). 10.1093/nar/gkad1025 PMC10767911 37941124

[103] Nguyen, B. C. et al. Cross-regulation between Notch and p63 in keratinocyte commitment to differentiation. *Genes Dev.* **20**, 1028–1042 (2006). 10.1101/gad.1406006 PMC1472299 16618808

[104] Kobayashi, S. et al. EGFR mutation and resistance of non-small-cell lung cancer to gefitinib. *N. Engl. J. Med*. **352**, 786–792 (2005). 10.1056/NEJMoa044238 15728811

[105] Delpuech, O. et al. Induction of Mxi1-SR alpha by FOXO3a contributes to repression of Myc-dependent gene expression. *Mol. Cell Biol.* **27**, 4917–4930 (2007). 10.1128/MCB.01789-06 PMC1951505 17452451

[106] Karlsson, G. et al. Gene expression profiling demonstrates that TGF-beta1 signals exclusively through receptor complexes involving Alk5 and identifies targets of TGF-beta signaling. *Physiol. Genomics* **21**, 396–403 (2005). 10.1152/physiolgenomics.00303.2004 15769904

[107] Sansom, O. J. et al. Myc deletion rescues Apc deficiency in the small intestine. *Nature* **446**, 676–679 (2007). 10.1038/nature05674 17377531

[108] Mustata, R. C. et al. Identification of Lgr5-independent spheroid-generating progenitors of the mouse fetal intestinal epithelium. *Cell Rep.* **5**, 421–432 (2013). 10.1016/j.celrep.2013.09.005 24139799

[109] Subramanian, A. et al. Gene set enrichment analysis: A knowledge-based approach for interpreting genome-wide expression profiles. *Proc. Natl. Acad. Sci. USA.* **102**, 15545–15550 (2005). 10.1073/pnas.0506580102 PMC1239896 16199517

[110] Dolgalev, I. in *CRAN* (2026).

