# Supplementary material for: Prophylactic inhibition of LATS1/2 before high-dose irradiation promotes the regeneration of the damaged intestinal epithelium
Source: NPJ Regen Med. 2026 Jul 20;11:50. doi: 10.1038/s41536-026-00496-5 (PMC13751606; doi:10.1038/s41536-026-00496-5)
Supplement: Supplementary file 1 [file 41536_2026_496_MOESM1_ESM.pdf]

# SUPPLEMENTARY MATERIAL

Prophylactic inhibition of LATS1/2 before high-dose irradiation promotes the regeneration of the damaged intestinal epithelium

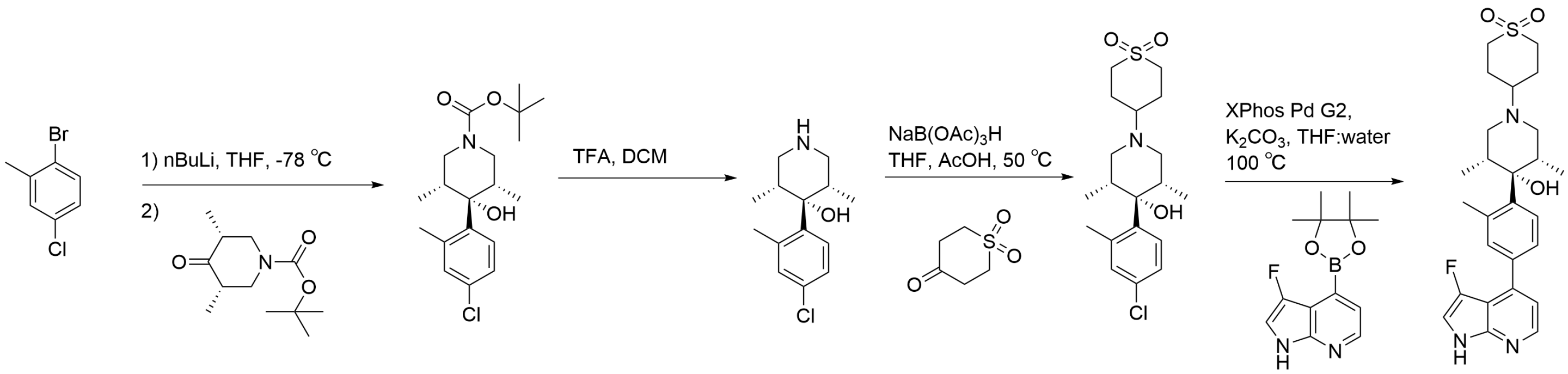


1 2 3 4

**Supplementary Figure 1. Chemical Synthesis of NCGC-023.** Overview of the steps for the chemical synthesis of NCGC-023.

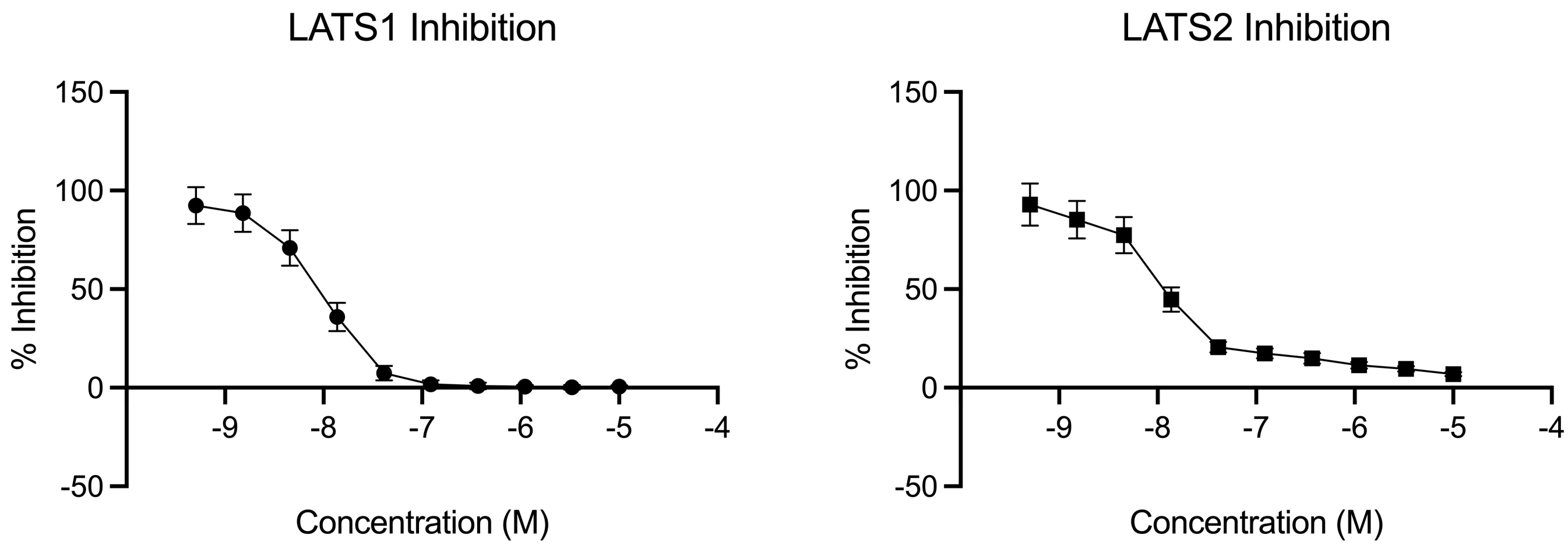


**Supplementary Figure 2.** ***In vitro*** **biochemical kinase assays of NCGC-023.** In-vitro kinase assays (γ-$^{33}$P assay) dose response curves with respect to LATS1 and LATS2 inhibition. Data are presented as mean +/- SEM (n=3 wells per group).

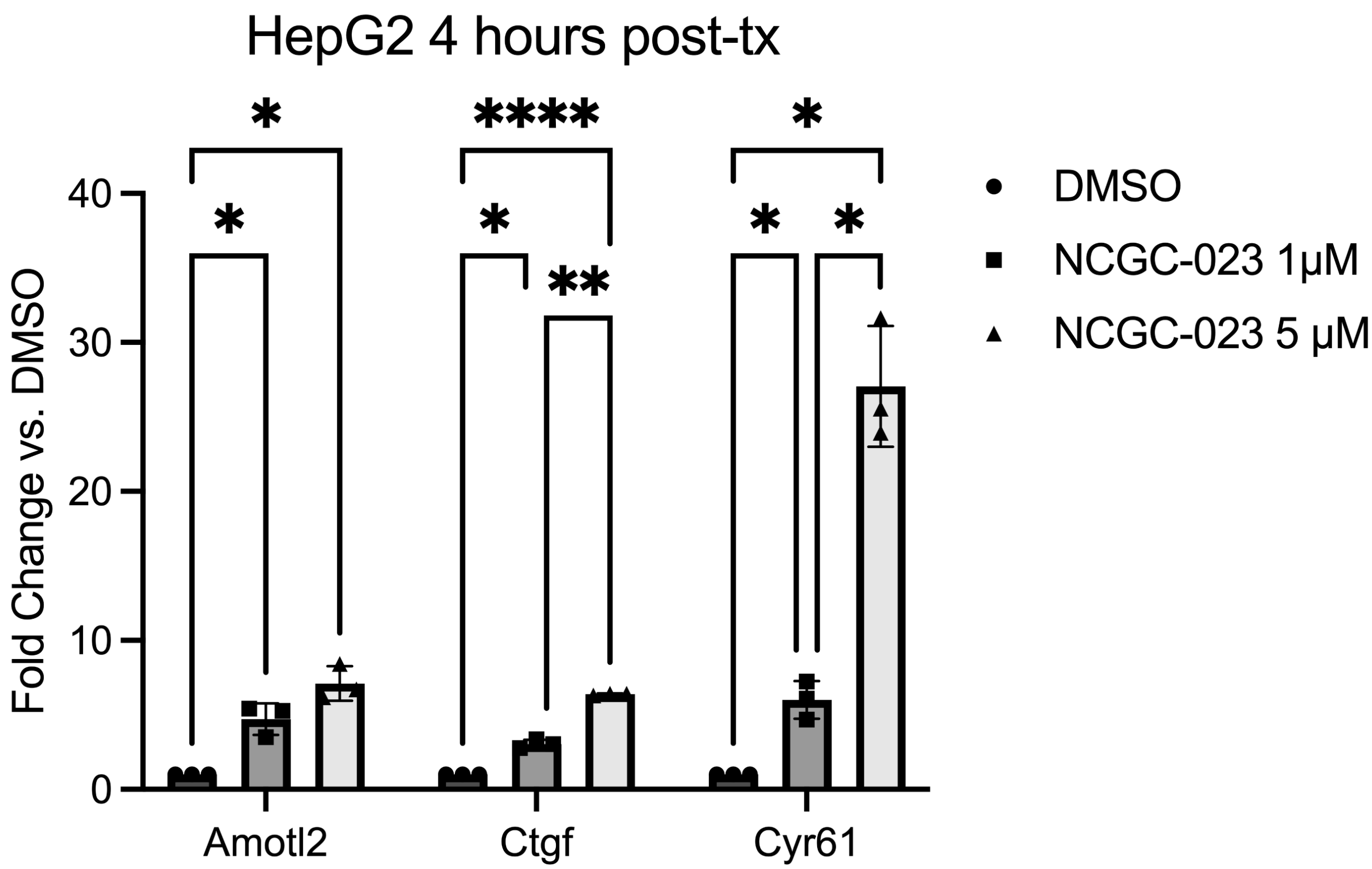


**Supplementary Figure 3. NCGC-023 treatment induces canonical transcriptional targets of *Yap* in Hepatocellular carcinoma cells.** HepG2 cells were acutely treated (4 hours) with NCGC-023, and the mRNA expression of three *bona-fide Yap1* target genes (*Amotl2, Ctgf* and *Cyr61*) were assessed by RT-qPCR. Fold Changes (FC) were calculated with respect to DMSO-treated samples. *$p<0.05$, **$p<0.01$, ****$p<0.0001$ by two-way ANOVA with Bonferroni post hoc test. Each dot represents a single well, n=3. Error bars represent SEM.

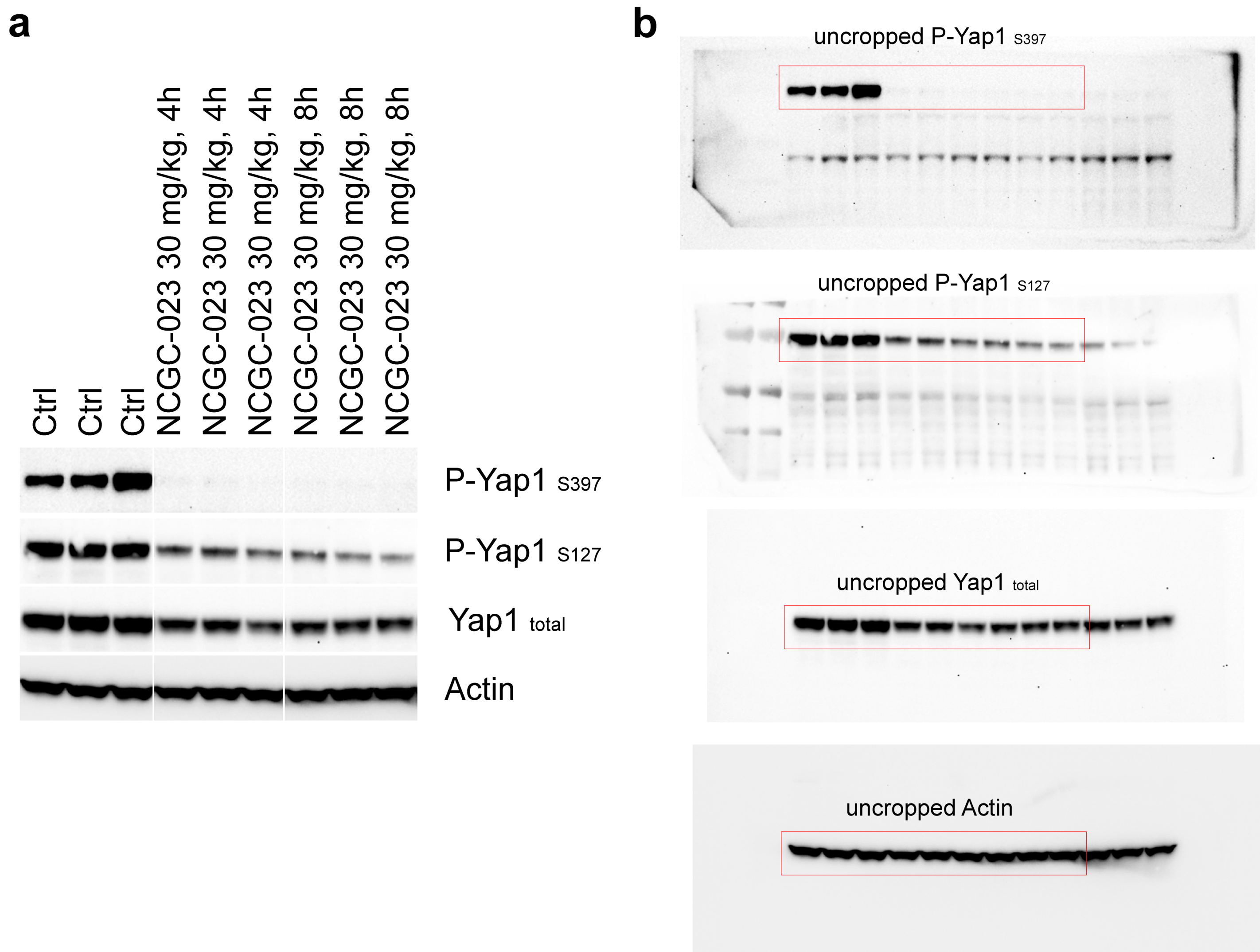


**Supplementary Figure 4. NCGC-023 treatment inhibits Yap protein phosphorylation *in vivo*. a)** Western Blot analysis of murine liver samples 4 or 8 hours following 30 mg/kg intraperitoneal administration of NCGC-023. Each lane represents one mouse. **b)** Uncropped western blot images from panel **a**.

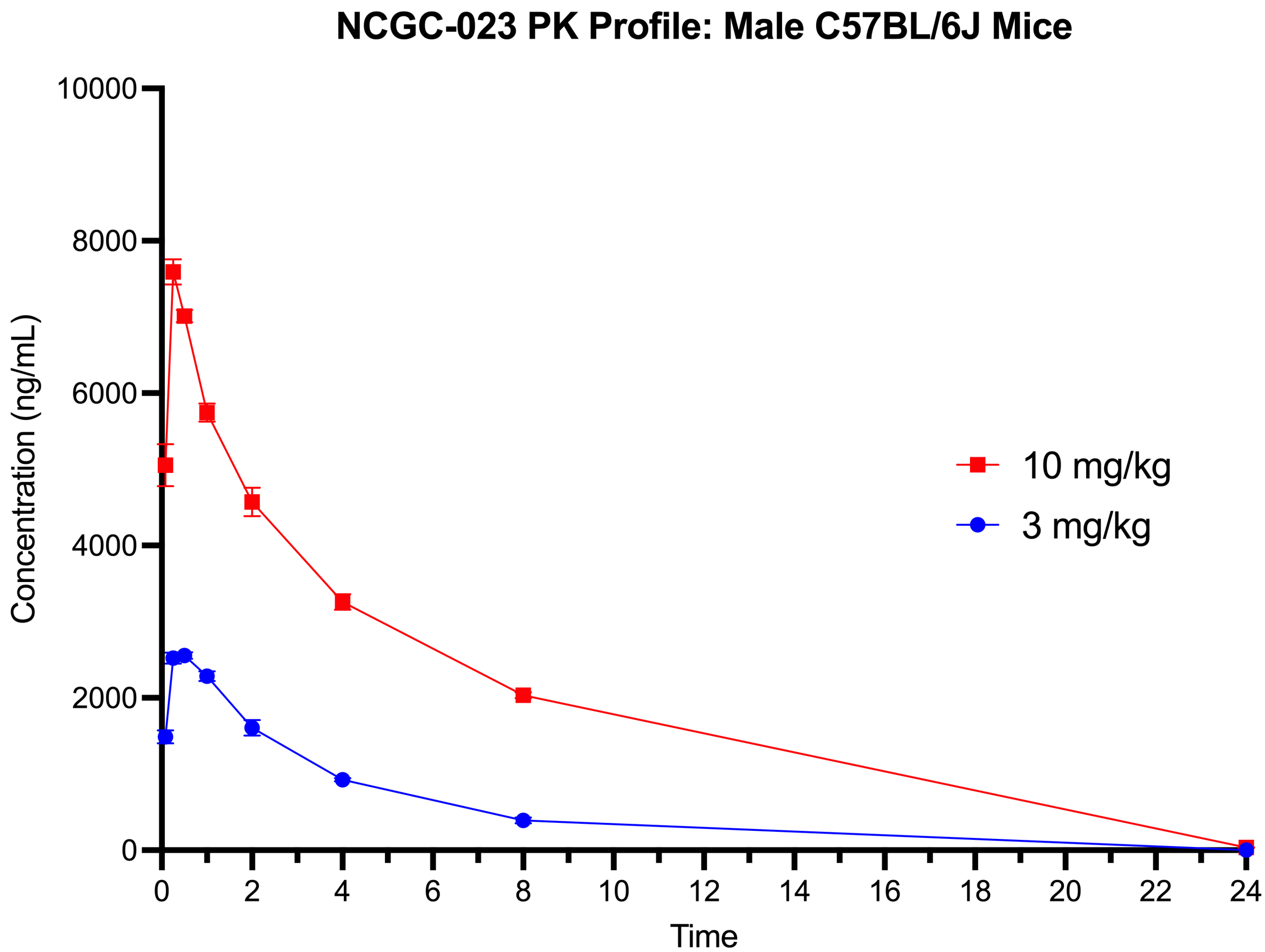


**Supplementary Figure 5. Pharmacokinetics of NCGC-023.** Plasma concentrations of NCGC-023 after intraperitoneal administration of 3 mg/kg or 10 mg/kg of NCGC-023 to male C57Bl/6J mice. n=3 mice per group. Error bars represent SEM.

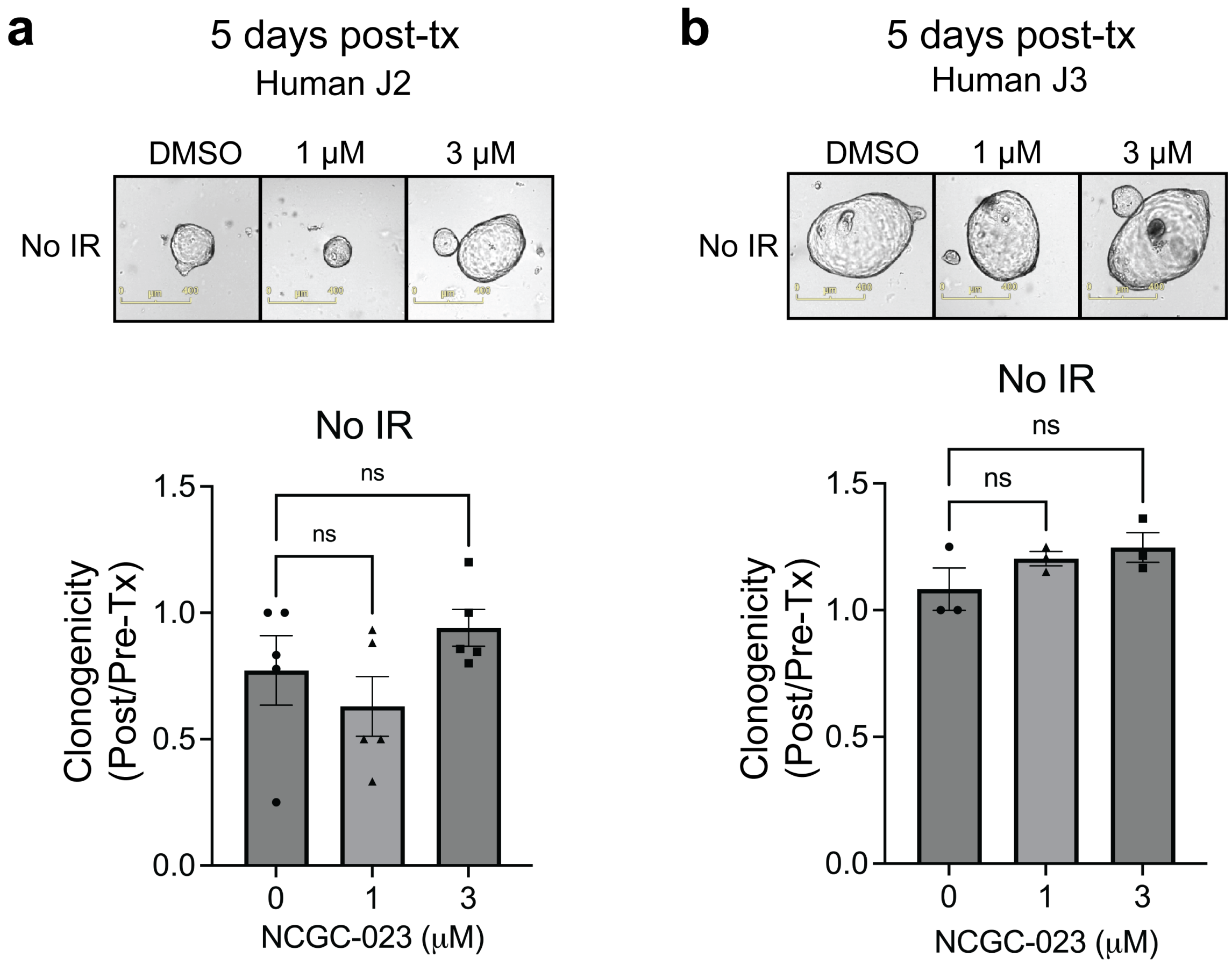


**Supplementary Figure 6. Clonogenicity of human enteroids treated with NCGC-023 without irradiation.** **a)** Unirradiated human J2 enteroids 5 days post-treatment with either DMSO, 1 μM, or 3 μM NCGC-023. Scale bar 400 μm. Quantification of the clonogenicity of unirradiated enteroid. Each dot represents a single well, n=5 wells. DMSO vs. 1 μM NCGC-023 p=0.6551, DMSO vs. 3 μM NCGC-023 p=0.5581. **b)** Unirradiated human J3 enteroids 5 days post-treatment with either DMSO, 1 μM, or 3 μM NCGC-023. Scale bar 400 μm. Quantification of the clonogenicity of unirradiated enteroid. Each dot represents a single well, n=3 wells. DMSO vs. 1 μM NCGC-023 p=0.4283, DMSO vs. 3 μM NCGC-023 p=0.2125. Error bars represent SEM. P-values were calculated by one-way ANOVA with Bonferroni post hoc test.

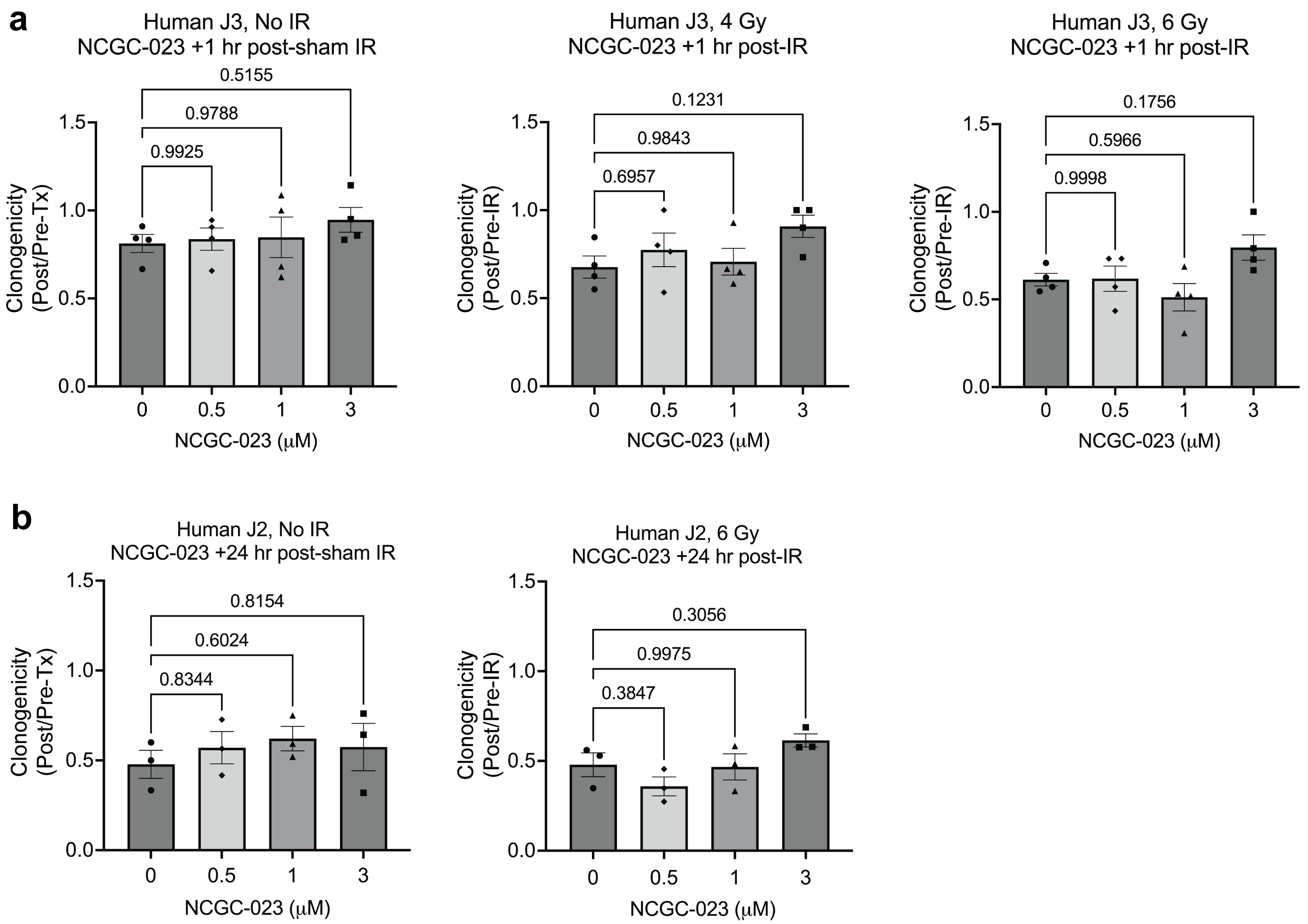


**Supplementary Figure 7. Clonogenicity of human enteroids treated with NCGC-023 post-sham IR, 4 Gy, or 6 Gy IR. a)** Human J3 enteroids were treated with DMSO, 0.5 μM, 1 μM, or 3 μM NCGC-023 1-hour post-sham, 4 Gy, or 6 Gy IR. Quantification of enteroid clonogenicity is shown. Each dot represents a single well, n=4 wells. **b)** Human J2 enteroids were treated with DMSO, 0.5 μM, 1 μM, or 3 μM NCGC-023 24 hours post-sham or 6 Gy IR. Quantification of enteroid clonogenicity is shown. Each dot represents a single well, n=3 wells. Error bars represent SEM. Numerical p-values are indicated on each graph per line. P-values were calculated by one-way ANOVA with the Bonferroni post hoc test.

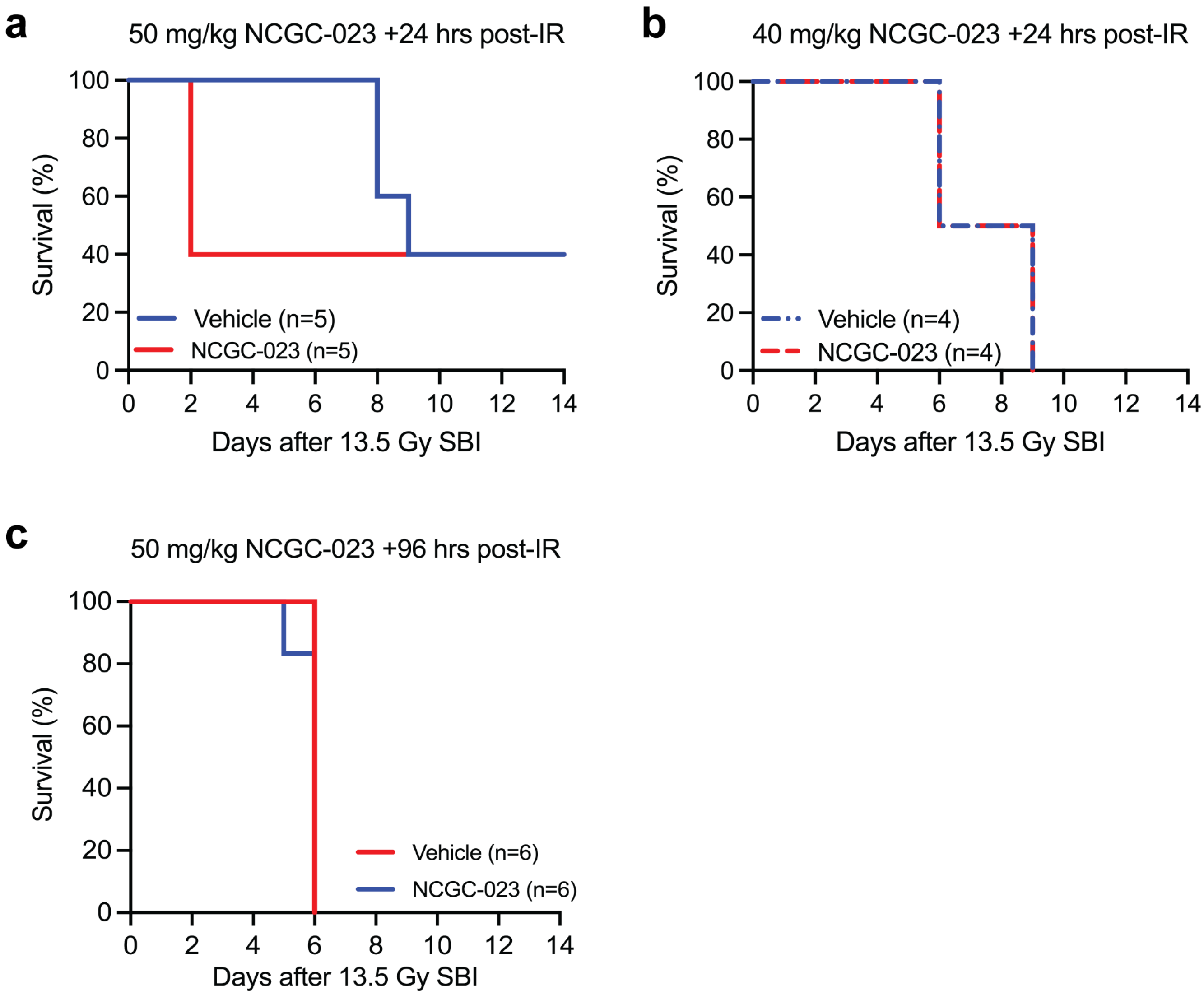


**Supplementary Figure 8. The effect of NCGC-023 administration post-irradiation on the development of GI-ARS. a)** C57BL/6N mice were treated with 50 mg/kg NCGC-023 or vehicle 24 hours post-13.5 Gy SBI and followed for the development of GI-ARS up to 14 days post-IR. n=5 mice/group, p=0.6241. **b)** C57BL/6N mice were treated with 40 mg/kg NCGC-023 or vehicle 24 hours post-13.5 Gy SBI and followed for the development of GI-ARS. n=4 mice/group, p=>0.9999. **c)** C57BL/6N mice were treated with 50 mg/kg NCGC-023 or vehicle 96 hours post-13.5 Gy SBI and followed for the development of GI-ARS. n=6 mice/group, p=0.3173. P-values were calculated by Kaplan-Meier survival analysis (log-rank test).

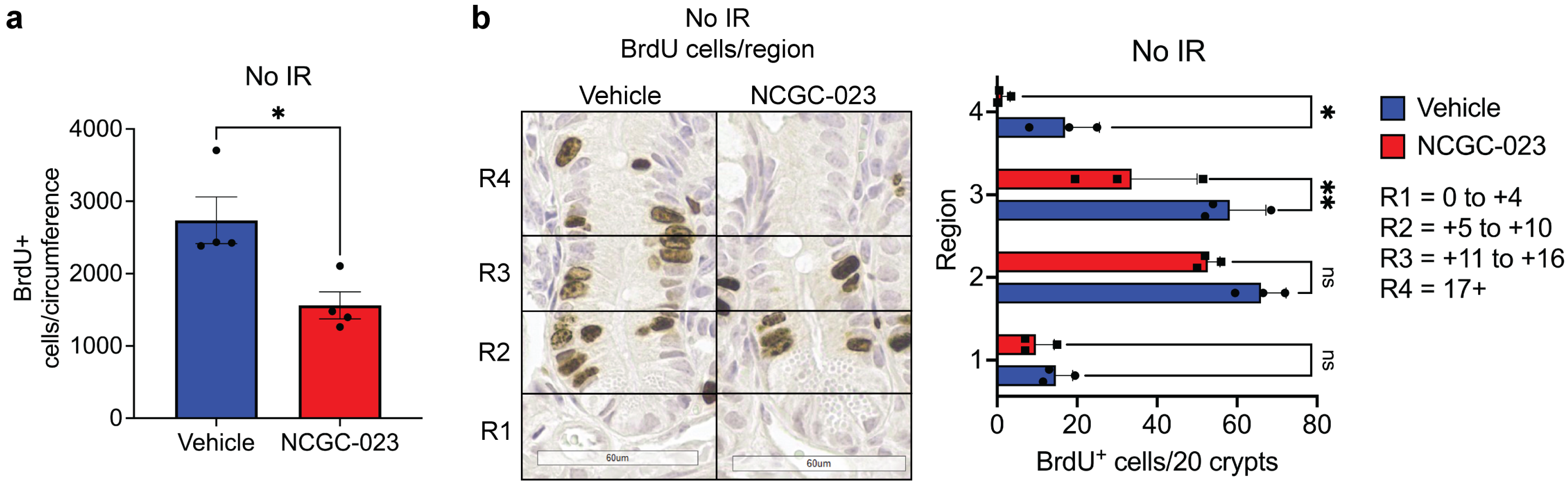


**Supplementary Figure 9. NCGC-023 reduces the proliferation of upper TA cells in unirradiated mice. a)** Quantification of BrdU$^+$ crypt cells per circumference in unirradiated mice treated with two doses of NCGC-023 or vehicle, p=0.0197. Each dot represents one mouse, n=4. Error bars represent SEM. P-value was calculated by two-tailed t-test. **b)** (Left) Representative images of BrdU$^+$ cells/region of intestinal crypts in vehicle or NCGC-023 treated mice (shown to scale). Scale bar 60 µm. (Right) Quantification of BrdU$^+$ cells/region per 20 crypts. Region 1 (R1) represents cell position 0 to +4, Region 2 (R2) represents cell position +5 to +10, Region 3 (R3) represents cell position +11 to +16, and Region 4 (R4) represents cell position 17 and above. R1 NCGC-023 vs. vehicle p=0.4668, R2 NCGC-023 vs. vehicle p=0.0760, R3 NCGC-023 vs. vehicle p=0.0057, R4 NCGC-023 vs. vehicle p=0.0436. Each dot represents one mouse, n=3. Error bars represent SEM. P-values were calculated by two-way ANOVA with the Bonferroni post hoc test.

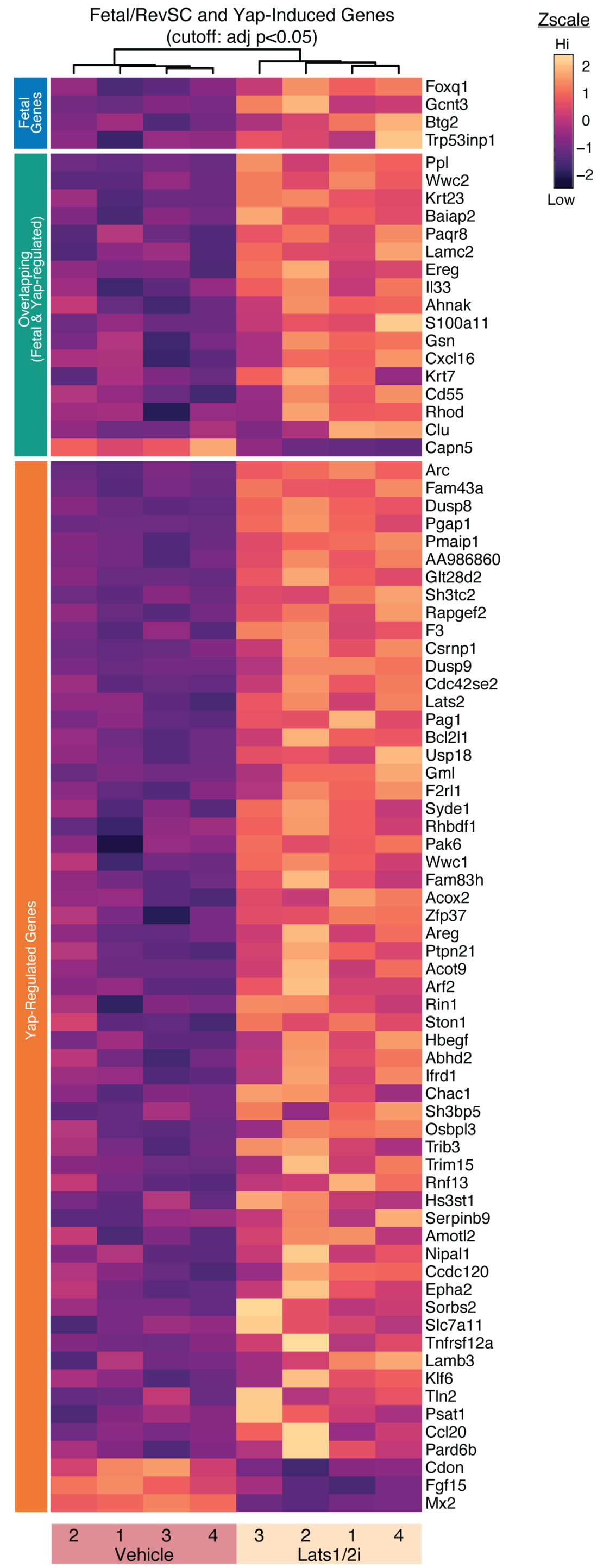


**Supplementary Figure 10. Heatmap of Fetal/RevSC and Yap-induced genes with vehicle or NCGC-023 in unirradiated intestinal crypts.** All genes within the indicated, publicly available gene signatures that also met a significance cutoff of p-value <0.05 in our data were z-scaled across the 8 samples and plotted in a heatmap, with columns ordered via unsupervised hierarchical clustering. “Fetal gene” signature (blue) was obtained from Mustata et al., “Yap regulated gene” signature (orange) was obtained from Gregorieff et al, and genes contained within both signatures are designated as “Overlapping” (green).

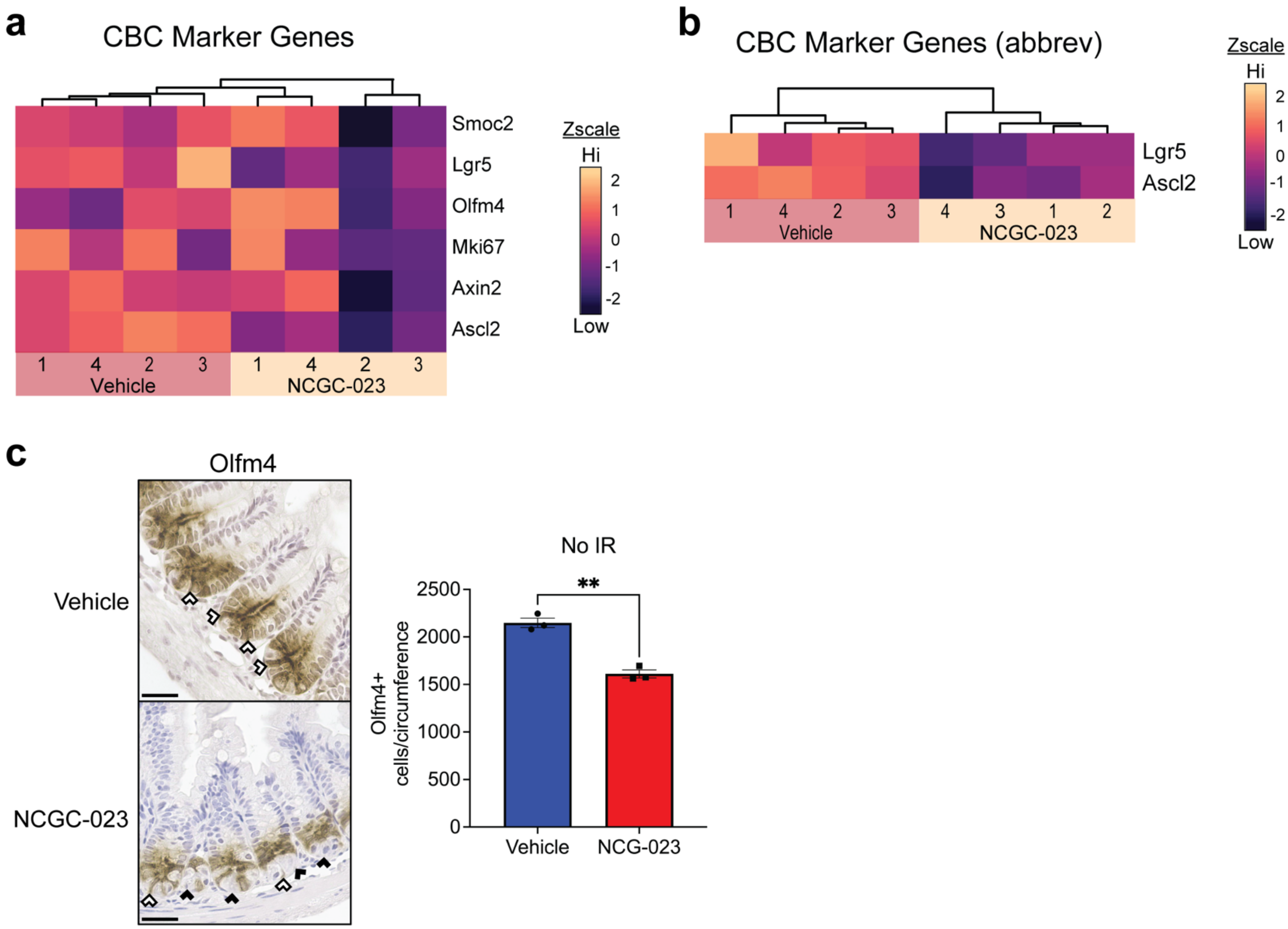


**Supplementary Figure 11. NCGC-023 downregulates canonical CBC genes in unirradiated intestinal crypts**. **a)** Canonical CBC gene expression levels were z-scaled across the 8 samples and plotted in a heatmap, with columns ordered via unsupervised hierarchical clustering. **b)** Expression levels of canonical CBC genes Lgr5 and Ascl2 were z-scaled across the 8 samples and plotted in a heatmap, with columns ordered via unsupervised hierarchical clustering. **c)** (Left) Representative images of small intestines stained for Olfm4; open white arrows indicate Olfm4-positive CBCs, black arrows indicate Olfm4-negative CBCs. (Right) Quantification of $Olfm4^{+}$ cells per circumference. Each dot represents a single mouse, p=0.0013. Error bars represent SEM. P-value was calculated by two-tailed t-test.

a

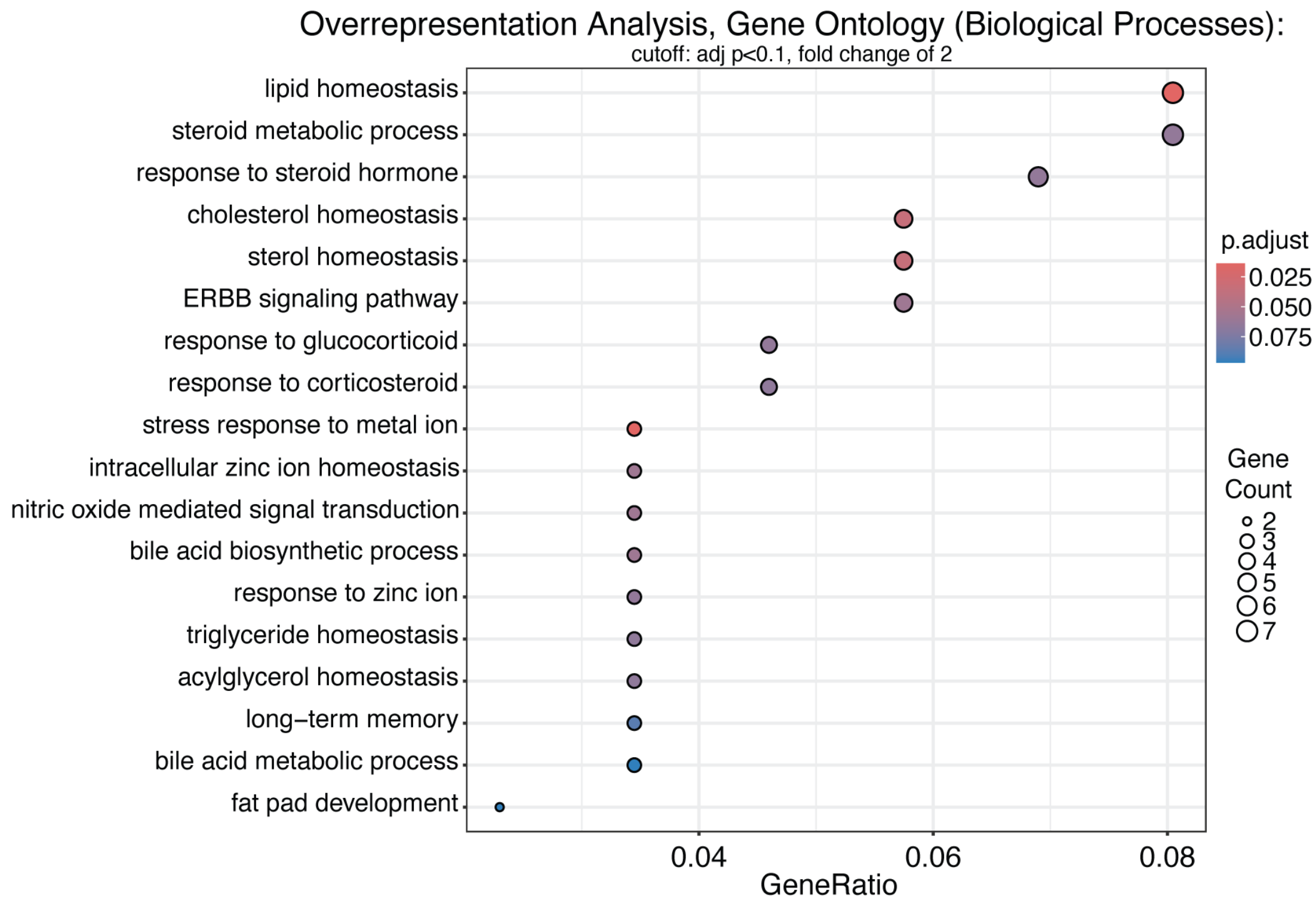


b

| ***Grouped Biological Process*** | ***Component Processes from ORA*** | ***Adjusted p-value*** | ***Shared Genes*** |
|---|---|---|---|
| Nitric Oxide Signal Transduction | 1- Nitric oxide mediated signal transduction | 0.058 | Rasd1, Mt1, Mt2 |
| Cell Response to Zinc & Metals | 2- Response to zinc ion | 0.065 | Mt1, Mt2, Slc30a10 |
| | 3- Intracellular zinc Ion homeostasis | 0.058 | Mt1, Mt2, Slc30a10 |
| | 4- Stress response to metal ion | 0.014 | Mt1, Mt2, Slc30a10 |
| ERBB Signaling | 5-ERBB signaling pathway | 0.058 | Slc30a10, Sh3tc2, Ereg, Areg, Errfi1 |
| Glucocorticoid/ Steroid Response | 6- Response to steroid hormone | 0.065 | Areg, Errfi1, Sult1a1 Per1, Ddit4, Sgk1 |
| | 7- Response to corticosteroid | 0.066 | Areg, Sult1a1, Ddit4, Sgk1 |
| | 8 - Response to glucocorticoid | 0.065 | Areg, Sult1a1, Ddit4, Sgk1 |
| Cholesterol/ Lipid Metabolism & Homeostasis | 9 - Sterol homeostasis | 0.033 | Lipg, Il18, Dgat2, Errfi1,Tsku |
| | 10 - Cholesterol homeostasis | 0.033 | Lipg, Il18, Dgat2, Errfi1,Tsku |
| | 11 - Lipid homeostasis | 0.013 | Lipg, Il18, Dgat2, Angptl4, Errfi1,Tsku, Acox2 |
| | 12 - Steroid metabolic process | 0.065 | Msmo1, Errfi1, Acox2, Fgf15,Sult1a1,Tsku, Dgat2 |
| | 13 - Bile acid biosynthetic process | 0.058 | Acox2, Errfi1, Fgf15 |
| Triglyceride & Acylglycerol Homeostasis | 14 - Triglyceride homeostasis | 0.065 | Il18, Dgat2, Angptl4 |
| | 15 - Acylglycerol homeostasis | 0.066 | Il18, Dgat2, Angptl4 |
| Long-Term Memory | 16 - Long-term memory | 0.087 | Sgk1, Arc, Lcn2 |

**Supplementary Figure 12. Analysis of pathways altered by NCGC-023 treatment in unirradiated intestinal crypts. a.** Overrepresentation analysis of biological pathways that are significantly enriched in NCGC-023-treated intestinal crypts (adj p<0.01, fold change > 2). **b.** The list of significantly enriched pathways and the associated genes.

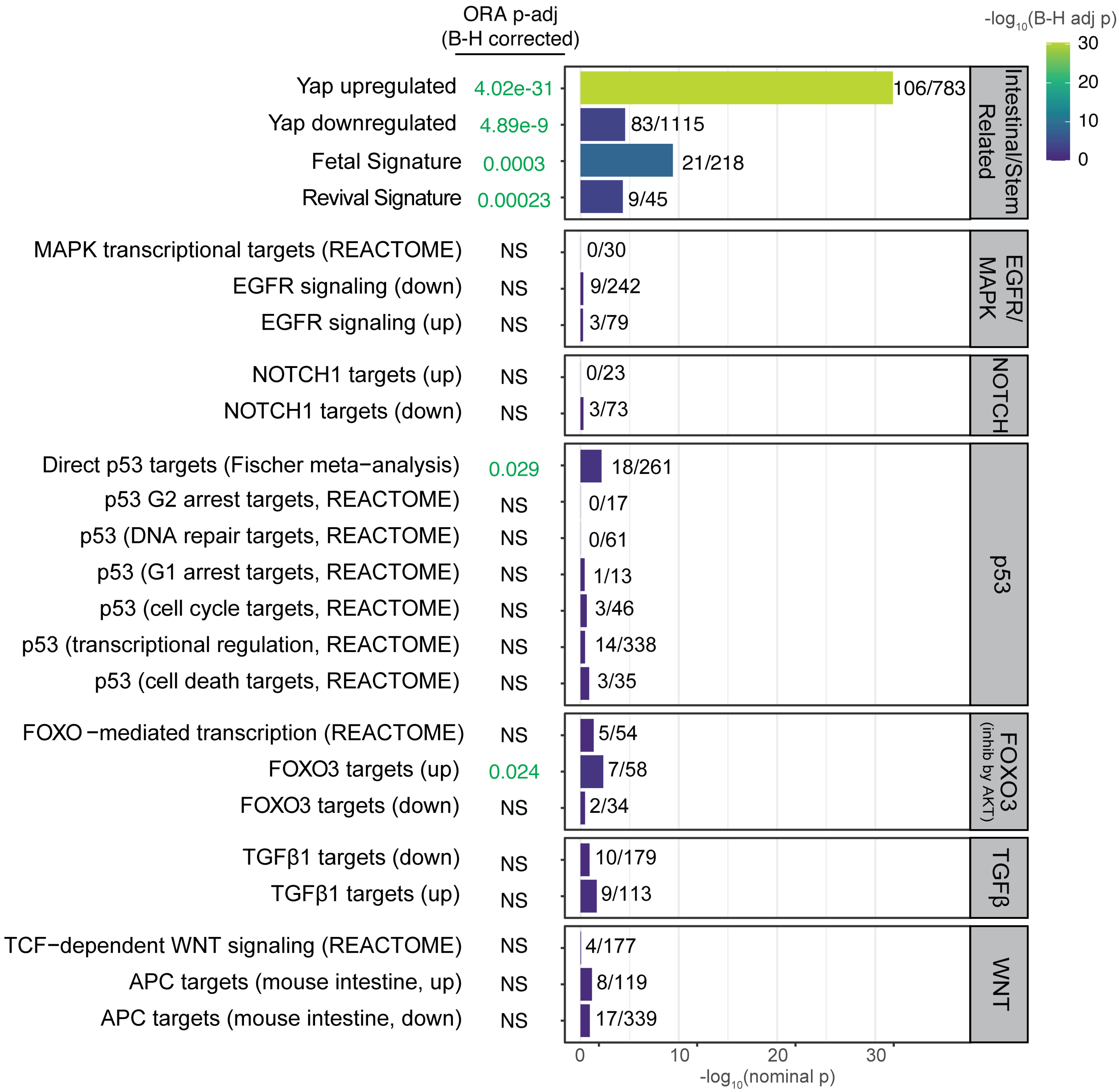


**Supplementary Figure 13. Hypergeometric over-representation (ORA) of transcriptional gene sets in NCGC-023- and Vehicle-treated mice.** The indicated gene sets were selected as representing the transcriptional output of seven pathways or signaling nodes[CEEMP1.1]. Selected gene sets were tested for enrichment using the list of 758 genes meeting our significance threshold in DESeq2 analysis. Bar length indicates nominal log10(p) from the one-sided hypergeometric test, while log10(Benjamini-Hochberg (B-H) adjusted-p value) is indicated in the viridis scaled color palette. Fraction of genes represented given next to each bar (numerator = number of set members reaching padj<0.05, denominator = number of set members in the defined gene universe for the analysis). The B-H corrected ORA nominal p-values are indicated next to the gene signature names, with signatures meeting significance indicated in green (NS=not significant).

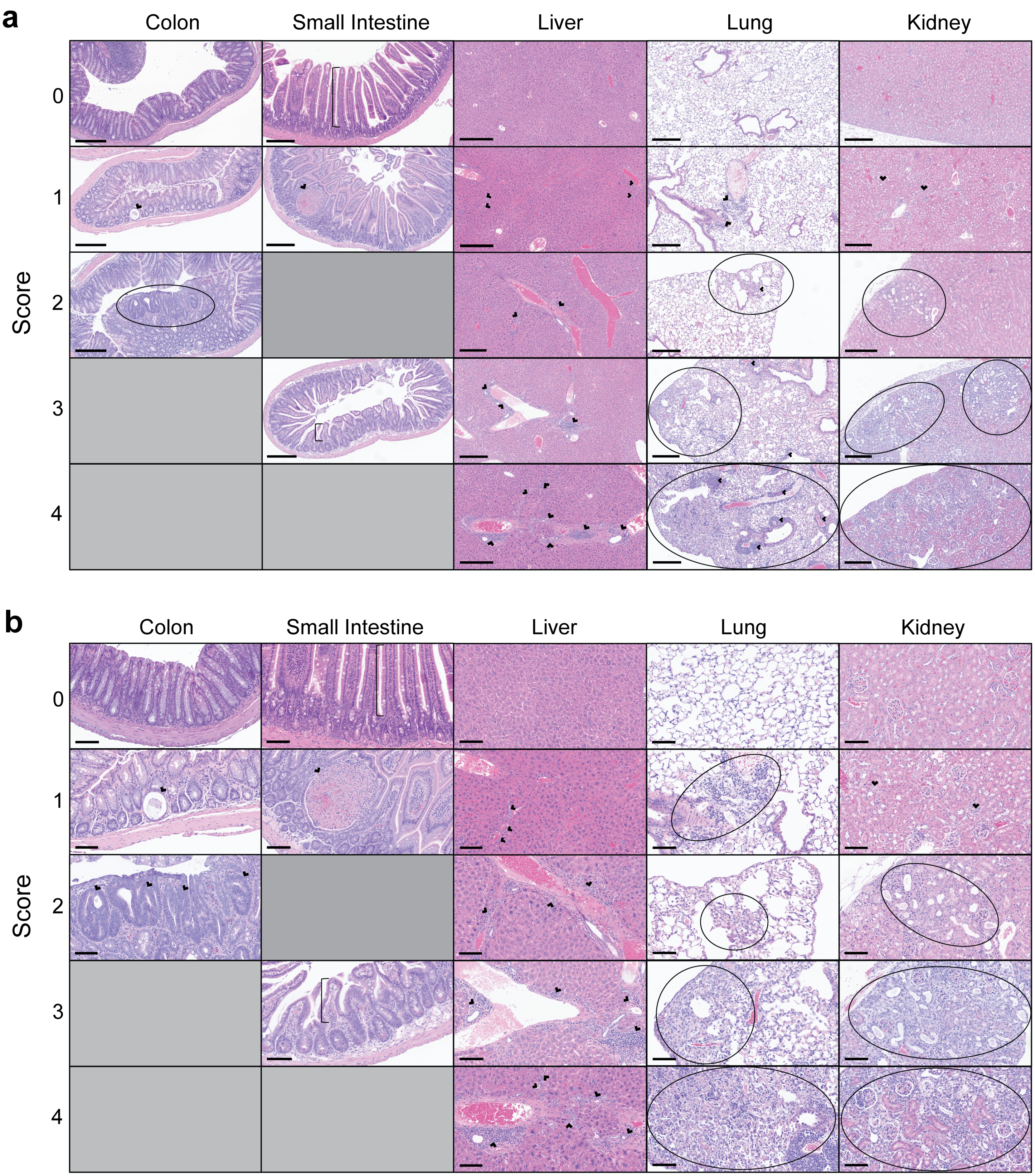


**Supplementary Figure 14. Representative images from each organ per injury score. a)** Representative images of each organ from C57BL/6N mice 4 months post-IR treated with vehicle or 50 mg/kg NCGC-023 after 13.5 Gy SBI. Each image represents pathological lesions indicated by black arrows and/or circles for representative injury scoring per organ that correlates to **Figure 7e**. Scale bar 300 µm. **b)** High magnification images from panel **a**. Pathological lesions indicated by black arrows and/or circles. Scale bar 100 µm.

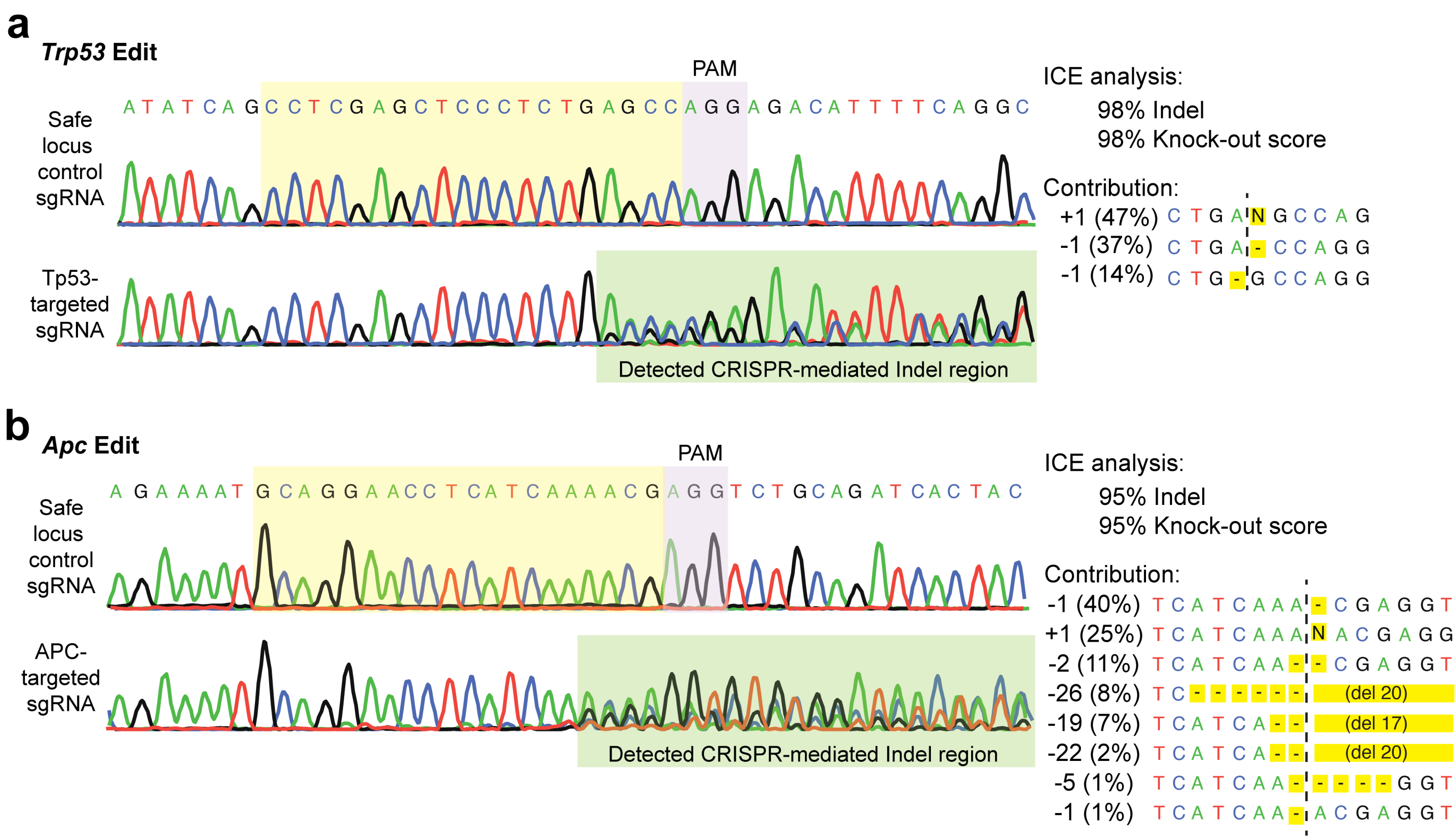


**Supplementary Figure 15. Generation of *Trp53* and *Apc* edits in mouse colonic organoids.** Stable CRISPR/Cas9-edited intestinal organoids were assessed for gene editing efficiency using PCR amplification of the edited region, followed by Sanger sequencing and Interference of CRISPR Edits (ICE) analysis. Sequencing results for *Trp53* (**a**) and *Apc* (**b**) are shown, with guide-binding region in yellow, PAM sequence in purple, and region with detected indels in green.

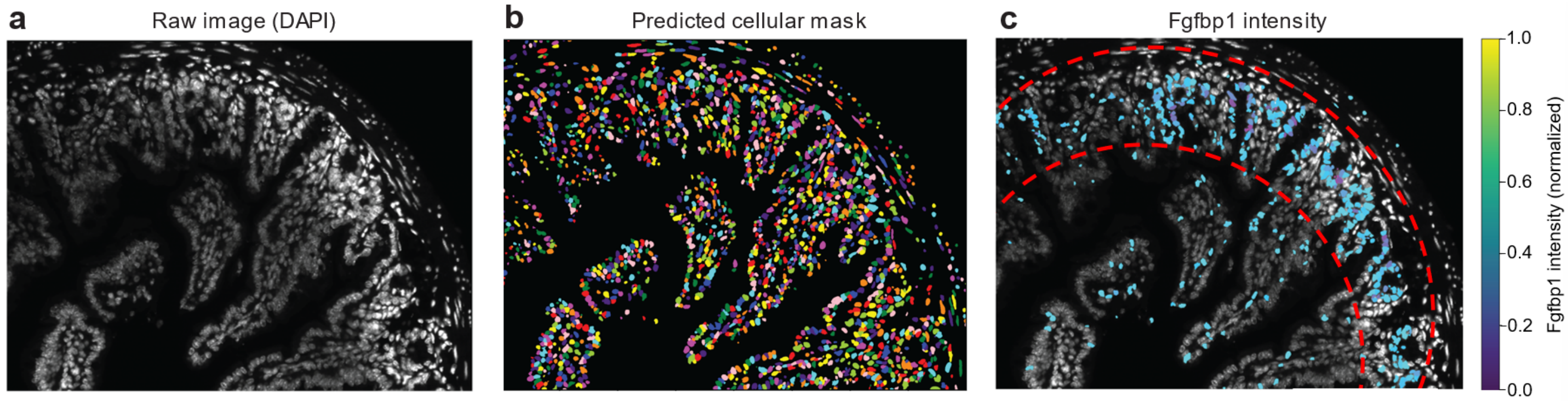


**Supplementary Figure 16. Segmentation and quantification of *Fgfbp1*+ cells. a**) Representative raw image of small intestinal tissue (DAPI channel, grayscale). **b**) Predicted cellular segmentation mask generated from the raw image. **c**) *Fgfbp1*+ cells containing ≥2 puncta overlaid on the raw tissue image and used for quantification; only cells within the crypt-villus junction to crypt base region were included (demarcated by red dotted lines).

a

| Sample | | Total Reads(M) | Mapped Reads(M) | Mapping Rate | Genes Detected |
|---|---|---|---|---|---|
| Vehicle | Rep 1 | 65.2 | 40.4 | 62.0% | 31337 |
| | Rep 2 | 66.5 | 41.6 | 62.5% | 31650 |
| | Rep 3 | 58.0 | 34.0 | 58.6% | 31854 |
| | Rep 4 | 55.1 | 33.4 | 60.7% | 31439 |
| NCGC-023 | Rep 1 | 65.7 | 39.9 | 60.8% | 31422 |
| | Rep 2 | 56.0 | 36.7 | 65.6% | 30748 |
| | Rep 3 | 65.5 | 39.9 | 60.9% | 31862 |
| | Rep 4 | 62.9 | 40.2 | 63.9% | 31742 |

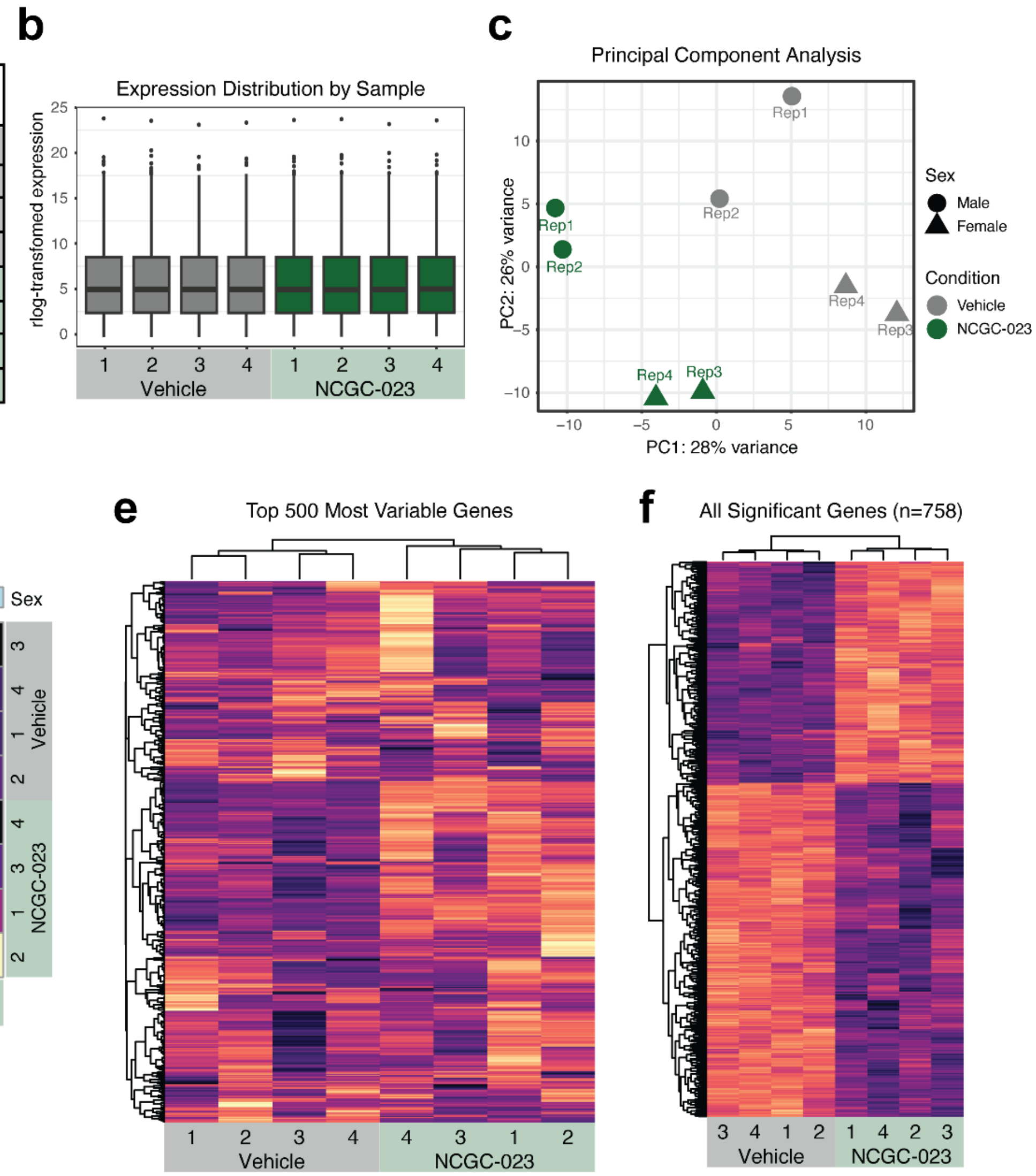


**Supplementary Figure 17. Quality control of bulk RNA-seq data. a**) Summary statistics for each sample after Salmon quantification (total reads, mapped reads, and mapping rate) and DESeq2 filtering (genes detected with raw count >0). Mapped reads and mapping rates reflect transcript-level quantification. Lower mapping rates are consistent with residual rRNA carryover following rRNA depletion, but total mapped read numbers remain appropriate for downstream analyses. **b**) Sample-specific boxplots of rlog-transformed expression values, showing comparable expression distributions across all 8 samples. **c**) Principal component analysis (PCA) of rlog-transformed expression data. Predominantly, PC1 (28% variance) separates samples by treatment condition whereas PC2 (26% variance) separates samples by sex. **d**) Pearson sample-to-sample correlation matrix with unsupervised hierarchical clustering from rlog-transformed expression values demonstrates samples cluster by treatment condition and sex; minimum sample-to-sample Pearson correlation was 0.997. **e**) Unsupervised heatmap of the 500 most variable genes, displayed as Z-scores of all rlog-transformed samples. **f**) Heatmap of all 758 genes meeting the significance threshold (adjusted p-value<0.05, no fold change cutoff) in the NCGC-023 versus vehicle condition, displayed as row Z-scores of rlog-transformed values.

## SUPPLEMENTARY TABLES

**Supplementary Table 1. *In vitro* biochemical kinase assays and NanoBRET cellular kinase assays of NCGC-023**

| Compound number | Biochemical LATS1 $IC_{50}$ (nM)[a] | Biochemical LATS2 $IC_{50}$ (nM)[a] | Biochemical AKT1 $IC_{50}$ (nM)[a] | NanoBRET LATS1 $IC_{50}$ (nM) | NanoBRET LATS2 $IC_{50}$ (nM) | NanoBRET AKT1 $IC_{50}$ (nM) | Solubility (ug/ml) | PAMPA (pH 7, $10^{-6}$ cm/sec) | Mouse microsomal Stability (min, $T_{1/2}$) |
|---|---|---|---|---|---|---|---|---|---|
| NCGC-023 | 14.1* | 14.7* | 59.1* | 367 | 57 | 19800 | 31 | 1314 | 70.2 |

*Average of 3 runs

**Supplementary Table 2. Comparison of selected published LATS inhibitors to NCGC-023**

| Name | Structure | LATS1 *in vitro* $IC_{50}$ (nM)[a] | LATS2 *in vitro* $IC_{50}$(nM)[a] | LATS1 cellular $IC_{50}$ (nM)[b] | LATS2 cellular $IC_{50}$ (nM)[b] |
|---|---|---|---|---|---|
| NCGC-023 | 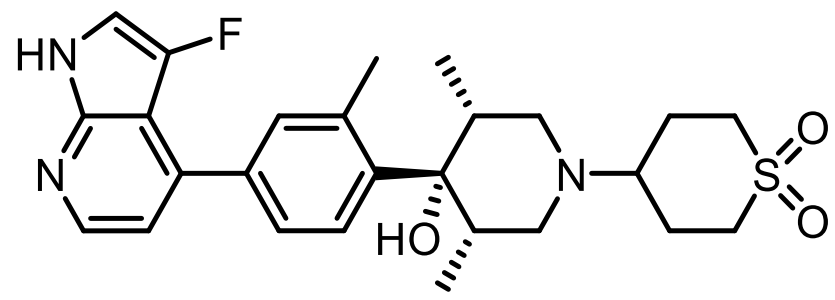 | 14.1 | 14.7 | 367 | 57 |
| TRULI | 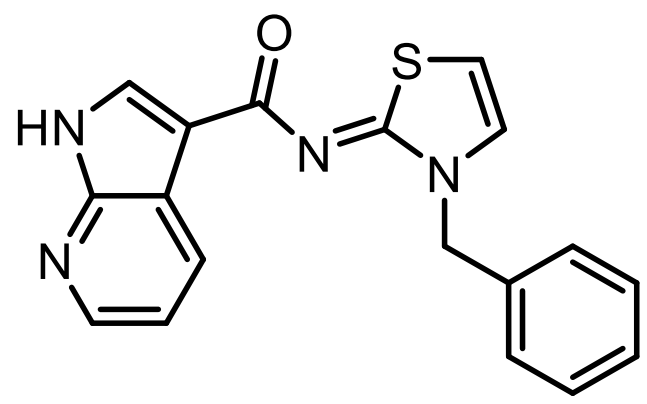 | 5.1 | 12.3 | 3840 | 795 |
| TDI-011536 | 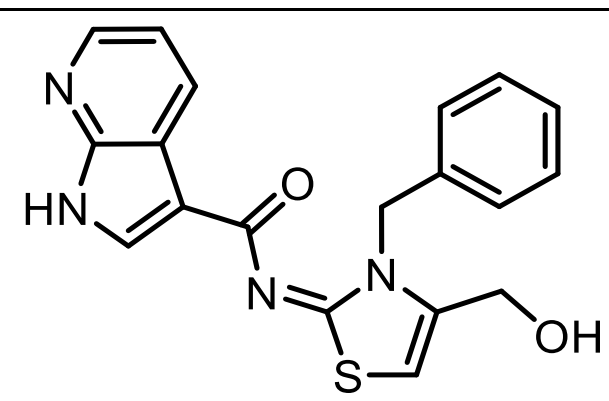 | 1.7 | 1.2 | 923 | 173 |
| VT02956 | 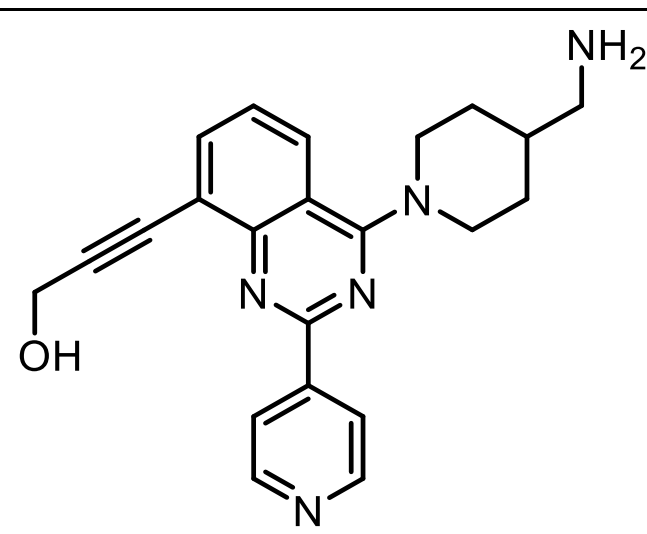 | 6.9 | 1.2 | 1980 | 773 |
| NIBR-LTSi (racemate) | 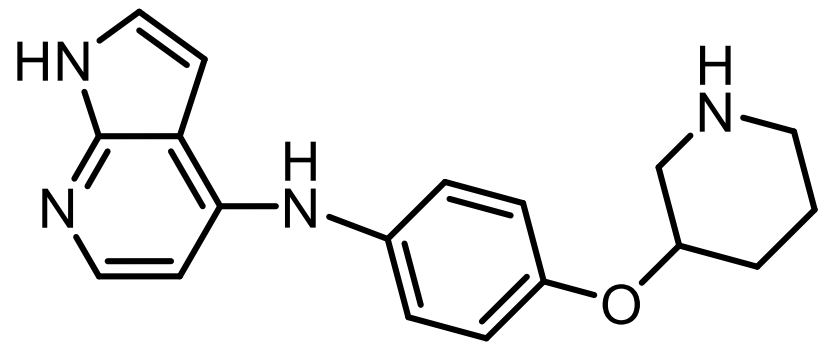 | 1.5 | 2.8 | 3580 | 139 |

[a] Biochemical $IC_{50}$ determined via Y-$^{33}$P functional assay.
[b] Cellular $IC_{50}$ determined by NanoBRET assay.

***Sourcing and Synthesis for selected commercial and published LATS inhibitors*.**
TRULI was purchased from Enamine. TDI-011536 was purchased from MedChemExpress. VT02956 and NIBR-LTSi, were synthesized at the National Center for Advancing Translational Sciences according to published methods. The NIBR-LTSi racemate was utilized for comparison studies.

**Supplementary Table 3. Mouse sgRNA sequences used for CRISPR/Cas9 editing**

| Name | sgRNA Sequence |
|---|---|
| Control guide | GTTCATTTCTAATGCTGGGG |
| *Apc* Knockout (KO) | GCAGGAACCTCATCAAAACG |
| *Trp53* Knockout (KO) | CCTCGAGCTCCCTCTGAGCC |

**Supplementary Data 1. NCGC-023 Kinome Selectivity**

Selectivity profiling of NCGC-023 across the human kinome, highlighting on-target activity and off-target engagement to assess therapeutic specificity.

**Supplementary Data 2. Long Term Lesion Scoring Chart**

Histological examination and scoring system from organs within the radiation field 4 months following 13.5 Gy SBI. Scoring was used at a 0-3 scale (Liver, Small Intestine, and Colon) and 0-4 (Kidney and Lung) scale to evaluate long-term tissue response and radiation-induced pathological changes in comparison to unirradiated control organs.
